# Metal‐Catalyzed Abiotic Cleavage of C═C Bonds for Effective Fluorescence Imaging of Cu(II) and Fe(III) in Living Systems

**DOI:** 10.1002/advs.202412407

**Published:** 2025-01-09

**Authors:** Chunfei Wang, Dandan Chen, Zixiang Wei, Jingyun Tan, Changfeng Wu, Xuanjun Zhang

**Affiliations:** ^1^ Faculty of Health Sciences University of Macau Macau SAR 999078 China; ^2^ Department of Pharmacology School of Pharmacy Wannan Medical College Wuhu Anhui 241002 China; ^3^ Department of Biomedical Engineering Southern University of Science and Technology Shenzhen Guangdong 518055 China; ^4^ MOE Frontiers Science Centre for Precision Oncology University of Macau Macau SAR 999078 China

**Keywords:** abiotic cleavage of C═C bonds, Cu(II)/Fe(III) catalyzed reaction, fluorophore activation, living systems

## Abstract

Imaging abnormal copper/iron with effective fluorescent tools is essential to comprehensively put insight into many pathological events. However, conventional coordination‐based detection is mired in the fluorescence quenching induced by paramagnetic Cu(II)/Fe(III). Moreover, the strong chelating property of the probe will consume dissociative metal ions and inevitably interfere with the physiological microenvironment. Here, a new strategy is developed by employing this aberrant Cu(II)/Fe(III) to catalyze bond cleavage for fluorescent imaging of them. A short series of near‐infrared fluorescent molecules (**NIRB1−NIRB6**) is devised as substrates, wherein the specific C═C bonds can be effectively cleaved to activate red fluorophore by Cu(II)/Fe(III) catalyzing. Representatively, **NIRB1** is applied for fluorescent imaging of Cu(II)/Fe(III) in living cells, zebrafish, and Alzheimer's disease (AD)‐afflicted mouse brains which is of significance to monitor metal safety. The successful cleavage of C═C bonds catalyzed by Cu(II)/Fe(III) enriches the application of abiotic bond cleavage reactions in metal detection, and may also inspire the development of fluorescent tools for the future diagnosis and therapy of diseases.

## Graphical abstract

Cu(II)/Fe(III)‐catalyzed oxidative cleavage of C═C bonds is successfully achieved, resulting in activating red fluorophore. Representatively, the amphiphilic molecule can effectively cleave the specific C═C bonds in living cells and zebrafish, especially in AD‐afflicted mouse brains, wherein turn‐on fluorescence is desirable for in vivo imaging‐guided explorations in the presence of paramagnetic metals.

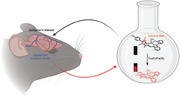

## 1 Introduction

Industrial and agricultural activities have increased people be exposed to toxic metals. Copper and iron are essential trace elements for the body, but high‐level inside can also cause serious diseases, including cirrhosis, pulmonary edema, cancers, and the like.^[^ ^1^ ^]^ Furthermore, abundant Cu(II)/Fe(III) has universally been accepted as an important pathological feature in neurodegenerative diseases, like Alzheimer's disease (AD), Parkinson's disease, and the like.^[^ ^2^ ^]^ In Vivo Imaging Systems (IVIS) could provide desirable fluorescent imaging to noninvasively track Cu(II)/Fe(III) under pathophysiological conditions. However, a better understanding of how copper/iron impacts the function of these diseases has still not been thoroughly settled for lacking effective fluorescent probes. Meanwhile, the complicated biological microenvironment and the fluorescence quenching effect by paramagnetic Cu(II)/Fe(III)^[^ ^3^ ^]^ also restrict the development of in situ fluorescent imaging of Cu(II)/Fe(III).

**Figure 1:**
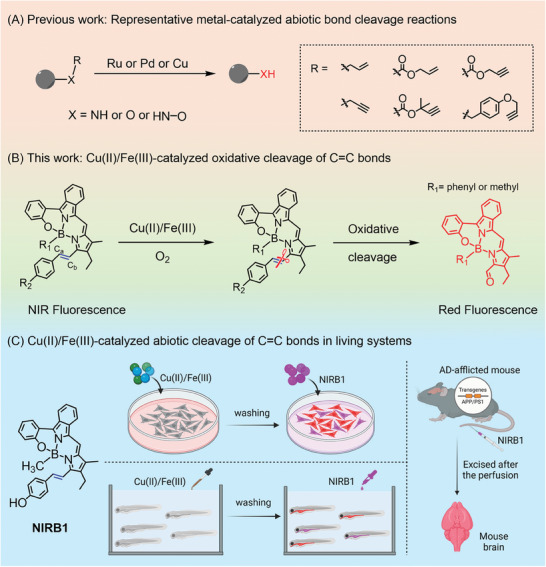
Schematic illustration of the strategy by Cu(II)/Fe(III) catalyzing abiotic bond cleavage. A) Representative metal‐catalyzed abiotic bond cleavage reactions. B) The scheme of Cu(II)/Fe(III)‐catalyzed oxidative cleavage of C═C bonds. C) Schematic illustration showing abiotic cleavage of C═C bonds from **NIRB1** catalyzed by Cu(II)/Fe(III) in living cells, zebrafish, and AD‐afflicted mouse brain.

As well known, catalysis has the function of amplification and sustainability. Compared with other transition metals, Cu(II)/Fe(III) are inexpensive and have realized Suzuki−Miyaura cross‐coupling, Sonogashira coupling, and many diversified click reactions,^[^ ^4^ ^]^ which have largely cantered over the past decades. Presently, metal‐catalyzed abiotic chemical reactions have rapidly been emerging as the driving force for activating small molecules within specific organisms in medicinal chemistry and chemical biology, which transformed inactivated/protected molecules to their corresponding products with activity^[^ ^5^ ^]^ (**Figure** **1**A). Unlike general chemical reactions, abiotic chemical reactions should proceed in elusive biological systems without disturbing native physiological processes. Therefore, they can successfully dissect biological processes in the context of living cells or whole organisms, including specific fluorescent labeling, microenvironment monitoring, in situ synthesis of drugs, and removal of toxic species.^[^ ^6^ ^]^ For instance, Chen and co‐workers did excellent work on the activation of protein and prodrugs in living systems by utilizing abiotic bond cleavage.^[^ ^7^ ^]^ Bradley and co‐workers also contributed to activating drugs within biological systems via Pd‐catalyzed bond cleavage.^[^ ^8^ ^]^ Even though they are powerful, the most prominent has been limited in practice to traditional decaging or deprotection of allyloxycarbonyl, propargyloxycarbonyl, allyl and propargyl.^[^ ^9^ ^]^ Furthermore, the intracellular synthesis of designed products from exogenous reactants is also very important.

Currently, nearly all abiotic bond cleavage reactions as a new emerging part of intracellular synthesis in living systems, were strived to develop biocompatible metal catalysts for decaging and deprotection.^[^ ^10^ ^]^ Delightfully, numerous metal catalysts have been successfully prepared as nano‐ and meso‐structures to improve biocompatibility and avoid side effects or toxicity.^[^ ^11^ ^]^ Specifically, enamines with β‐vinylic hydrogens were once reported to be mostly α‐oxygenation by Cu(II) resulting in exclusive cleavage of C═C bonds, which was related to ^1^O_2_ and displayed a metal reactivity trend of copper > iron > cobalt ≫ nickel.^[^ ^12^ ^]^ Rather than exploring new catalysts, we here utilized intrinsic Cu(II)/Fe(III) elevated in living systems as catalysts, within which to explore abiotic bond cleavage reactions for fluorescent imaging. Herein, we rationally devised and synthesized a short series of near‐infrared (NIR) emitting molecules as substrates, wherein the C_a_═C_b_ bonds could be oxidatively cleaved by Cu(II)/Fe(III) yielding red‐emitting products (Figure 1B). Activated red fluorescence was not quenched by paramagnetic Cu(II)/Fe(III), so the effective reaction in living systems was simultaneously tracked by fluorescence imaging (Figure 1C). Meaningfully, Cu(II)/Fe(III) in living systems acted as catalysts, avoiding the time‐consuming and laborious construction of exogenous catalysts, as well as side effects and toxicity induced by introducing them. The possibilities of the C═C bond cleavage catalyzed by aberrant Cu(II)/Fe(III) in living systems not only expands the diversity of abiotic chemical reactions but also opens an avenue to catalysis for the deep understanding of diseases.

## 2 Results

### 2.1 Design and Synthesis of NIR‐Emitting Molecules

Oxidative cleavage of the $C_{{\mathrm{sp}}^{2}}-C_{{\mathrm{sp}}^{2}}$ bond is one of the most common reactions in organic chemistry, wherein metal salts or complexes have been preferentially utilized as catalysts.^[^ ^13^ ^]^ Especially, the flexible interconversion between oxidation states of polyvalent metals, such as copper and iron, ensures that they can catalyze the oxidation of many substrates.^[^ ^12^, ^14^ ^]^ Moreover, copper/iron serves as co‐factors of many enzymes involved in biochemical reactions in living systems and they can be released and become free to catalyze.^[^ ^15^ ^]^ However, due to the high bond energy, abiotic oxidative cleavage of C═C bonds in living systems has been rarely achieved. We proposed Cu(II)/Fe(III)‐catalyzed oxidative cleavage of the $C_{{\mathrm{sp}}^{2}}-C_{{\mathrm{sp}}^{2}}$ bond employing NIR emitting molecules as substrates. We hypothesized that π‐conjugation of the NIR‐emitting molecules would be altered after oxidative cleavage of the specific $C_{{\mathrm{sp}}^{2}}-C_{{\mathrm{sp}}^{2}}$ bond and that the photophysical properties would change consequently.

A *N*,*N*,*O*‐type benzopyrromethene boron complex **BOBPY** was selected as a starting compound owing to its high stability, strong absorbance, high fluorescence quantum yield, and narrow emission band.^[^ ^16^ ^]^ As described in **Table** **1**, different boronic acid derivatives are substituted in the axial position, while the methyl group from 2,4‐dimethyl‐3‐ethylpyrrole renders **BOBPY** flexible for further functionalization as NIR‐emitting molecules. A short series of NIR‐emitting molecules, **NIRB1−NIRB6**, were successfully synthesized by Knoevenagel condensation reaction. All products and intermediates were fully characterized (Figures S1–S16 and S21–S28, Supporting Information). Take **NIRB1** as an example here, **BOBPY** with methylboronic acid in the axial position was condensed with 4‐hydroxybenzaldehyde to yield **NIRB1**, in which an ethylenic bond that labeled as C_a_═C_b_ was readily introduced (Table 1). In particular, the introduction of a hydroxyl group in **NIRB1** could further improve its amphiphilicity. Due to the strong electron donating ability of N,N‐dimethyl, or piperidinyl groups, **NIRB3**, **NIRB4,** and **NIRB6** show relatively low quantum yields in polar solvents such as ethanol, which are even lower in the mixture of ethanol/H_2_O (Figure S31, Supporting Information). However, **NIRB1**, **NIRB2,** and **NIRB5** showed high quantum yields even in polar solvents (> 0.30, Table 1).

**Table 1:** The photophysical properties of **NIRB1−NIRB6** and two products **P1−P2**. All properties were measured in ethanol.

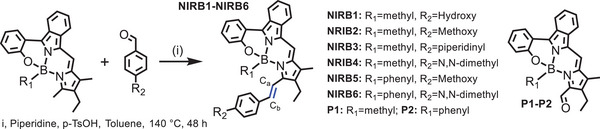				
Molecules	λ_abs_ ^a)^ [nm]	λ_em_ ^b)^ [nm]	ε_max_ ^c)^ [mol^−1^·L·cm^−1^]	Φ
**NIRB1**	689	707	84100	0.35
**NIRB2**	686	703	82100	0.31
**NIRB3**	699	740	72000	0.06
**NIRB4**	710	746	70800	0.06
**NIRB5**	691	709	70100	0.34
**NIRB6**	719	759	72700	0.03
**P1**	598	624	19200	0.55
**P2**	600	629	19800	0.48

^a)^ Wavelength of the longest absorption peak;

^b)^ Fluorescence peak, emissions of **NIRB1−NIRB6** were excited at the longest absorption wavelength and emissions of **P1−P2** were excited at 580 nm;

^c)^ Maximum molar absorbance.

### 2.2 Cu(II)/Fe(III)‐Catalyzed Oxidative Cleavage of C═C Bonds Under Mild Condition

The feasibility of the Cu(II)/Fe(III)‐catalyzed reaction was evaluated using all NIR‐emitting molecules. As one example, **NIRB1** reacted with CuCl_2_ or FeCl_3_ in ethanol at room temperature. After the reaction was completed, the product **P1** with red fluorescence was isolated. Both nuclear magnetic resonance (NMR) and high‐resolution mass spectra (HRMS) data (Figures S17,S18, and S29, Supporting Information) demonstrated that the ethylenic bond (C_a_═C_b_, Table 1) was effectively cleaved and successfully converted to aldehyde groups. On the other hand, the product **P2** from the catalytic reaction was also isolated and confirmed by NMR and HRMS data (Figures S19,S20, and S30, Supporting Information). Before transferring the reaction into living systems, all NIR‐emitting molecules were implemented to react with CuCl_2_ or FeCl_3_ in a mixture of ethanol and H_2_O (v/v = 2:1, containing 2% PEG400). Here, this mixed solvent was utilized to ensure the solubility of all NIR‐emitting molecules and avoid aggregation. As expected, **P1** and **P2** from effective cleavage of the C_a_═C_b_ bonds existing in all reaction mixtures were successfully captured by HRMS data (Figure S32, Supporting Information). Taking amphiphilicity and photophysical properties into consideration, we finally selected **NIRB1** as a representative to further investigate Cu(II)/Fe(III)‐catalyzed cleavage of C═C bonds.

**Figure 2:**
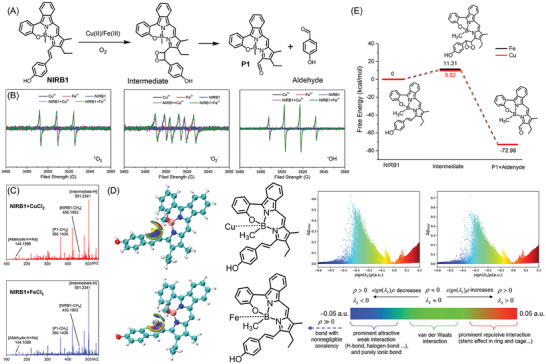
Proposed reaction mechanism between **NIRB1** and CuCl_2_/FeCl_3_. A) The proposed reaction mechanism of Cu(II)/Fe(III)‐catalyzed oxidative cleavage of C═C bonds with **NIRB1**. B) EPR spectra of **NIRB1** in the presence and absence of CuCl_2_ or FeCl_3_. C) MS data of the reaction mixture between **NIRB1** and CuCl_2_ or FeCl_3_. D) Density functional theory (DFT) calculations of interaction between **NIRB1** and CuCl_2_ or FeCl_3_ with structural optimization by using the ORCA 5.1 software package. E) The calculation of Gibbs free energies with the key molecules in the catalyzed reaction by using dispersion‐corrected basis sets.

Kinetically stable free radicals have been usually reported for various reactions catalyzed by Cu(II) and Fe(III).^[^ ^17^ ^]^ We thus proposed the plausible reaction mechanism displayed in **Figure** **2**A. As shown in the chemical structure of **NIRB1**, the coordinated boron atom is an electron deficient centre that can attract electron‐rich Cu(II)/Fe(III) with weak force to form a whole (similar to that of single‐atom catalysts).^[^ ^18^ ^]^ This further catalyzes the formation of radicals under mild conditions accompanied by normoxia (21% O_2_), like ^1^O_2_, ^•^OH, and O_2_^•−^. Therefore, electron paramagnetic resonance (EPR) was first employed to prove whether free radicals were generated in this catalytical reaction. As depicted in Figure 2B, clear EPR signals were observed after mixing CuCl_2_ or FeCl_3_ with **NIRB1** using 5,5‐dimethyl‐1‐pyrroline N‐oxide (DMPO) and 2,2,6,6‐Tetramethylpiperidine (TEMP) as the spin trap and the EPR signals were centered at 3460G–3560G. Additionally, radicals in **NIRB2−NIRB6** added with CuCl_2_ or FeCl_3_ can also be captured by EPR (Figure S33, Supporting Information). The EPR peaks indicated that the reaction involved several kinds of free radicals, such as ^1^O_2_, ^•^OH, and O_2_^•−^.^[^ ^19^ ^]^ Indeed, the generation of O_2_^•−^ and ^•^OH is mainly induced by e^−^/H^+^ transfer in the catalytical systems, while electron spin exchange results in ^1^O_2_. As is reported that ^1^O_2_ has the highest reactivity to electron‐rich acceptors of C═C bonds;^[^ ^20^ ^]^ therefore, Cu(II)/Fe(III)‐catalyzed oxidative cleavage of C═C bonds is in good agreement with ^1^O_2_‐mediated dioxetane formation/cycloreversion (Figure 2A). More importantly, the intermediate in the reaction with **NIRB1** was captured by MS data (Figure 2C), which further evidenced the proposed reaction mechanism.

To further clarify the proposed reaction mechanism, density functional theory (DFT) calculations were employed to investigate the reaction between **NIRB1** and Cu(II)/Fe(III). Herein, non‐covalent interactions in molecular systems, such as hydrogen bonds, van der Waals forces, and π–π interactions, were quantified by the reduced density gradient (RDG) method. As described in Figure 2D, van der Waals forces was speculated to govern between **NIRB1** and Cu(II)/Fe(III) by this calculation, which may promote the formation of a catalytical whole (similar to that of single‐atom catalysts) to further generate free radicals (^1^O_2_, ^•^OH and O_2_^•−^). Furthermore, the calculation of Gibbs free energies with **NIRB1**, intermediate, and **P1**, also ensured reliable quantification of the activation energy and provided insights into the thermodynamic and kinetic aspects of the reaction (Figure 2E). Consequently, these can support ^1^O_2_‐mediated dioxetane formation/cycloreversion due to the intramolecular radical addition.

### 2.3 Metal‐Triggered Cleavage of C═C for Fluorophore Activation

**Figure 3:**
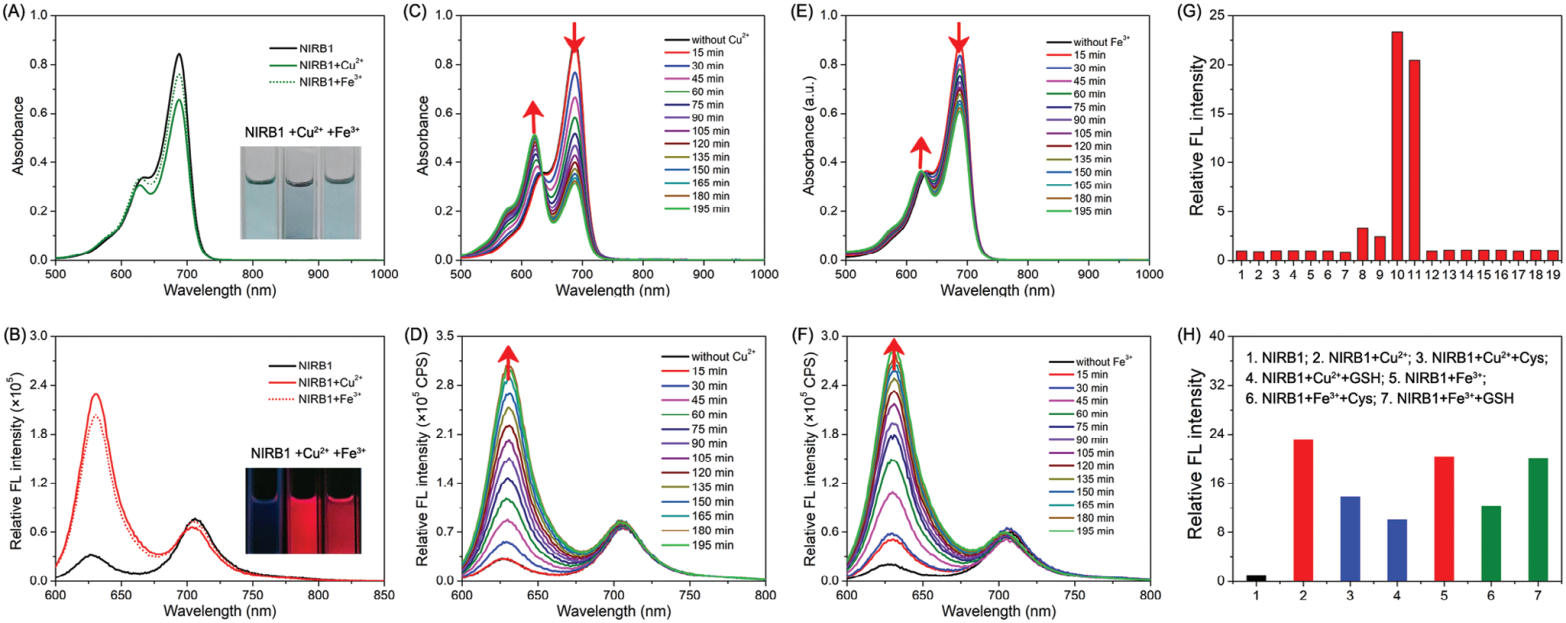
Optical properties of **NIRB1** with and without CuCl_2_ or FeCl_3_. A) Absorption spectra of **NIRB1** (10 µm) toward 50 µm CuCl_2_/FeCl_3_. B) Fluorescent spectra of **NIRB1** (10 µm) toward 50 µm CuCl_2_/FeCl_3_, excitation: 580 nm. Inserts are photoimages of **NIRB1** with and without CuCl_2_/FeCl_3_ under daylight and an ultraviolet lamp (365 nm). C–E) Time‐dependent absorption spectra of **NIRB1** (10 µm) toward 50 µm CuCl_2_ and 50 µm FeCl_3_. (D–F) Time‐dependent fluorescence spectra of **NIRB1** (10 µm) toward 50 µm CuCl_2_ and 50 µm FeCl_3_, excitation: 580 nm. G) Selectivity tests for **NIRB1** (10 µm, 1) upon the addition of different metal cations (2: Li^+^, 3: Na^+^, 4: K^+^, 5: Ca^2+^, 6: Mg^2+^, 7: Al^3+^, 8: Fe^2+^, 9: Cu^+^, 10: Cu^2+^, 11: Fe^3+^, 12: Zn^2+^, 13: Ni^2+^, 14: Mn^2+^, 15: In^3+^, 16: Co^2+^, 17: Cd^2+^, 18: Pb^2+^, 19: Hg^2+^) for 3 h. 500 µm for Na^+^, K^+^, and Ca^2+^, 50 µm for other metal ions. The emission changes were determined at 635 nm. H) Fluorescence intensity of **NIRB1** reacting with Cu(II) and Fe(III) in the presence of 1 mM cysteine (Cys) or 1 mM GSH. All tests were performed in an ethanol and H_2_O mixture (v/v = 2:1, containing 2% PEG400).

Next, we verified the hypothesis of photophysical property change. The photophysical properties of all NIR‐emitting molecules and two products were summarized in Table 1, and significant changes in photophysical properties can be observed between NIR‐emitting molecules and the corresponding products. The absorption and fluorescence responses of all NIR‐emitting molecules toward CuCl_2_ and FeCl_3_ were measured in ethanol and H_2_O mixture (v/v = 2:1, containing 2% PEG400), which displayed remarkable shifts after oxidative cleavage (Figures S34 and S35, Supporting Information). As illustrated in **Figure** **3**A,C, **NIRB1** showed a main absorption peak at 689 nm, which decreased after mixing with CuCl_2_ (50 µm), while a new absorption peak at 625 nm appeared. **NIRB1** displayed an NIR fluorescence peak at 707 nm, which shifted to 635 nm after the addition of CuCl_2_ (50 µm) (Figure 3B,D; Figure S34, Supporting Information). Similar changes in absorption and fluorescence were also observed in **NIRB1** after mixing with FeCl_3_ (50 µm) (Figure 3A,B,E,F; Figure S34, Supporting Information). More importantly, the changes in absorption and fluorescence were still observed in **NIRB1** (10 µm) after mixing with CuCl_2_ or FeCl_3_, even at very low concentrations (Figure S36, Supporting Information), which further confirmed the catalytical capacity of Cu(II)/Fe(III). To sum up, the photophysical properties of all NIR‐emitting molecules significantly changed after the reaction, but fluorescence was not quenched by paramagnetic Cu(II)/Fe(III). Therefore, the activated high‐intensity red fluorescence could be employed to monitor and track this Cu/Fe‐catalyzed reaction in living systems.

### 2.4 Executing Oxidative Cleavage of C═C bonds in Living Cells

Ideally, abiotic chemical synthesis in living systems is target‐specific and stable, while still maintaining significant activity under biological conditions. To evaluate the specificity of this catalytical reaction in living systems, different bio‐related metal cations were treated with **NIRB1**. As monitored by fluorescence intensity (**NIRB1** at 635 nm, Figure 3G), the oxidative cleavage of the C_a_═C_b_ bonds in **NIRB1** was specifically catalyzed by Cu(II) and Fe(III), while other biologically relevant metal ions showed negligible catalytic effect. **NIRB1** also maintained good stability in the presence of ROS/RNS (H_2_O_2_, ClO^−^, and ONOO^−^) and biothiols (glutathione and cysteine) (Figure S37, Supporting Information) across a pH range of 4.4−7.4 (Figures S38–S41, Supporting Information). Importantly, the Cu(II)/Fe(III)‐catalyzed reactions are still very efficient in the presence of bio‐related reductants (1 mM glutathione or cysteine) (Figure 3H).

Subsequently, an MTT (3‐(4,5‐dimethyl‐2‐thiazolyl)‐2,5‐diphenyl‐2‐H‐tetrazolium bromide) study of **NIRB1** was performed in human glioma U87 cell lines, which showed low cytotoxicity at given concentrations from 5 to 50 µm (Figure S42, Supporting Information). Indeed, Cu(I) and Fe(II) were considered to be mainly found in biological systems,^[^ ^21^ ^]^ exogenous Cu(II) and Fe(III) were thus introduced. Cells treated with both CuCl_2_ and **NIRB1** presented obvious red fluorescence due to the bond cleavage reaction (Figure S43A, Supporting Information). Significantly enhanced red fluorescence was also observed in cells treated with both FeCl_3_ and **NIRB1** (Figure S43B, Supporting Information). The relative proportion of red emitted products (**P1**) has been ulteriorly detected by HPLC in the reaction conducted in PBS buffer and cells, which is shown in Table S1 (Supporting Information). Significantly, red emitted **P1** was increased in the reaction conducted in PBS buffer at the indicated concentration of CuCl_2_/FeCl_3_, as well as in the cell lysis. These results demonstrated that the increase in red fluorescence is correlated with the formation of **P1**. Furthermore, MS data confirmed the product **P1** in the extraction of U87 cells after coincubation (Figure S44, Supporting Information). Turn‐on red fluorescence and MS data clearly revealed that the cleavage of C═C bonds could well work in living cells. In addition, co‐localization experiments with MitoTracker Green FM (λ_ex_ = 490 nm and λ_em_ = 513 nm) indicated acceptable targeting ability of **NIRB1** and red‐emitted product to mitochondria with good Pearson's co‐localization coefficients (Figures S45–S48, Supporting Information). Current results indicated to a certain extent that Cu(II)/Fe(III)‐catalyzed reaction is related to mitochondria.

**Figure 4:**
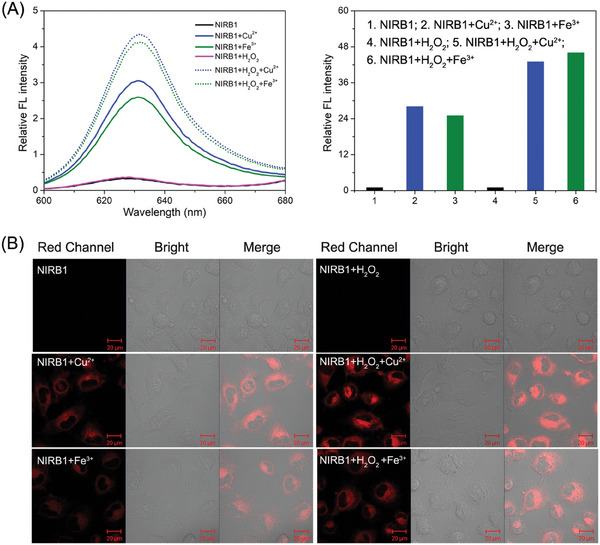
Abiotic bond cleavage reaction of **NIRB1** in living cells. A) Fluorescent spectra of **NIRB1** (10 µm) toward 50 µm CuCl_2_ and 50 µm FeCl_3_ promoted by H_2_O_2_ (50 µm). B) Confocal images of exogenous Cu(II)/Fe(III)‐catalyzed abiotic bond cleavage of **NIRB1** (10 µm) in U87 cells, CuCl_2_ (50 µm), FeCl_3_ (50 µm), H_2_O_2_ (50 µm), scale bar: 20 µm.

Notably, the fluorescence response of **NIRB1** at 635 nm in the presence of both H_2_O_2_ and Cu(II) or Fe(III) was considerably stronger than that of **NIRB1** titrated with only Cu(II) or Fe(III) (**Figure** **4**A), which displayed more efficient cleavage of the C_a_ ═ C_b_ bonds under oxidative condition. We further investigated the response of **NIRB1** toward Cu(II) and Fe(III) in living U87 cells under oxidative stress. U87 cells were pre‐treated with exogenous H_2_O_2_ and then incubated with **NIRB1** and CuCl_2_, respectively. As shown in Figure 4B, the presence of H_2_O_2_ indeed improved catalytic reaction with obviously enhanced red fluorescence compared with that of cells just treated with **NIRB1** and CuCl_2_. A similar enhancement was also presented in H_2_O_2_‐treated cells with **NIRB1** and FeCl_3_. The results of cell imaging indicated that H_2_O_2_ is a promoter of Cu(II)/Fe(III)‐catalyzed oxidative cleavage of the C_a_═C_b_ bonds, which agrees well with previous reports.^[^ ^22^ ^]^

### 2.5 Oxidative Cleavage of C═C Bonds by Food‐Derived Cu(II)/Fe(III) in Living Zebrafish

**Figure 5:**
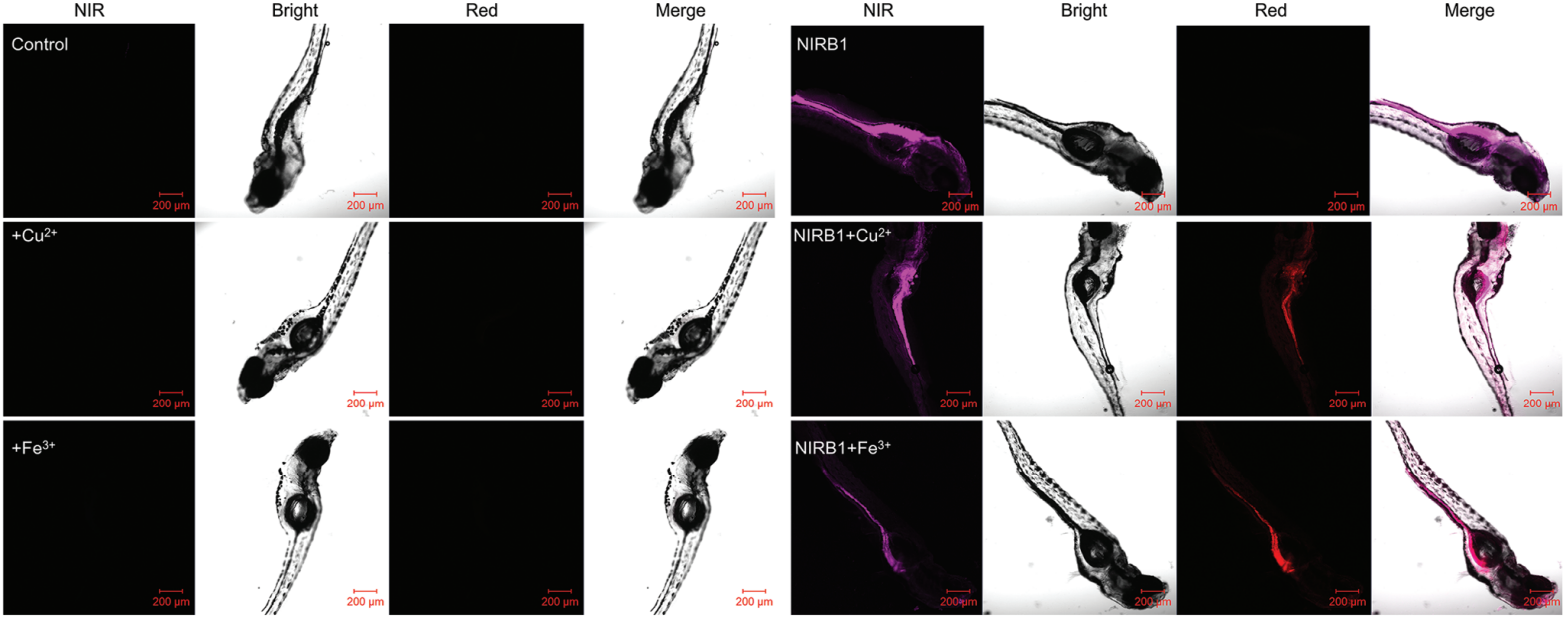
Abiotic bond cleavage reaction of **NIRB1** in zebrafish. Confocal images of food‐derived Cu(II)/Fe(III)‐catalyzed abiotic bond cleavage of **NIRB1** (10 µm) in zebrafish, CuCl_2_ (50 µm) and FeCl_3_ (50 µm), scale bar: 200 µm.

Food‐derived metal accumulation is an important safety issue for seafood, thus the feasibility of Cu(II)/Fe(III)‐catalyzed abiotic bond cleavage was further identified in zebrafish and is significant to metal detection in seafood. As described in **Figure** **5**, after treatment with **NIRB1**, the obvious NIR fluorescence could be observed which indicated the uptake of **NIRB1** in living zebrafish. However, compared with zebrafish treated with sole **NIRB1**, those treated with both CuCl_2_ and **NIRB1** exhibited strong red fluorescence, which demonstrated the oxidative cleavage of C═C bonds in living zebrafish. Similar results were also displayed in the zebrafish treated with both FeCl_3_ and **NIRB1** (Figure S49, Supporting Information). Collectively, this further demonstrated Cu(II)/Fe(III)‐catalyzed oxidative cleavage of C═C bonds can be successfully realized in living zebrafish.

### 2.6 Cu(II)/Fe(III)‐Catalyzed Cleavage of C═C Bonds in AD‐Afflicted Mouse Brains

**Figure 6:**
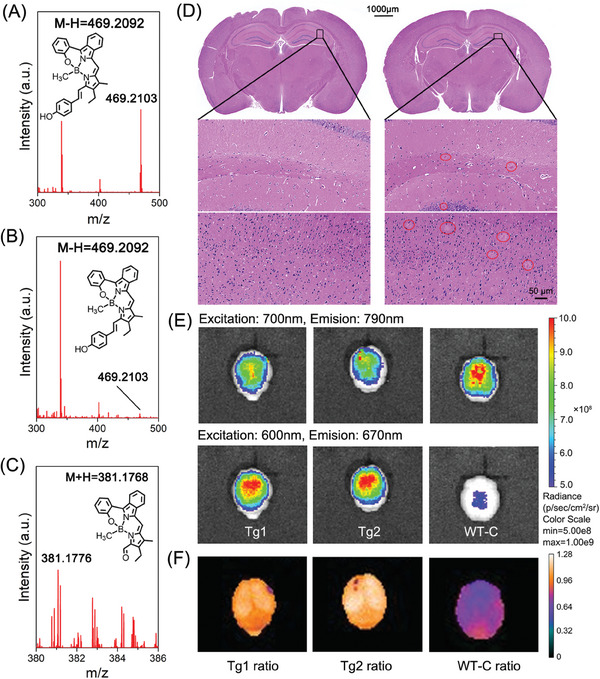
Abiotic bond cleavage reaction of **NIRB1** in mouse brain. MS spectra of the extraction of brain homogenate from normal mice after intravenous injection of **NIRB1** for 3 h (A) and 6 h (B), and from Tg mice after intravenous injection of **NIRB1** for 5 h (C). D) Histological staining of the brain slices in the hippocampus region from normal (left) and AD (right) mouse using H&E. The lesion parts were marked with red circles. E) Fluorescent imaging in ex vivo brains from Tg and WT mice with **NIRB1** after intravenous injection for 5 h. F) The ratio of fluorescence intensity from 670 nm and 790 nm in different brain tissues. WT‐C: WT mouse injected with **NIRB1**.

As well known, abnormal copper/iron is recognized as an important pathological feature of AD, thus this Cu(II)/Fe(III)‐catalyzed cleavage of C═C bonds was proposed to apply in AD‐afflicted mouse brains. Prior to applying this abiotic bond cleavage reaction in a mouse brain, the biostability of **NIRB1** was first evaluated. There presented no variations in photophysical properties in mouse serum, indicating favorable biostability (Figure S50, Supporting Information). Hemolytic analysis showed very low hemolysis percentages (≈ 0.5%) of erythrocytes even at 20 µg mL^−1^, displaying adequate hemocompatibility (Figure S51, Supporting Information). Furthermore, the ability of **NIRB1** to penetrate the blood‐brain barrier (BBB) in mouse brains was confirmed by MS data. After intravenous injection of **NIRB1** (1.2 mg kg^−1^) for 3 and 6 h, an extraction of brain homogenate was analyzed by mass spectrometry. As shown in **Figure** **6**A,B, **NIRB1** was successfully captured in the extraction, providing strong evidence that **NIRB1** could successfully cross the BBB.

Subsequently, abiotic cleavage of the C_a_═C_b_ bonds in **NIRB1** was performed in transgenic mice (APP/PS1, Tg) and age‐matched wild‐type (WT) mice. Firstly, histological staining of the brain slices by H&E and Thioflavine‐S confirmed Tg mice as AD‐afflicted mice (Figure 6D; Figure S52, Supporting Information). Fluorescent imaging was utilized to monitor the catalytical cleavage of the C_a_═C_b_ bonds in mouse brains, which was repeated with the confirmed Tg mice, and the results were reproducible. After injection for 5 h, mouse brains were excised after the perfusion of sterile saline solution, to evaluate the effective reaction in mouse brains by fluorescence imaging, excluding interferences from the skin, skull, and blood. Compared with the brain tissue from WT‐C mice treated with **NIRB1**, the fluorescent signals at 670 nm in Tg mouse brain tissues were stronger, which was attributed to the activation of red fluorophores after C═C bonds cleavage. Meanwhile, the fluorescent signals at 790 nm in Tg mouse brain tissues were weaker than those of normal ones (Figure 6E), which was explained by the reactive consumption of **NIRB1**. Furthermore, the ratio of fluorescence intensity from 670 and 790 nm in Tg mouse brain tissues is higher than that of normal ones (Figure 6F), which revealed a better cleavage of **NIRB1** in AD‐afflicted mouse brains. More importantly, the efficient reaction in Tg mice was further supported by MS data. The product of **NIRB1** after reaction was successfully extracted from the brain homogenate of Tg mice and was confirmed by MS data (Figure 6C).

Currently, there have been no effective fluorescent probes for imaging Cu(II) or Fe(III) in living AD‐afflicted mouse brains so far. One of the difficulties is that, in conventional coordination‐based detection, the paramagnetic Cu(II) and Fe(III) can quench the fluorescence of chelators. Differently, the Cu(II)/Fe(III)‐catalyzed oxidative cleavage of C═C bonds in this work can effectively activate red fluorescence also does not consume Cu(II)/Fe(III), which is useful and valuable for imaging‐guided explorations of brain diseases. Although there are still some issues of fluorescent imaging in vivo in AD‐afflicted mouse brain to be addressed in current or further study, this metal‐catalyzed strategy may encourage the continuous development of analyte‐inconsumable fluorescent probes for imaging in vivo and raise many interests in catalysis‐based diagnosis and therapy.

## 3 Conclusion

In summary, we have achieved Cu(II)/Fe(III)‐catalyzed oxidative cleavage of C═C bonds in living systems, which is anticipated to further inspire the exploration of Cu/Fe related diseases with a breakthrough. There is no doubt that the catalytic reaction here does not disturb native physiological processes much. Importantly, the strong activated red fluorescence is not quenched by paramagnetic Cu(II)/Fe(III). Both advantages above are highly desirable for studying biological processes within sophisticated living systems. We envision that the successful abiotic cleavage of C═C bonds may bring a new opportunity to develop non‐invasive and cost‐effective diagnostic and therapeutic measures for diseases in the future.

## 4 Experimental Section

### Synthesis of BOBPY1 and BOBPY2

Compound **1**, compound **2**, **BOBPY1,** and **BOBPY2** were synthesized by referring to previous studies.^[^ ^16^, ^23^ ^]^ POCl_3_ (5.2 mmol, 0.797 g) was added to a dichloromethane solution (15 mL) of 2,4‐dimethyl‐3‐ethylpyrrole (10.4 mmol, 1.281 g) at 0 °C. Then a solution of compound 2 (5.2 mmol, 1.233 g) in dichloromethane (25 mL) was added dropwise to the reaction mixture at 0 °C. The mixture was then passed through a pad of silica gel, concentrated, and dissolved in ethyl acetate (50 mL). To this solution, methylboronic acid (52 mmol, 3.112 g) was added and the reaction mixture was refluxed for 3 h. After evaporation of the solvent, the residual product was purified by silica gel column chromatography (dichloromethane/n‐hexane = 1/1) to obtain **BOBPY1** (yield 54.36%). ^1^H NMR (400 MHz, CDCl_3_) δ = 8.16 (d, J = 8.2, 1H), 8.05 (dd, J = 7.8, 1.6, 1H), 7.86 (d, J = 8.0, 1H), 7.46 (dd, J = 11.1, 3.9, 1H), 7.40‐7.33 (m, 2H), 7.30 (s, 1H), 7.10 (dd, J = 8.3, 0.9, 1H), 7.00 (dd, J = 10.9, 4.2, 1H), 2.61 (s, 3H), 2.45 (q, J = 7.6, 2H), 2.25 (s, 3H), 1.09 (t, J = 7.6, 3H), ‐0.06 (s, 3H). ^13^C NMR (101 MHz, CDCl_3_) δ = 157.07, 150.15, 142.78, 135.23, 132.66, 132.62, 131.60, 130.00, 128.68, 128.17, 126.05, 125.21, 125.03, 123.31, 120.40, 119.54, 119.38, 119.12, 115.83, 41.01, 17.53, 14.89, 12.80, 9.48. HRMS (ESI^+^) m/z: calcd for C_24_H_23_BN_2_O [M+H]^+^, 367.1976; found, 367.2099.

POCl_3_ (5 mmol, 0.776 g) was added to a dichloromethane solution (15 mL) of 2,4‐dimethyl‐3‐ethylpyrrole (10 mmol, 1.232 g) at 0 °C. Then a solution of compound 2 (5 mmol, 1.186 g) in dichloromethane (25 mL) was added dropwise to the reaction mixture at 0 °C. The reaction mixture was stirred at room temperature for 4 h. To this solution, phenylboronic acid (50 mmol, 6.096 g) was dissolved in dichloromethane and added. Then the reaction mixture was stirred for another 4 h. After evaporation of the solvent, the residual product was purified by silica gel column chromatography (dichloromethane/n‐hexane = 1/2) to give **BOBPY2** (yield 59.78%). ^1^H NMR (400 MHz, CDCl_3_) δ = 8.11 (d, J = 8.2, 1H), 7.96 (dd, J = 7.9, 1.5, 1H), 7.89 (d, J = 8.1, 1H), 7.50–7.44 (m, 1H), 7.44–7.38 (m, 2H), 7.36–7.31 (m, 1H), 7.28 (dd, J = 8.3, 1.1, 1H), 7.19 (dd, J = 6.5, 3.1, 2H), 7.06–7.00 (m, 3H), 6.99–6.93 (m, 1H), 2.47 (s, 3H), 2.38 (qd, J = 7.5, 2.1, 2H), 2.27 (s, 3H), 1.04 (t, J = 7.6, 3H). ^13^C NMR (101 MHz, CDCl_3_) δ = 157.08, 150.98, 143.27, 135.34, 133.29, 133.03, 132.10, 131.55, 130.36, 128.76, 128.46, 127.10, 126.42, 126.36, 125.72, 125.38, 123.43, 120.15, 119.67, 119.58, 119.09, 115.98, 17.50, 14.86, 13.08, 9.60. HRMS (ESI^+^) m/z: calcd for C_29_H_25_BN_2_O [M+H]^+^, 429.2133; found, 429.2251.

### General Synthesis of NIRB1‐NIRB6

As depicted in Scheme S1 (Supporting Information), the synthesis of **NIRB1−NIRB6** was consulting previous studies by modification.^[^ ^23^, ^24^ ^]^ To compound **BOBPY1** (1 mmol) or **BOBPY2** (1 mmol), and different benzaldehyde derivatives (1.2 mmol) in 100 ml toluene added piperidine (4 ml) and *p*‐TsOH (100 mg) through a syringe. The solution was refluxed at 140 °C for 48 h in a round‐bottomed flask and any water formed during the reaction was removed by using a Soxhlet extractor containing anhydrous CaCl_2_. After the reaction finished, the solvent was removed under vacuum. The crude product was purified from a chromatograph (silica gel, dichloromethane/n‐hexane = 1/1), giving **NIRB1−NIRB6**.

### NIRB1

Yield 35.21%. ^1^H NMR (400 MHz, DMSO) δ = 9.80 (s, 1H), 8.38 (d, J = 8.3, 1H), 8.26 (dd, J = 7.9, 1.4, 1H), 8.20 (d, J = 8.0, 1H), 7.94 (s, 1H), 7.78 (d, J = 16.8, 1H), 7.61 (t, J = 7.6, 1H), 7.54–7.43 (m, 4H), 7.28 (d, J = 16.8, 1H), 7.14 (dd, J = 8.2, 0.9, 1H), 7.11–7.05 (m, 1H), 6.89 (d, J = 8.6, 2H), 2.82–2.64 (m, 2H), 2.29 (s, 3H), 1.19 (t, J = 7.5, 3H), ‐0.15 (s, 3H). ^13^C NMR (101 MHz, DMSO) δ = 158.61, 156.90, 147.18, 142.90, 134.99, 134.35, 133.91, 133.38, 133.26, 130.82, 129.42, 129.10, 128.61, 128.46, 127.19, 126.70, 126.61, 124.03, 121.20, 120.62, 120.43, 118.79, 117.51, 116.80, 116.55, 18.75, 14.58, 9.35. HRMS (ESI^+^) m/z: calcd for C_31_H_27_BN_2_O_2_ [M], 470.2166; found, 470.2222. [M‐CH_3_]^+^ 455.1931; found, 455.1976.

### NIRB2

Yield 24.59%. ^1^H NMR (400 MHz, CDCl_3_) δ = 8.18 (d, J = 6.8, 1H), 7.98 (d, J = 63.3, 3H), 7.69–7.28 (m, 6H), 7.23–6.94 (m, 5H), 3.88 (s, 3H), 2.76 (s, 2H), 2.28 (s, 3H), 1.26 (d, J = 7.5, 3H), 0.02 (s, 3H). ^13^C NMR (101 MHz, CDCl_3_) δ = 159.69, 157.43, 147.10, 143.66, 135.05, 133.13, 132.96, 132.22, 131.17, 130.89, 129.11, 128.44, 128.06, 126.68, 126.22, 125.69, 123.49, 120.54, 119.89, 119.58, 119.16, 119.08, 114.59, 114.37, 55.43, 18.90, 14.23, 9.20. HRMS (ESI^+^) m/z: calcd for C_32_H_29_BN_2_O_2_ [M+H]^+^, 485.2395; found, 485.2463.

### NIRB3

Yield 21.74%. ^1^H NMR (400 MHz, CDCl_3_) δ = 8.17 (d, J = 7.6, 2H), 7.97 (d, J = 68.7, 3H), 7.62–7.27 (m, 6H), 7.26–6.84 (m, 5H), 3.29 (s, 3H), 2.27 (s, 2H), 2.27 (s, 3H), 1.66 (d, J = 20.1, 4H), 1.56 (s, 2H), 1.25 (t, J = 7.5, 3H), 0.02 (s, 3H). ^13^C NMR (101 MHz, CDCl_3_) δ = 157.36, 134.87, 133.34, 133.05, 132.68, 131.00, 128.95, 128.18, 127.94, 126.09, 125.49, 123.38, 120.52, 119.83, 119.50, 119.22, 117.84, 115.93, 114.26, 49.98, 25.60, 24.34, 18.95, 14.19, 9.20. HRMS (ESI^+^) m/z: calcd for C_36_H_36_BN_3_O [M+H]^+^, 538.2985; found, 538.3074.

### NIRB4

Yield 35.08%. ^1^H NMR (400 MHz, CDCl_3_) δ = 8.17 (d, J = 8.2, 1H), 8.05 (dd, J = 7.8, 1.4, 1H), 7.89 (dd, J = 12.3, 9.7, 2H), 7.56 (d, J = 8.8, 2H), 7.47 (s, 1H), 7.36 (s, 3H), 7.25 (s, 1H), 7.21‐7.16 (m, 1H), 7.01 (s, 1H), 6.79 (d, J = 8.8, 2H), 3.04 (s, 6H), 2.77 (qq, J = 14.8, 7.5, 2H), 2.27 (s, 3H), 1.27 (t, J = 7.5, 3H), 0.01 (s, 3H). ^13^C NMR (101 MHz, CDCl_3_) δ = 157.28, 150.49, 148.47, 142.42, 134.75, 133.60, 133.51, 133.38, 132.48, 130.98, 128.83, 128.14, 128.01, 126.61, 126.10, 126.00, 125.34, 123.31, 120.49, 119.78, 119.45, 119.33, 116.84, 114.09, 112.49, 41.01, 40.44, 18.97, 14.17, 9.19. HRMS (ESI^+^) m/z: calcd for C_33_H_32_BN_3_O [M+H]^+^, 498.2711; found, 498.2786.

### NIRB5

Yield 30.23%. ^1^H NMR (400 MHz, CDCl_3_) δ = 8.13 (d, J = 8.1, 1H), 8.04–7.81 (m, 3H), 7.62‐7.27 (m, 8H), 7.25–7.21 (m, 2H), 7.04–6.93 (m, 6H), 3.88 (s, 3H), 2.69 (s, 2H), 2.29 (s, 3H), 1.20 (t, J = 7.5, 3H). ^13^C NMR (101 MHz, CDCl_3_) δ = 159.70, 157.46, 147.76, 143.96, 135.14, 133.78, 133.51, 133.28, 132.58, 131.33, 131.17, 131.09, 129.10, 128.69, 128.08, 127.25, 127.15, 126.52, 126.46, 125.84, 123.62, 120.31, 119.93, 119.86, 119.35, 119.04, 114.75, 114.36, 55.41, 53.44, 18.89, 14.13, 9.29. HRMS (ESI^+^) m/z: calcd for C_37_H_31_BN_2_O_2_ [M+H]^+^, 547.2551; found, 547.2638.

### NIRB6

Yield 23.18%. ^1^H NMR (400 MHz, DMSO) δ = 8.34 (d, J = 8.3, 1H), 8.25 (d, J = 8.1, 1H), 8.16 (d, J = 7.6, 1H), 8.05 (s, 1H), 7.84 (d, J = 16.8, 1H), 7.60 (t, J = 7.5, 1H), 7.52 (d, J = 8.8, 3H), 7.45 (t, J = 7.7, 1H), 7.31 (d, J = 7.6, 1H), 7.16 (d, J = 16.8, 1H), 7.11–7.00 (m, 3H), 6.97–6.90 (m, 3H), 6.86 (d, J = 8.6, 2H), 3.01 (s, 6H), 2.68 (dt, J = 22.4, 7.5, 2H), 2.30 (s, 3H), 1.14 (t, J = 7.5, 3H). ^13^C NMR (101 MHz, DMSO) δ = 156.81, 151.11, 148.76, 141.93, 135.45, 134.76, 134.34, 134.03, 133.79, 131.17, 131.08, 129.31, 128.46, 128.16, 127.59, 127.08, 126.88, 126.78, 126.61, 125.60, 123.97, 121.22, 120.88, 120.29, 119.08, 116.49, 116.06, 112.97, 55.38, 49.07, 14.33, 9.39. HRMS (ESI^+^) m/z: calcd for C_38_H_34_BN_3_O [M+H]^+^, 560.2868; found, 560.2850.

### Reaction of NIR‐Emitting Molecules with CuCl2/FeCl3

Here **NIRB1** and **NIRB6** were taken as an example. **NIRB1**/**NIRB6** (0.2 mmol) was dissolved in 10 mL ethanol, and CuCl_2_/FeCl_3_ (1 mmol) also dissolved in 10 mL ethanol was added dropwise. Then the mixture was stirred at room temperature for 3 h. After the reaction was completed, the solvent was removed in vacuo, the crude product was purified by a silica gel column to get the red‐emitting product.

### P1

Yield 20.15%, ^1^H NMR (400 MHz, CDCl_3_) δ = 10.58 (s, 1H), 8.23 (d, J = 8.0, 1H), 8.11 (dd, J = 7.9, 1.5, 1H), 7.97 (d, J = 7.9, 1H), 7.66 (t, J = 7.5, 1H), 7.58‐7.49 (m, 2H), 7.33 (s, 1H), 7.13 (d, J = 8.4, 1H), 7.04 (t, J = 7.6, 1H), 2.93 (dt, J = 15.0, 7.5, 1H), 2.78 (dq, J = 14.9, 7.5, 1H), 2.25 (s, 3H), 1.16 (t, J = 7.5, 3H), 0.10 (s, 3H). ^13^C NMR (101 MHz, CDCl_3_) δ = 185.96, 136.08, 130.89, 128.14, 127.41, 124.71, 121.26, 120.90, 120.06, 115.14, 18.32, 14.11, 8.63. HRMS (ESI^+^) m/z: calcd for C_24_H_21_BN_2_O_2_ [M+H]^+^, 381.1774; found, 381.1806, [M‐CH_3_]^+^, 365.1461; found, 365.1478.

### P2

Yield 24.87%, ^1^H NMR (400 MHz, CDCl_3_) δ = 10.49 (s, 1H), 8.17 (d, J = 8.0, 1H), 8.03 (dd, J = 8.0, 1.4, 1H), 7.99 (d, J = 7.9, 1H), 7.65 (t, J = 7.6, 1H), 7.55 (ddd, J = 19.4, 11.8, 4.4, 2H), 7.46 (s, 1H), 7.31 (d, J = 8.3, 1H), 7.14 (dd, J = 6.5, 3.0, 2H), 7.06‐6.99 (m, 4H), 2.90 (dd, J = 13.3, 7.5, 1H), 2.69 (dd, J = 13.3, 7.4, 1H), 2.28 (s, 3H), 1.11 (t, J = 7.5, 3H). ^13^C NMR (101 MHz, CDCl_3_) δ = 185.88, 159.67, 152.23, 139.42, 136.57, 136.35, 136.17, 133.87, 132.59, 131.18, 131.03, 130.93, 129.12, 128.27, 127.81, 127.51, 127.10, 124.86, 120.91, 120.38, 117.63, 115.30, 18.31, 14.49, 8.76. HRMS (ESI^+^) m/z: calcd for C_29_H_24_BN_2_O_2_ [M+H]^+^, 443.1925, found, 443.1945; [M‐Phe]^+^, 365.1461, found, 365.1478.

### Cell Viability Detected by MTT

Human glioma cell lines (U87) were cultured in a complete DMEM culture medium, containing 10% fetal bovine serum (FBS) and 1% penicillin‐streptomycin at 37 ^°^C in the atmosphere containing 5% CO_2_. After 90% confluence, the cells were cultured into 96‐well plates (100 µL, 5000 cells/well). Cells were treated with different concentrations of **NIRB1** (5, 10, 20, 30, 40, 50 µm) for 24 h. Then, MTT stock solution (5.0 mg mL^−1^, 100 µL) was added to each well for incubating 4 h to form purple crystal formazan. At the end of incubation, DMSO (100 µL) was added for 10 min microvibration after removing the medium. The absorbance was measured at 570 nm on a microplate reader (Thermo, USA).

### Density Functional Theory (DFT) Calculations

All calculations were performed using the ORCA 5.1 software package.^[^ ^25^ ^]^ The hybrid functional B3LYP (Becke, 3‐parameter, Lee‐Yang‐Parr),^[^ ^26^ ^]^ combined with the def2‐TZVP (triple‐zeta valence polarization) basis set,^[^ ^27^ ^]^ was employed to optimize the molecular structures and compute single‐point energies. To account for dispersion interactions, Grimme's D3 dispersion correction with Becke–Johnson damping (DFT‐D3(BJ))^[^ ^28^ ^]^ was further applied. Besides, the structural optimization process included a global optimization algorithm to refine the positions of metal ions in the molecular system. This approach ensured accurate localization of the metal centers, which were critical to the chemical reactivity and stability of the system. Following structural optimization, single‐point energy calculations were performed to achieve higher accuracy in energy evaluation. To analyze weak intermolecular interactions, the reduced density gradient (RDG) method^[^ ^29^ ^]^ was employed. RDG was a widely used tool for visualizing and quantifying non‐covalent interactions in molecular systems, such as hydrogen bonds, van der Waals forces, and π‐π interactions. These interactions were mapped to provide a detailed understanding of the non‐covalent forces governing the reaction pathway. Additionally, vibrational frequency calculations were performed using the same level of theory B3LYP/def2‐TZVP with DFT‐D3(BJ) to verify the nature of the stationary points (minimum energy structures and transition states). The vibrational frequencies were also utilized to compute zero‐point energy (ZPE) corrections, thermal corrections, and entropy contributions. These corrections were included in the calculation of Gibbs free energies to obtain the corrected free energy profiles of the reaction. Furthermore, the reaction energy barriers were calculated using dispersion‐corrected basis sets to achieve a high level of accuracy in describing the electronic and geometric properties of the system.

### Cell Treatment and Catalytic Reaction by Exogenous Cu(II)/Fe(III) in Living Cells

Human glioma U87 cell lines were cultured in a complete DMEM medium containing 10% fetal bovine serum (FBS) and 1% penicillin‐streptomycin at 37 ^°^C in 5% CO_2_. After 90% confluence, cells were seeded in Petri dishes for 24 h. CuCl_2_ and FeCl_3_ dissolved in PBS buffer solution were used as exogenous Cu(II) and Fe(III) sources. Cells were randomly divided into 6 groups as follows:

1. Control group (10 µm **NIRB1**, 3 h),
2. H_2_O_2_ group (50 µm H_2_O_2_, 30 min; then 10 µm **NIRB1**, 3 h),
3. Cu(II) group (50 µm CuCl_2_, 30 min; then 10 µm **NIRB1**, 3 h),
4. Cu(II)‐H_2_O_2_ group (50 µm H_2_O_2_, 30 min; then 50 µm CuCl_2_, 30 min; then 10 µm **NIRB1**, 3 h),
5. Fe(III) group (50 µm FeCl_3_, 30 min; then 10 µm **NIRB1**, 3 h),
6. Fe(III)‐H_2_O_2_ group (50 µm H_2_O_2_, 30 min; then 50 µm FeCl_3_, 30 min; then 10 µm **NIRB1**, 3 h),

H_2_O_2_ (50 µm) was pretreated to stimulate oxidative stress. Finally, the residual medium was removed by washing three times with PBS buffer solution (pH = 7.4). Images were collected by a confocal fluorescence microscope (Carl Zeiss LSM710, Germany).

### Cell Lysis and Extraction

U87 cells were cultured in a complete DMEM medium containing 10% FBS and 1% penicillin‐streptomycin at 37 ^°^C in 5% CO_2_. After 90% confluence, cells were seeded in a culture flask for 24 h. Then cells were respectively treated with CuCl_2_ (100 µm) and the NIR‐emitting molecules (20 µm **NIRB1**) for 4 h. Medium was discarded before washing with PBS three times. After adding RIPA lysis buffer, 5 mL PBS and 5 mL methanol were added to the mixture, then stirred and centrifuged. The supernatant was extracted by ethyl acetate and dichloromethane, the organic phase was collected and concentrated. The extractions were analyzed by mass spectrometry (Q‐exactive, Thermofisher, USA).

### Abiotic Bond Cleavage Reaction by Food‐Derived Cu(II)/Fe(III) in Zebrafish

Zebrafish were acclimated for 4 days at 28 ^°^C in a 14 h light/10 h dark circular environment. The experiment was conducted in an E3 embryo medium. After the addition of CuCl_2_ or FeCl_3_ (50 µm), the zebrafish were incubated for 2 h for acclimatization, followed by replacing the media with **NIRB1** (10 µm) and then incubating again for 2 h. After washing thrice with the E3 embryo medium, the images of zebrafish were captured under a fluorescence microscope (LSM710, Carl Zeiss, Germany).

### Animals

All animal procedures were approved by the Institutional Animal Care and Use Committee of the Southern University of Science and Technology (approval number: SUSTC‐JY2019164) in accordance with published National Institutes of Health guidelines. Transgenic C57BL/6 mice (APP/PS1, female, 19 months, 18–22 g) and age‐matched normal C57BL/6 mice (female, 18–22 g, wild‐type) were purchased from the Shanghai Research Center of the Southern Model Organisms. All animals were free access to food and water and housed under sterile conditions at room temperature with a 12 h light/dark cycle.

### Hemolysis Assay

Hemolysis assay was detected with fresh C57BL/6 mice erythrocytes. The erythrocytes were collected via centrifugation of blood at 1500 rpm for 3 min and washed three times with PBS (pH = 7.4). A stock solution (2 mg/mL) of **NIRB1** was prepared in DMSO. The stock dispersion was prepared by mixing 1 mL of centrifuged erythrocytes into 9 mL of PBS (pH = 7.4) and the final hematocrit level of red blood cells (RBC) was ≈ 10%. Then NIRB1 was dispersed at different concentrations (4, 10, and 20 µg mL^−1^). The mixture was incubated for 3 h at 37 °C. The percentage of hemolysis was measured by UV‐vis analysis of the supernatant at 540 nm absorbance after centrifugation at 12 000 rpm for 15 min. Saline was the negative control, and pure water was the positive control. The percentage of hemolysis was calculated using the following formula:

(1) $$\mathrm{hemolysis}\;\left(\%\right)=\left(A_{S}-A_{N}\right)/\left(A_{P}-A_{N}\right)\times 100\%$$

where A_S_ is the absorbance resulting from the addition of **NIRB1** to the erythrocyte suspension, A_N_ is the absorbance following the addition of saline as a negative control, and A_P_ is the absorbance following the addition of deionized water as a positive control.

### Blood‐Brain Barrier (BBB) Penetrating Test

A solution of **NIRB1** (1.2 mg kg^−1^) in sterile saline solution was injected via the tail vein of C57BL/6 mice (wild type). After intravenous injection for 3 h and 6 h, mice were anesthetized and perfused with sterile saline solution to remove blood in the brain. Then, brain samples were removed and homogenized with 1 mL saline solution. The homogenate was processed by methanol, acetonitrile, and ethyl acetate, and the organic phase was collected and concentrated. These samples were analyzed by mass spectrometry (Q‐exactive, Thermofisher, USA).

### Abiotic Bond Cleavage Reaction in AD‐Afflicted Mouse Brain

Fluorescent imaging was performed on an IVIS Spectrum Imaging System (PerkinElmer, PE) as proof of the oxidative cleavage of C_a_═C_b_ bonds. Here, APP/PS1 transgenic C57BL/6 mice (n = 3, female, 19 months) and age‐matched normal C57BL/6 mice (n = 3, female, wild‐type) were used. Three Tg mice and three WT mice were intravenously injected with **NIRB1** (1.2 mg kg^−1^, 200 µL) in a sterile saline solution. After injection for 5 h, all mice were perfused with sterile saline solution and then mouse brains were excised. Fluorescence signals from the brain tissues were recorded. Imaging data were analyzed by systematic software. The ratio of fluorescence intensity from 670 and 790 nm was analyzed by MATLAB and ImageJ.

### Statistical Analysis

All experimental data were processed with the Origin 2018 software. Structural representations of NIR‐emitting molecules were drawn in ChemDraw software. Plots were arranged in PowerPoint and then further modified by Adobe Photoshop. Fluorescent images were processed by ImageJ and Figure 1C was made by Biorender with permission.

## Conflict of Interest

The authors declare no conflict of interest.

## Author Contributions

C.W. conceived and performed experiments, and wrote the paper. C.W. and J.T. performed the synthesis and the data analysis. D.C. and C.W. performed the mice experiments and data analysis. Z.W. performed the EPR experiments and data analysis. C.W., and X.Z. conceived and supervised the research, revised the manuscript, and acquired funding. All authors contributed to and reviewed the manuscript preparation.

## Supporting information



Supporting Information

## Data Availability

The data that support the findings of this study are available in the supplementary material of this article.
