# Supplementary material for: Metal‐Catalyzed Abiotic Cleavage of C═C Bonds for Effective Fluorescence Imaging of Cu(II) and Fe(III) in Living Systems
Source: Adv Sci (Weinh). 2025 Jan 9;12(8):2412407. doi: 10.1002/advs.202412407 (PMC11848571; doi:10.1002/advs.202412407)
Supplement: Supplementary file 1 — Supporting Information [file ADVS-12-2412407-s001.docx]

Supporting Information

Metal-Catalyzed Abiotic Cleavage of C=C Bonds for Effective Fluorescence Imaging of Cu(II) and Fe(III) in Living Systems

Chunfei Wang, Dandan Chen, Zixiang Wei, Jingyun Tan, Changfeng Wu*, Xuanjun Zhang*

**Experimental section**

**1. Materials**

Chemicals, such as 2,4-dimethyl-3-ethylpyrrole, phenylboronic acid and methylboronic acid and analytes containing NaCl, KCl, CaCl_2_ and MgSO_4_ were provided by Dieckman (Hong Kong) chemical industry company Ltd (Hong Kong, China). Fetal bovine serum (FBS) was purchased from Gibco/BRL (Grand Island, USA). Dulbecco’s modified eagle medium (DMEM) and other chemicals were obtained from Sigma-Aldrich (St. Louis, USA). Besides, all other reagents were AR grade and used without further purification. Distilled water was purified with a Milli-Q water purification system provided by Millipore (Bedford, USA).

**2. Instruments and general methods**

NMR spectra detection was recorded by a Bruker AV-400 instrument (Bruker, Germany) and reported in ppm downfield. High resolution mass spectra (HRMS) were obtained by a Xevo G2-XS QTof spectrometer (Waters, UK). All tested compounds were determined through a reverse phase C_18_ column by peak area integration (Agilent HPLC 1260, USA). Mass spectroscopy was also determined on a Q-exactive electrospray ionization mass spectrometer (Thermofisher, USA). UV/Vis’s absorption and fluorescent spectroscopy were measured by UV spectrophotometer (UV-1800, Shimadzu, Japan) and fluorescence spectrophotometer (FluoroMax-4, Horiba, Japan). Electron Paramagnetic Resonance (EPR) measurements were performed at Bruker EMXplus-10/12 spectrometer (Bruker, Germany). Images of cells and zebrafish were evaluated with confocal fluorescence microscope (LSM710, Carl Zeiss, Germany).

**3. Absorption and fluorescence spectra**

The optical characterization of NIR molecules was performed with UV/Vis absorption and fluorescent spectroscopy, which were measured by UV spectrophotometer (UV-1800, Shimadzu, Japan) and fluorescence spectrophotometer (FluoroMax-4, Horiba, Japan) in corresponding solvents.

**4. Preparation of test solutions**

Copper chloride (CuCl_2_) and ferric chloride (FeCl_3_) were dissolved in distilled water to 10 mM respectively. A stock solution (10 mM) of **NIRB1**–**NIRB6** were all prepared in DMSO. Take **NIRB1** as an example, test solutions were diluted **NIRB1** to 2 mL with a mixture of ethanol and distilled water (v/v = 2/1, containing 2% PEG 400) at a final concentration of 10 μM, while small aliquots of CuCl_2_ aqueous solution was gradually added at the final concentration up to 300 μM. The resulting solutions were shaken well and incubated for 1.5 h at room temperature for detecting UV-vis absorption and fluorescence property.

The solutions of various analytes were prepared in distilled water from NaCl, KCl, CaCl_2_, MgCl_2_, FeCl_3_, FeSO_4_, CuCl_2_, ZnCl_2_, NiCl_2_, MnCl_2_, InCl_3_, CoCl_2_, CdCl_2_, PbCl_2_, HgCl_2_, CuI respectively. Take **NIRB1** as an example, test solutions were diluted **NIRB1** to 2 mL with a mixture of ethanol and distilled water (v/v = 2/1, containing 2% PEG 400) at a final concentration of 10 μM, small aliquots of each testing species solution were added at specific concentration (500 μM for Na^+^, K^+^, and Ca^2+^, 100 μM for other metal ions). The resulting solutions were shaken well and incubated for 30 min at room temperature before recording the fluorescent spectra.

**5. pH stability of NIRB1, NIRB4, NIRB6, P1 and P2 with or without Cu(II)/Fe(III)**

A stock solution (10 mM) of **NIRB1**, **NIRB4**, **NIRB6**, **P1** and **P2** were all prepared in DMSO. To evaluate the pH stability of **NIRB1**, **NIRB4** and **NIRB6**, the resulting solution was prepared by dissolving small aliquots of **NIRB1**, **NIRB4** and **NIRB6** in different PBS buffers (pH = 4.42, 5.07, 6.32, 7.40) at a final concentration of 10 μM. The absorption changes with different pH values were recorded. In addition, small aliquots of **P1** and **P2** were diluted with different PBS buffers (pH = 4.42, 5.07, 6.32, 7.40) at a final concentration of 10 μM. Then the fluorescence changes of **P1** (641 nm) and **P2** (625 nm) with different pH were recorded. The excitation wavelength was 580 nm.

The resulting solution was prepared by dissolving small aliquots of **NIRB1**, **NIRB4** and **NIRB6** in the mixture of ethanol and PBS buffer solution with different pH (v/v = 2:1, pH = 4.42, 5.07, 6.32, 7.40) at final concentration of 10 μM adding with CuCl_2_ or FeCl_3_ (50 μM). The fluorescence changes at 635 nm (**NIRB1**), 630 nm (**NIRB4**) and 628 nm (**NIRB6**) with different pH were record. Moreover, small aliquots of **P1** and **P2** in the mixture of ethanol and PBS buffer solution with different pH (v/v = 2:1, pH = 4.42, 5.07, 6.32, 7.40) at final concentration of 10 μM, gradually adding with CuCl_2_ or FeCl_3_ (0−300 μM). Then, the fluorescence changes of **P1** (641 nm) and **P2** (625 nm) were record. The excitation wavelength was 580 nm.

**6. Detection of P1 by HPLC**

The catalytical reaction was performed in the PBS buffer between 10 μM **NIRB1** and different concentration of CuCl_2_/FeCl_3_ (2, 5, 10, 30 and 50 μM). After reaction for 3 h, the reaction mixture was freeze-dried to get the powder for HPLC analysis. In addition, cells treated with different concentration of CuCl_2_/FeCl_3_ (2, 5, 10, 30 and 50 μM) for 30 min, and then treated with 10 μM **NIRB1** for 3 h. After that, cells were washed with PBS three times and lysed by cell lysis buffer. Then the deproteinization of cell lysis was conducted by methanol (V/V = 1 : 3) and concentrated by Termovap Sample Concentrator to get the powder for HPLC analysis.

**Table S1** The relative proportion of red emitted products (**P1**) in the reaction conducted in PBS and extraction from cell lysis.

|  | NIRB1 (μM) | PBS | Cell lysis |
| --- | --- | --- | --- |
|  |  | Relative proportion of P1 (%) | Relative proportion of P1 (%) |
| CuCl_2_ | 2 | 14.41 | Not find |
|  | 5 | 17.11 | 10.06 |
|  | 10 | 23.71 | 18.43 |
|  | 30 | 43.06 | 21.03 |
|  | 50 | 49.48 | 27.16 |
| FeCl_3_ | 2 | 0.45 | Not find |
|  | 5 | 8.44 | 2.78 |
|  | 10 | 15.64 | 10.95 |
|  | 30 | 28.51 | 14.10 |
|  | 50 | 34.78 | 14.27 |

**Notice:** “Not find” here means that there displays very low content that the instrument cannot detect, which is out of the limit of detection.

**Scheme S1**. Synthetic route of **NIRB1−NIRB6**.


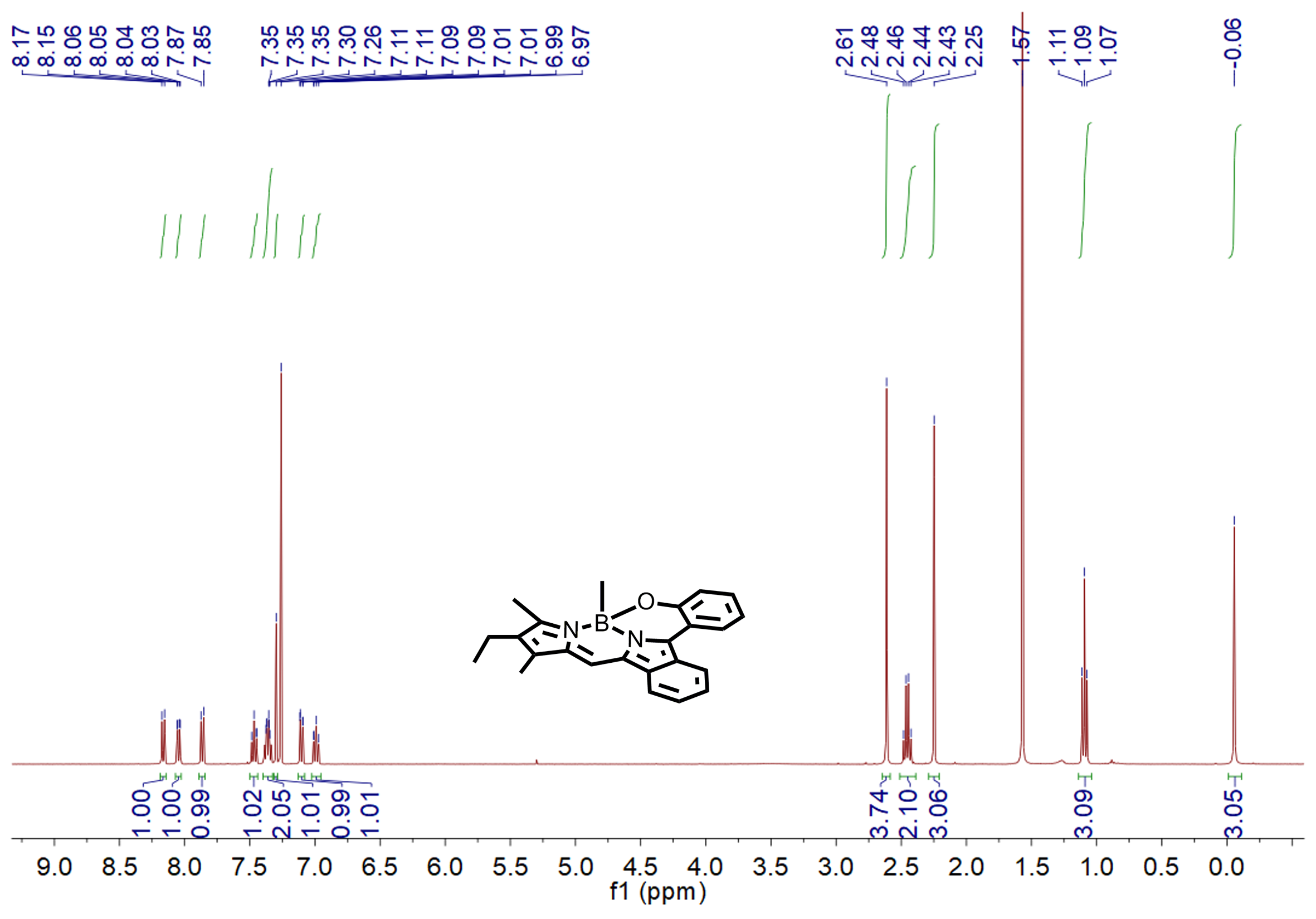


**Figure S1.** ^1^H NMR spectrum of **BOBPY1** in CDCl_3_.


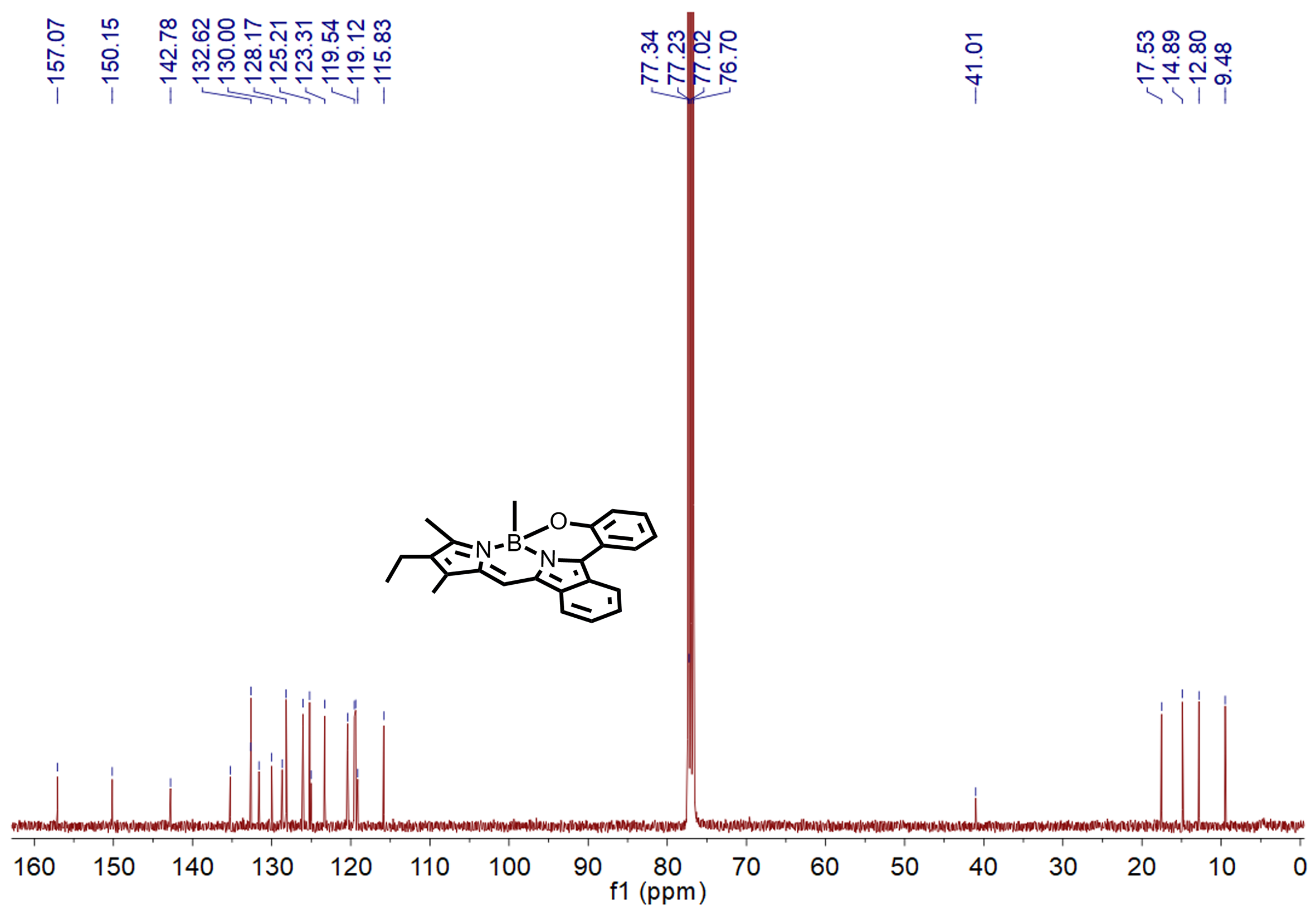


**Figure S2.** ^13^C NMR spectrum of **BOBPY1** in CDCl_3_.


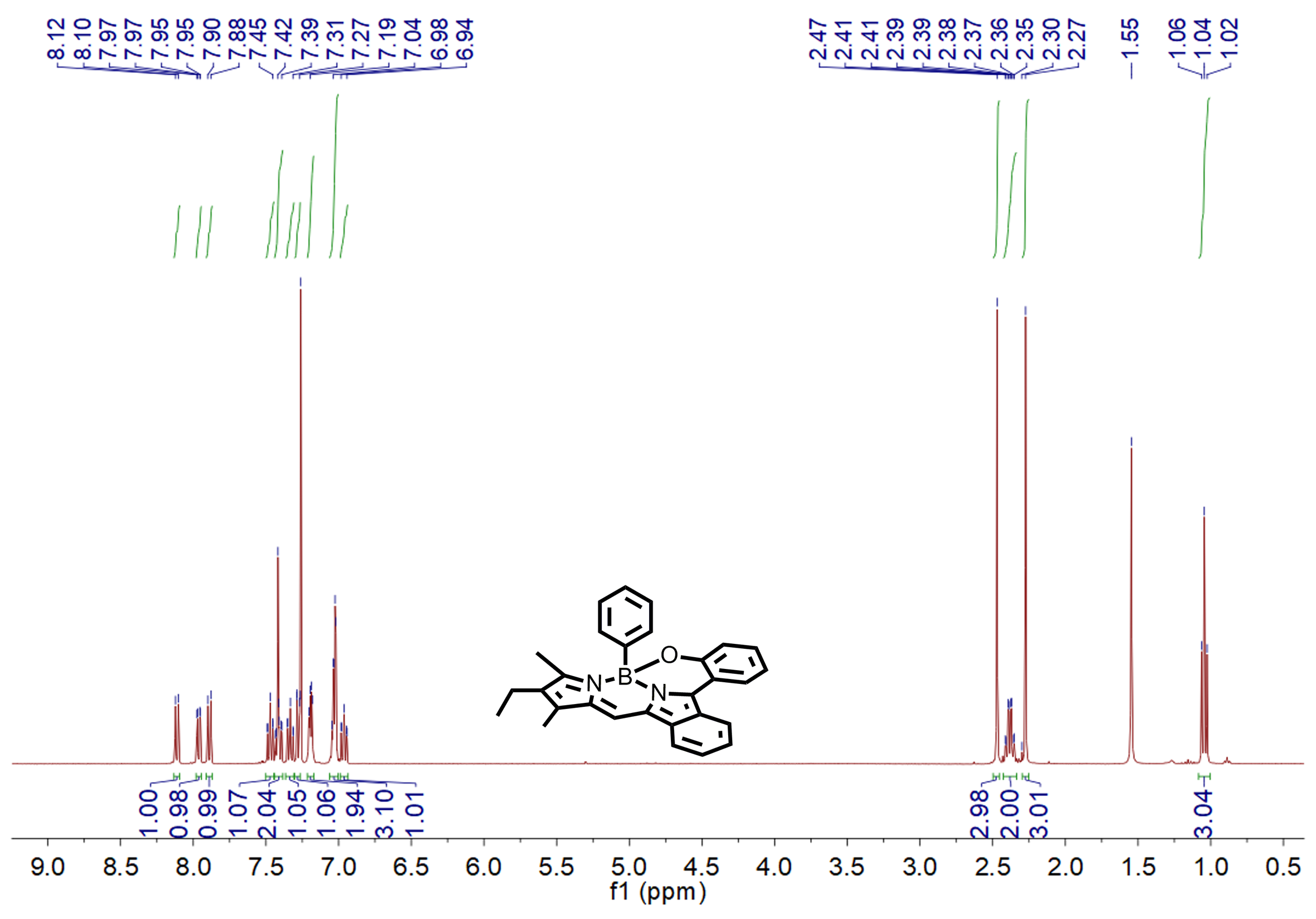


**Figure S3.** ^1^H NMR spectrum of **BOBPY2** in DMSO.


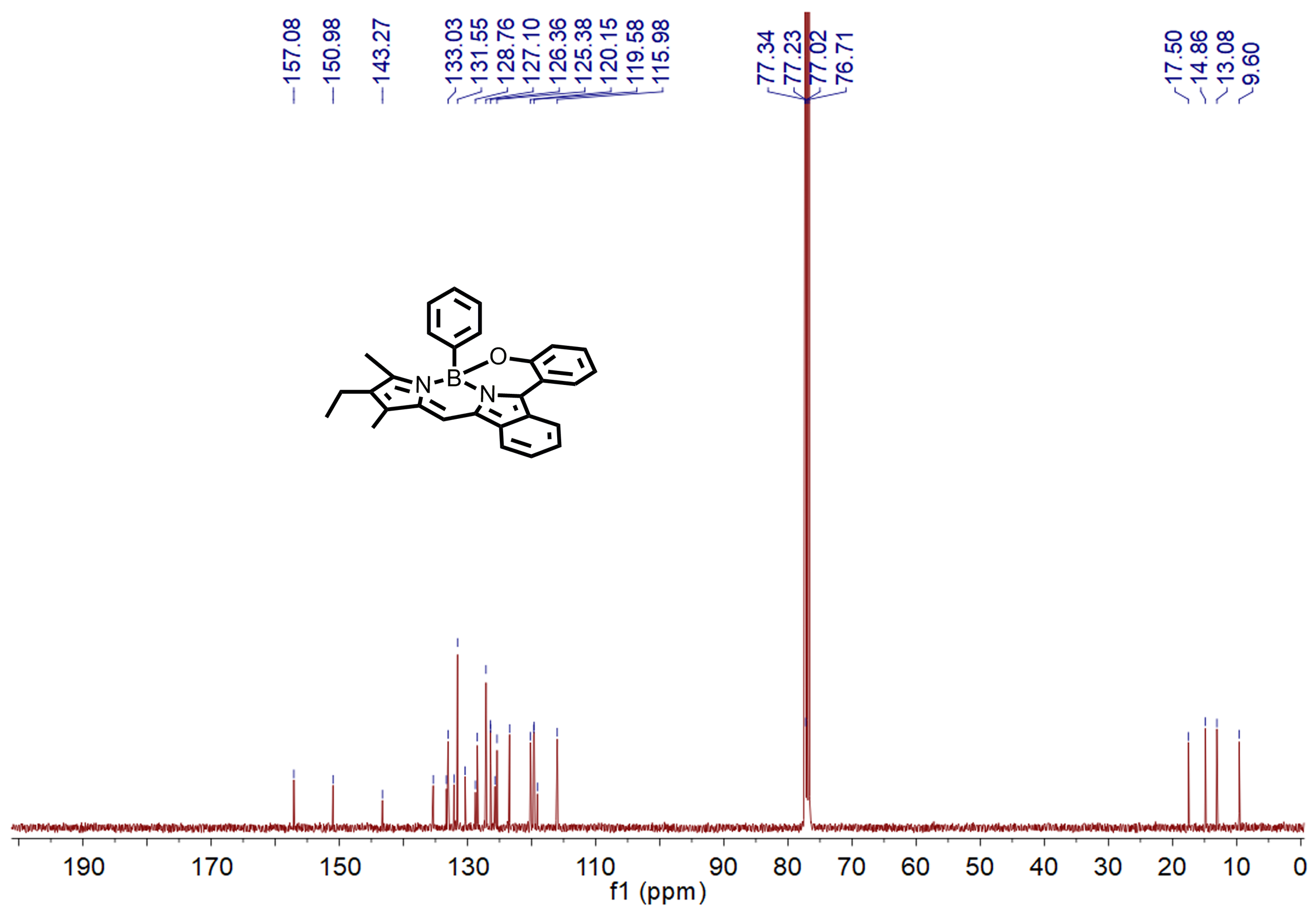


**Figure S4.** ^13^C NMR spectrum of **BOBPY2** in DMSO.


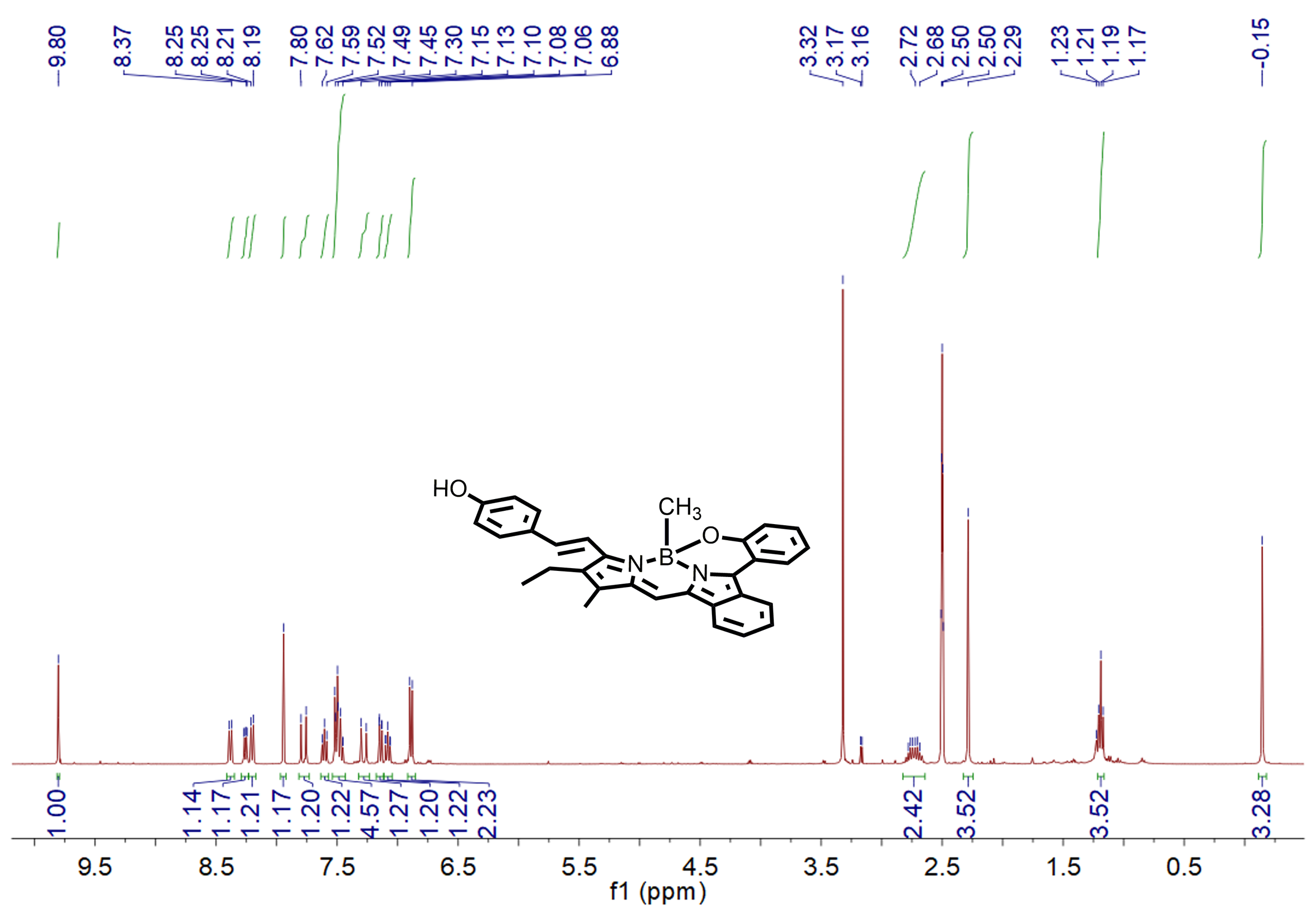


**Figure S5.** ^1^H NMR spectrum of **NIRB1** in DMSO.


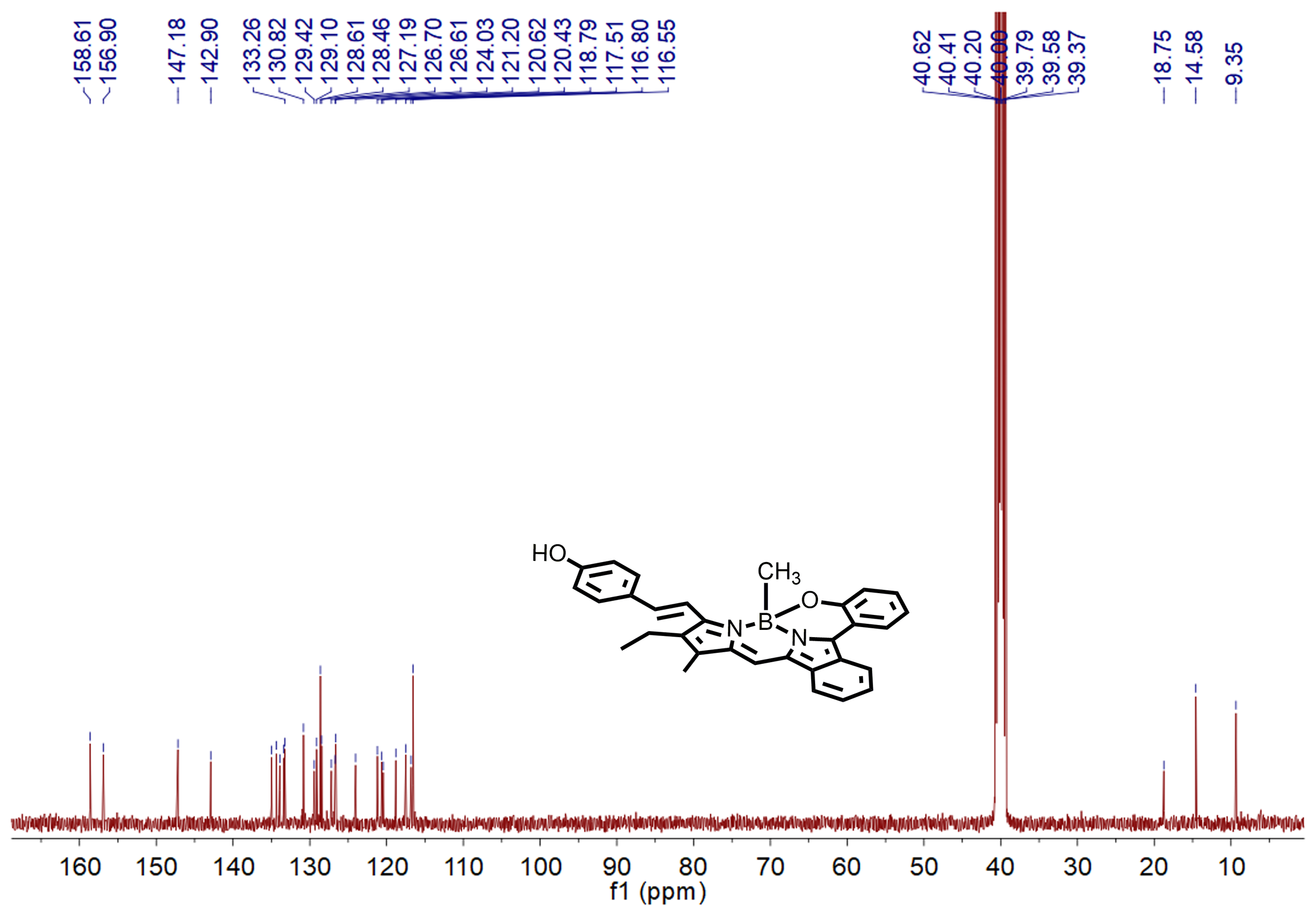


**Figure S6.** ^13^C NMR spectrum of **NIRB1** in DMSO.


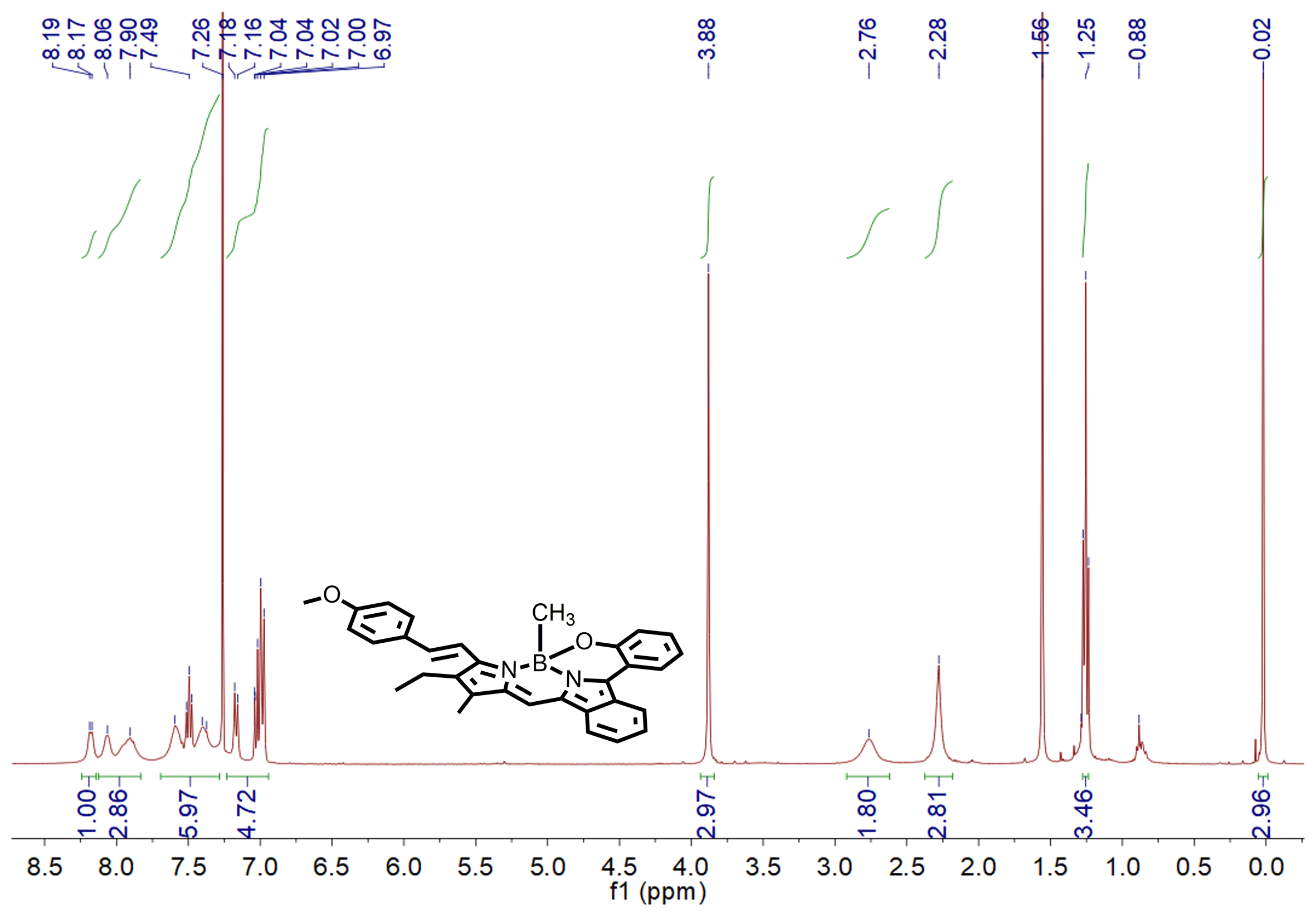


**Figure S7.** ^1^H NMR spectrum of **NIRB2** in CDCl_3_.


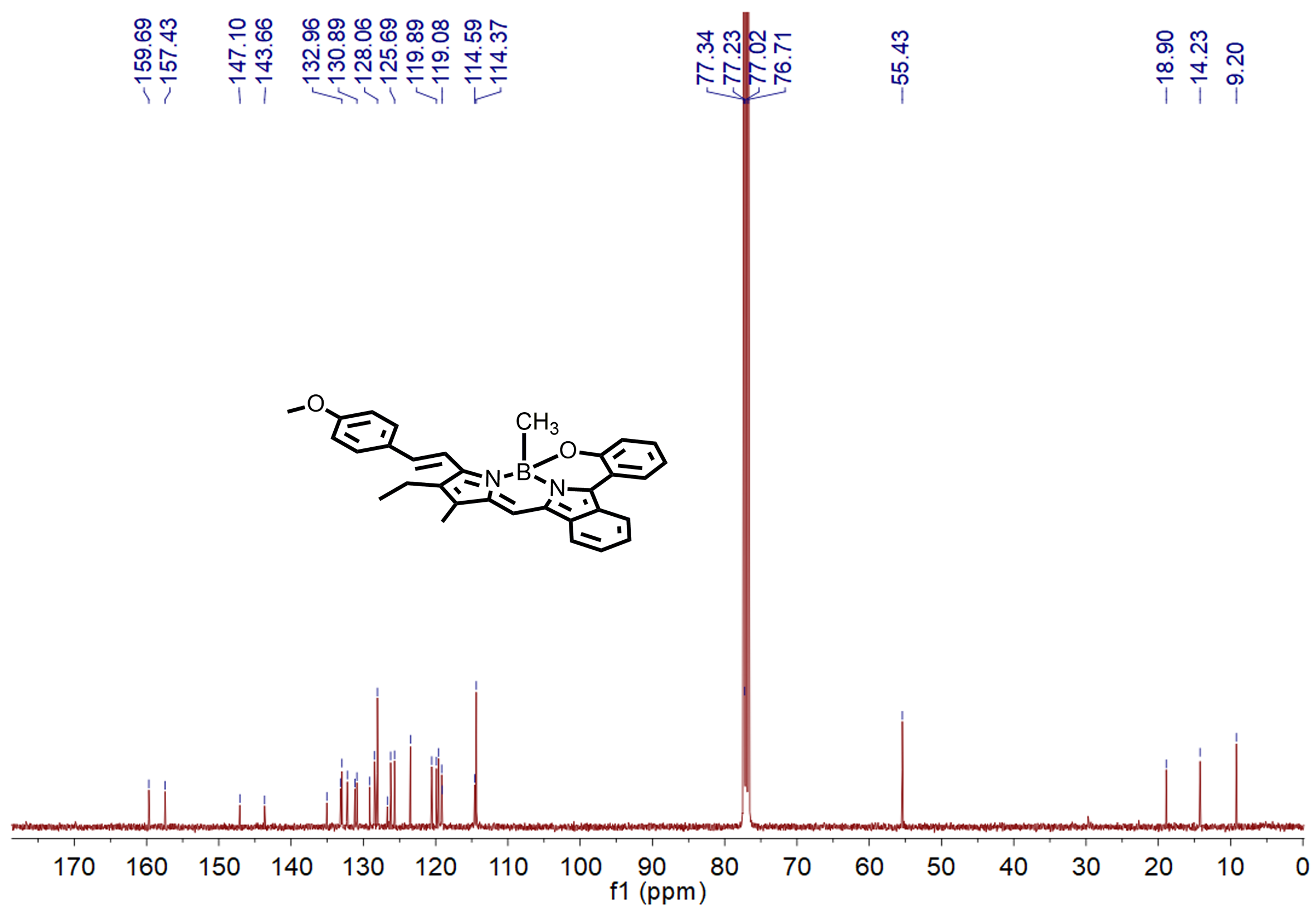


**Figure S8.** ^13^C NMR spectrum of **NIRB2** in CDCl_3_.


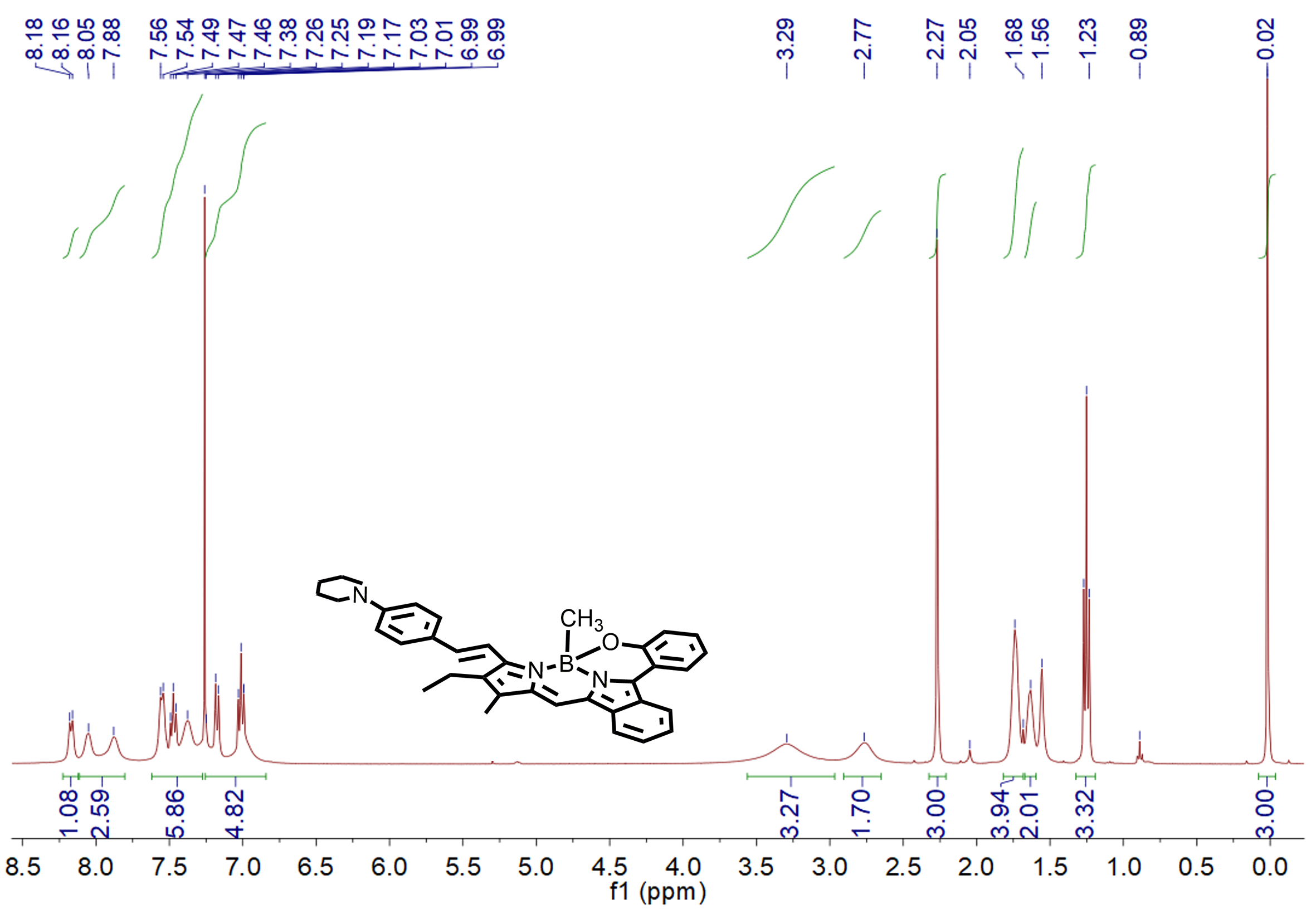


**Figure S9.** ^1^H NMR spectrum of **NIRB3** in CDCl_3_.


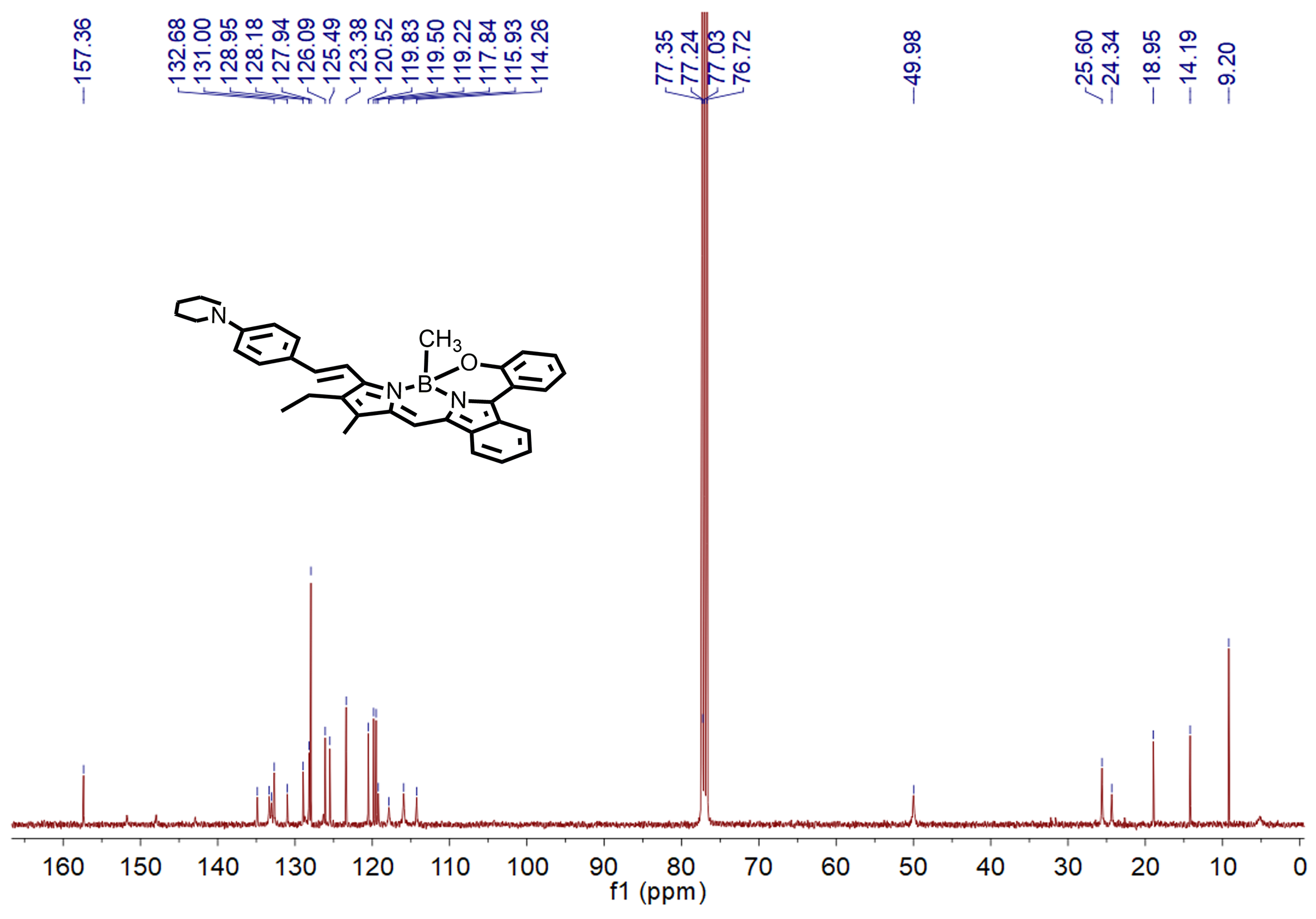


**Figure S10.** ^13^C NMR spectrum of **NIRB3** in CDCl_3_.


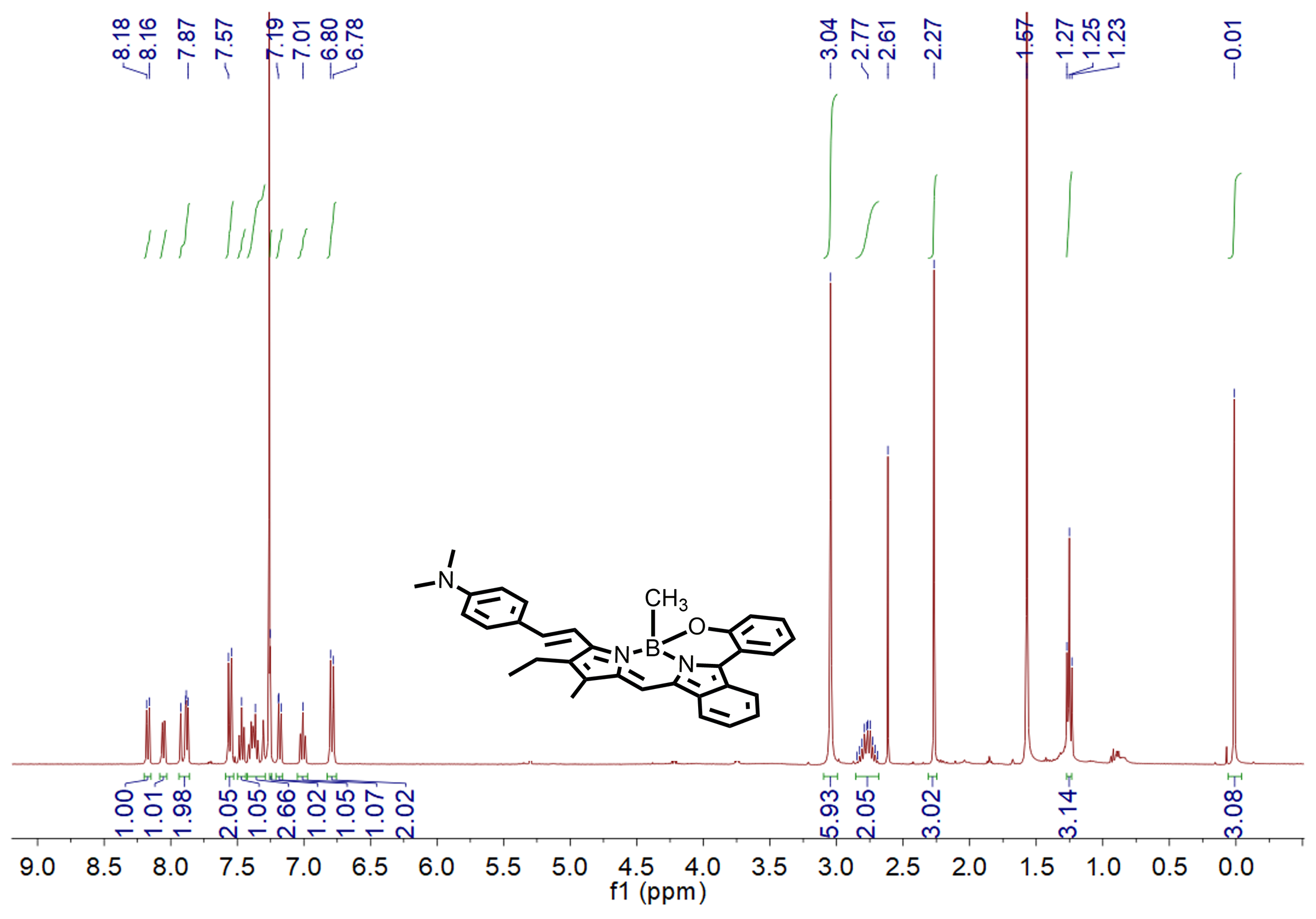


**Figure S11.** ^1^H NMR spectrum of **NIRB4** in CDCl_3_.


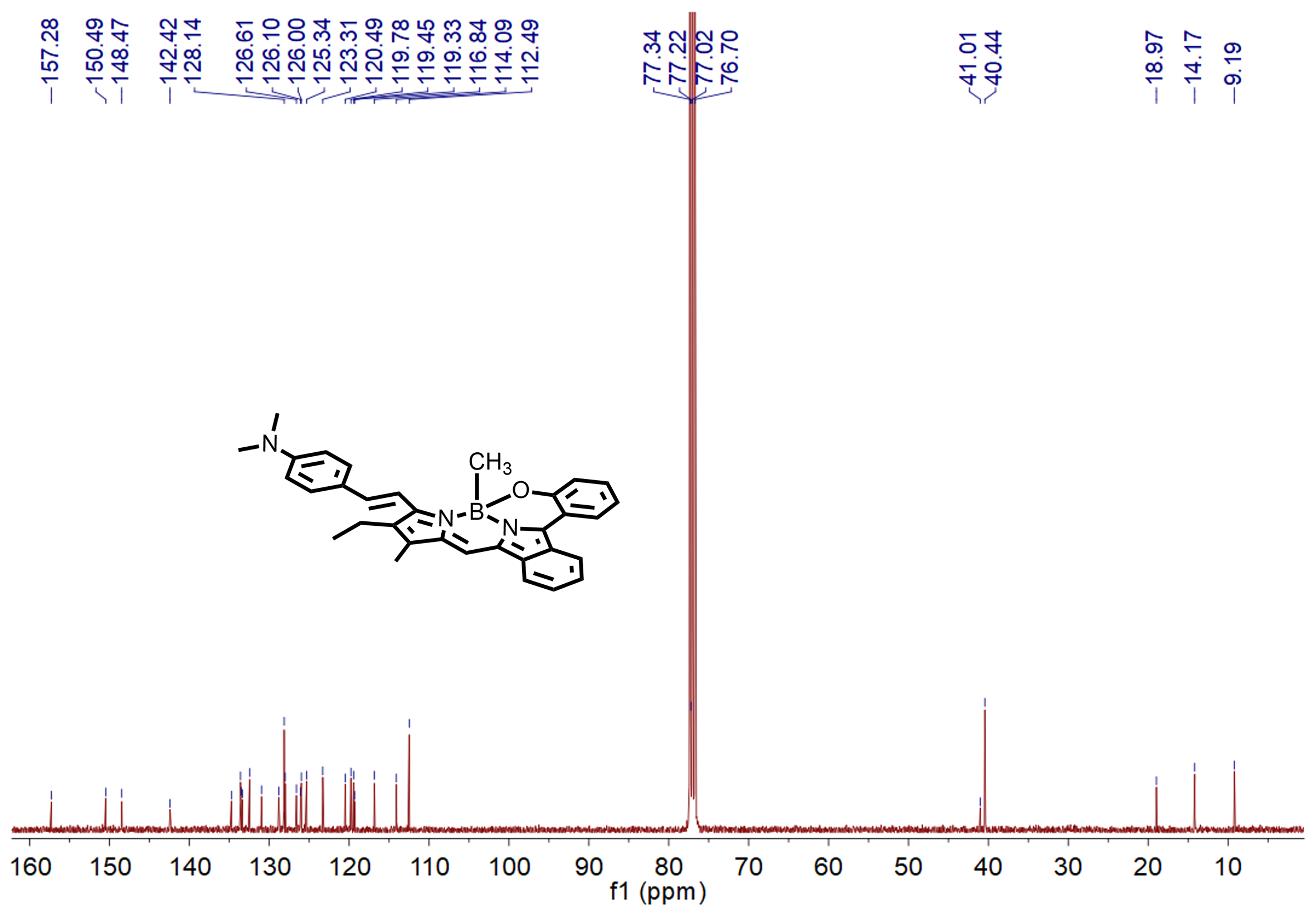


**Figure S12.** ^13^C NMR spectrum of **NIRB4** in CDCl_3_.


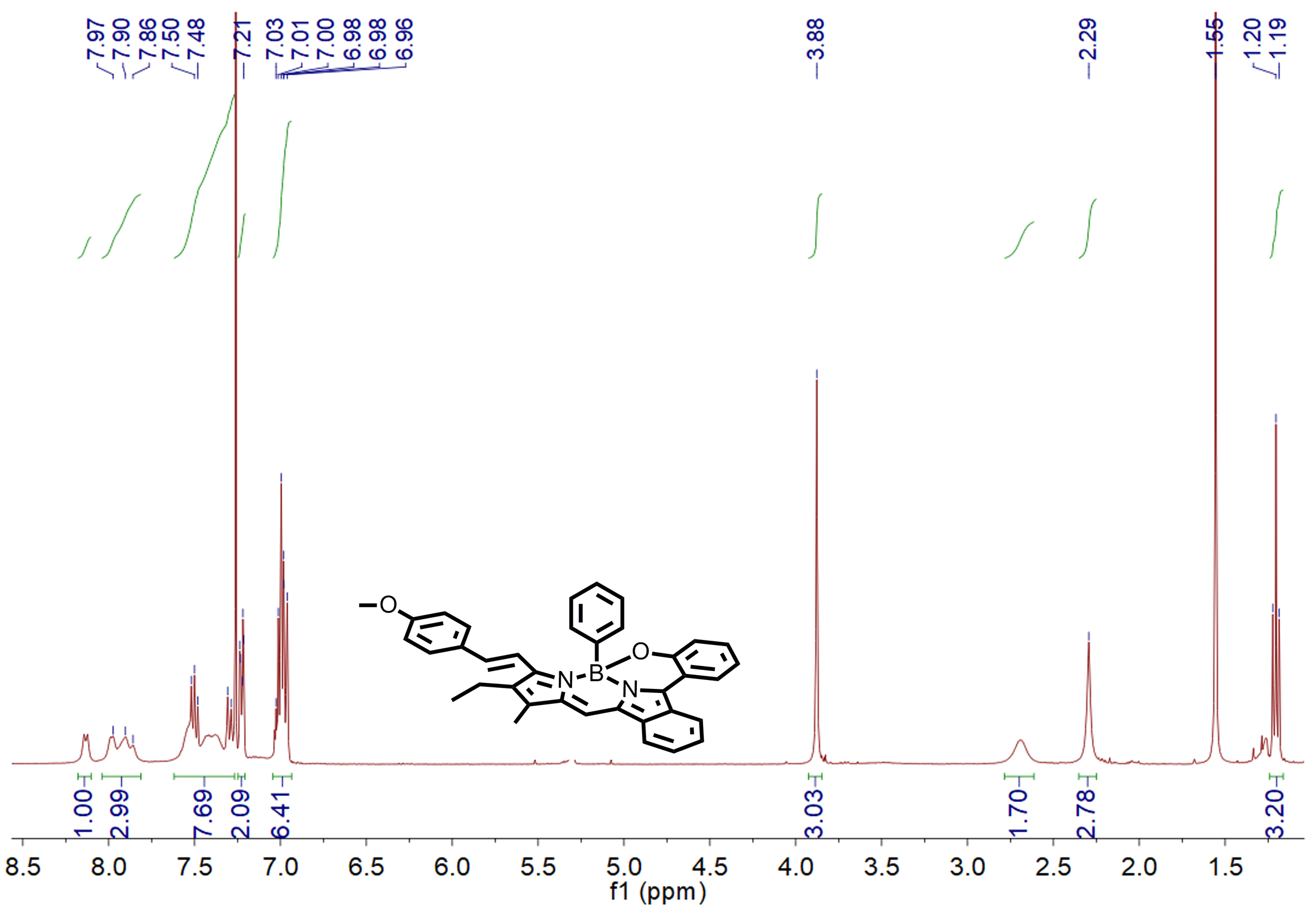


**Figure S13.** ^1^H NMR spectrum of **NIRB5** in CDCl_3_.


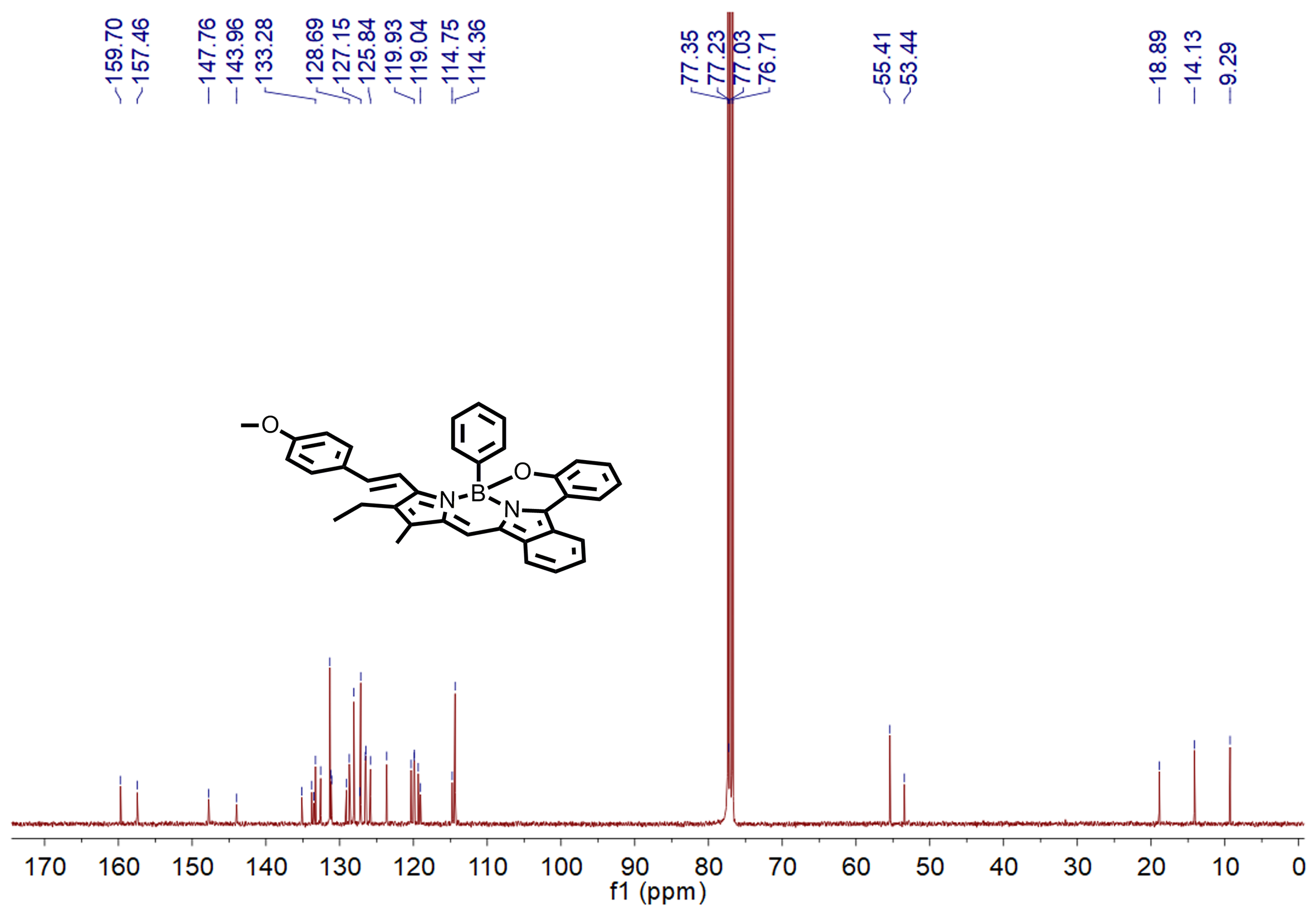


**Figure S14.** ^13^C NMR spectrum of **NIRB5** in CDCl_3_.


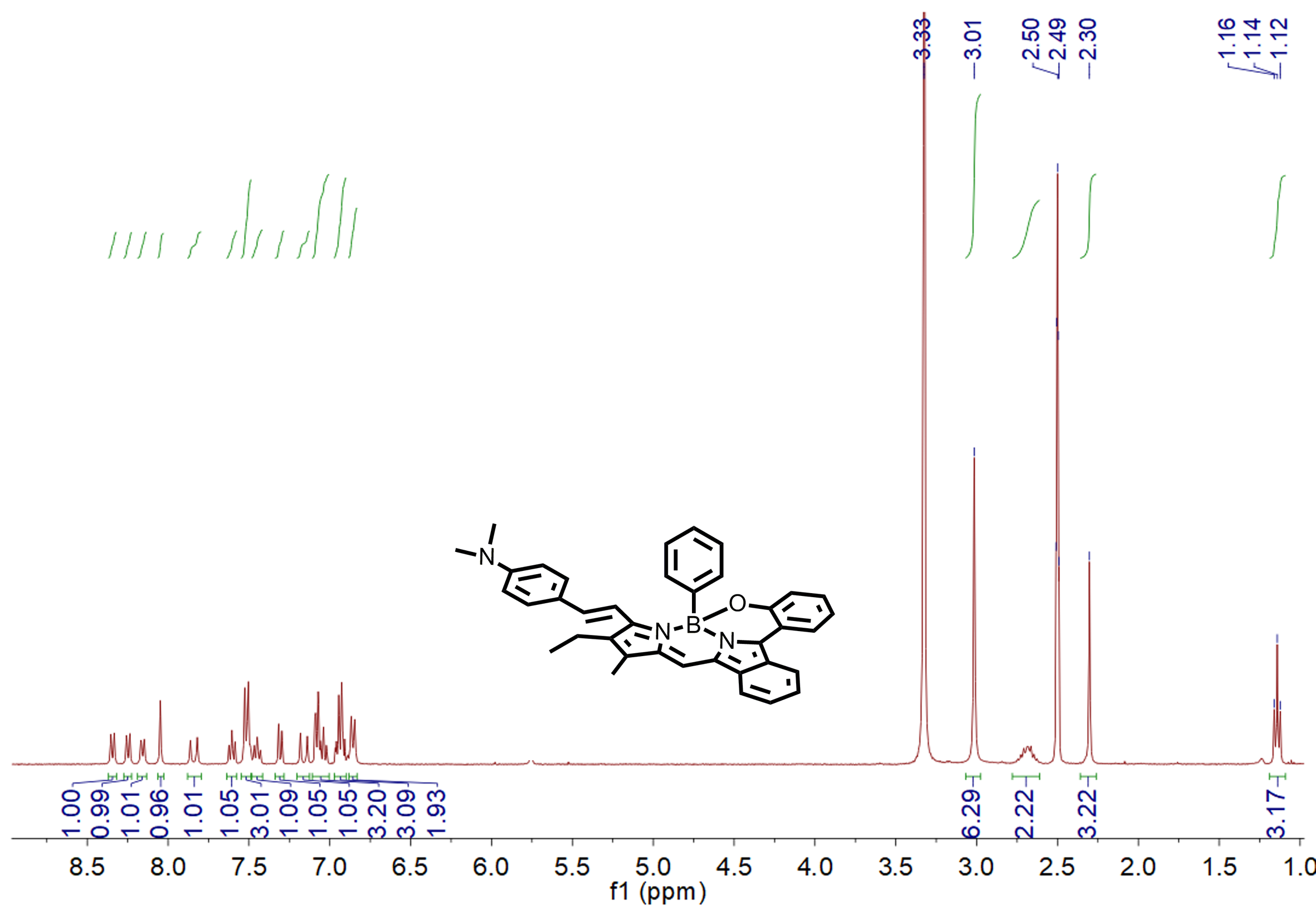


**Figure S15.** ^1^H NMR spectrum of **NIRB6** in CDCl_3_.


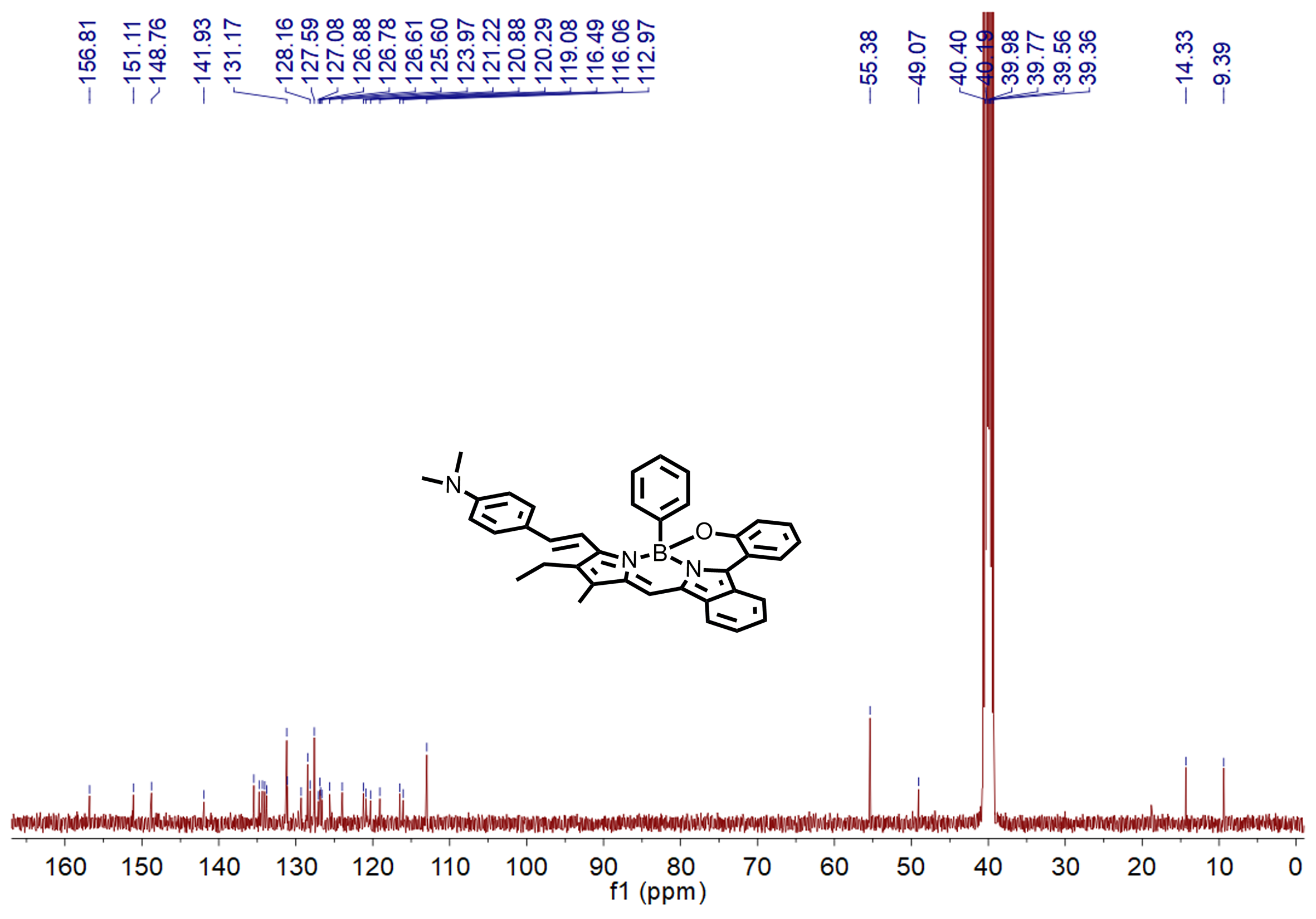


**Figure S16.** ^13^C NMR spectrum of **NIRB6** in CDCl_3_.


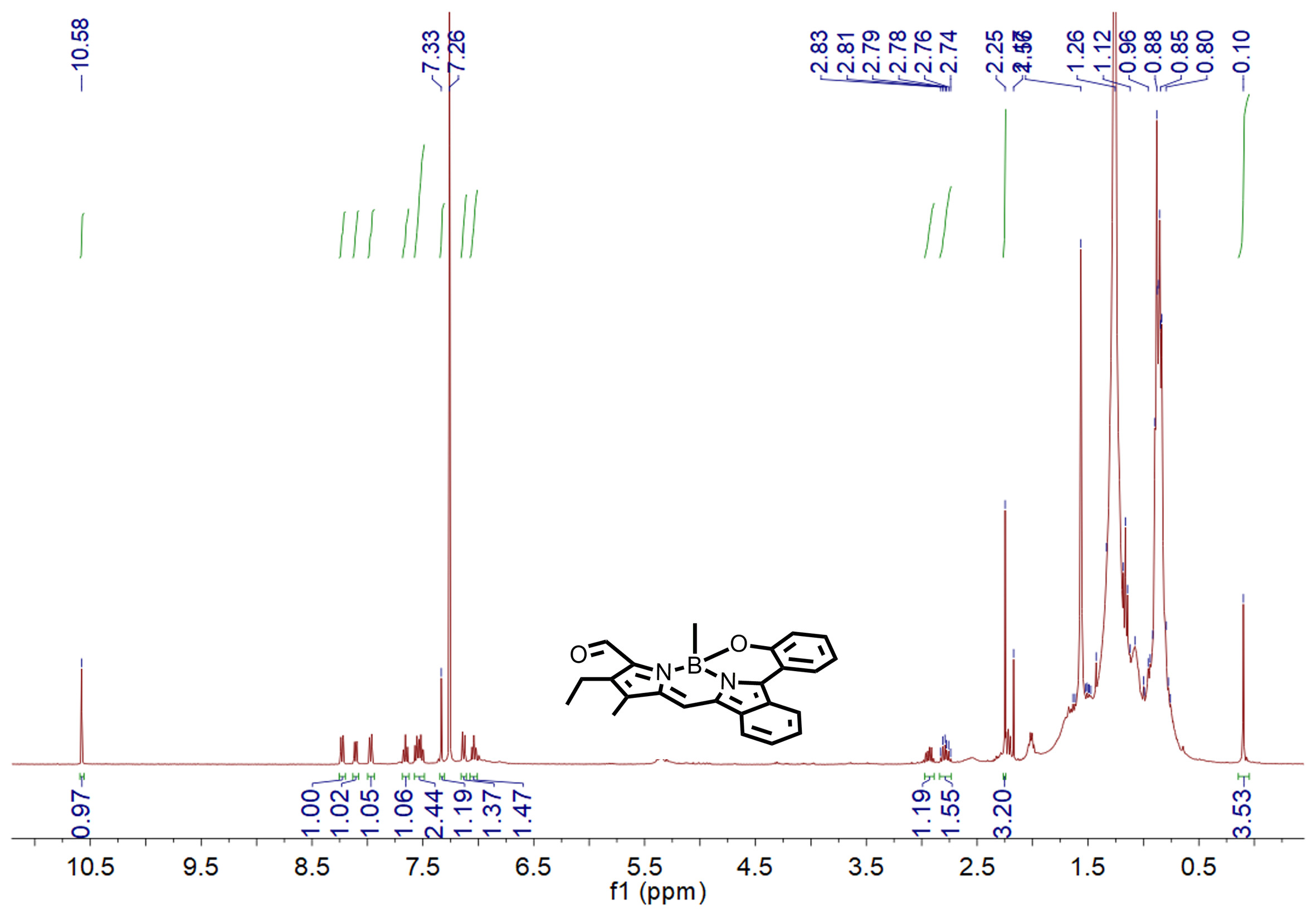


**Figure S17.** ^1^H NMR spectrum of red emitting product **P1** in CDCl_3_.


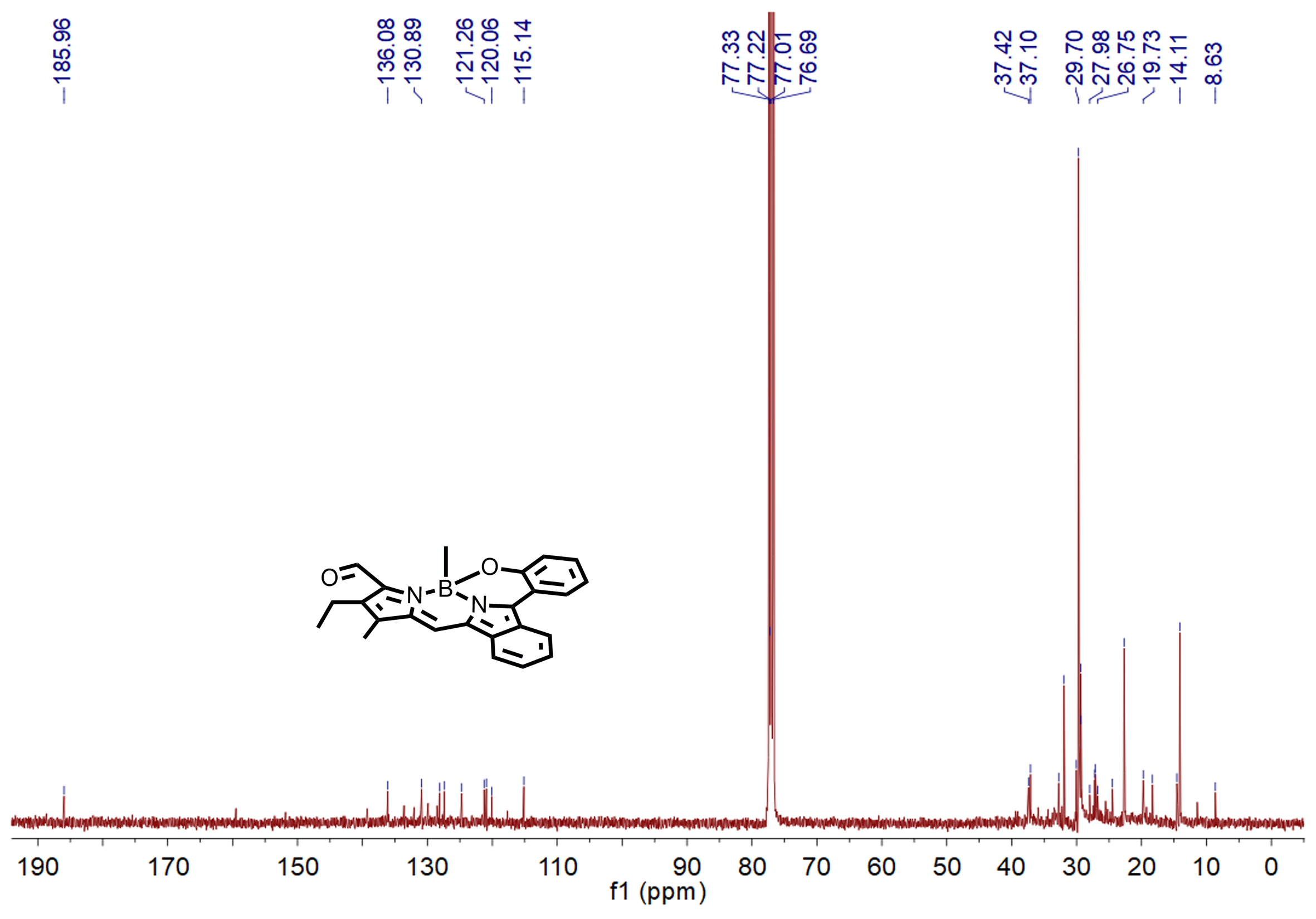


**Figure S18.** ^13^C NMR spectrum of red emitting product **P1** in CDCl_3_.


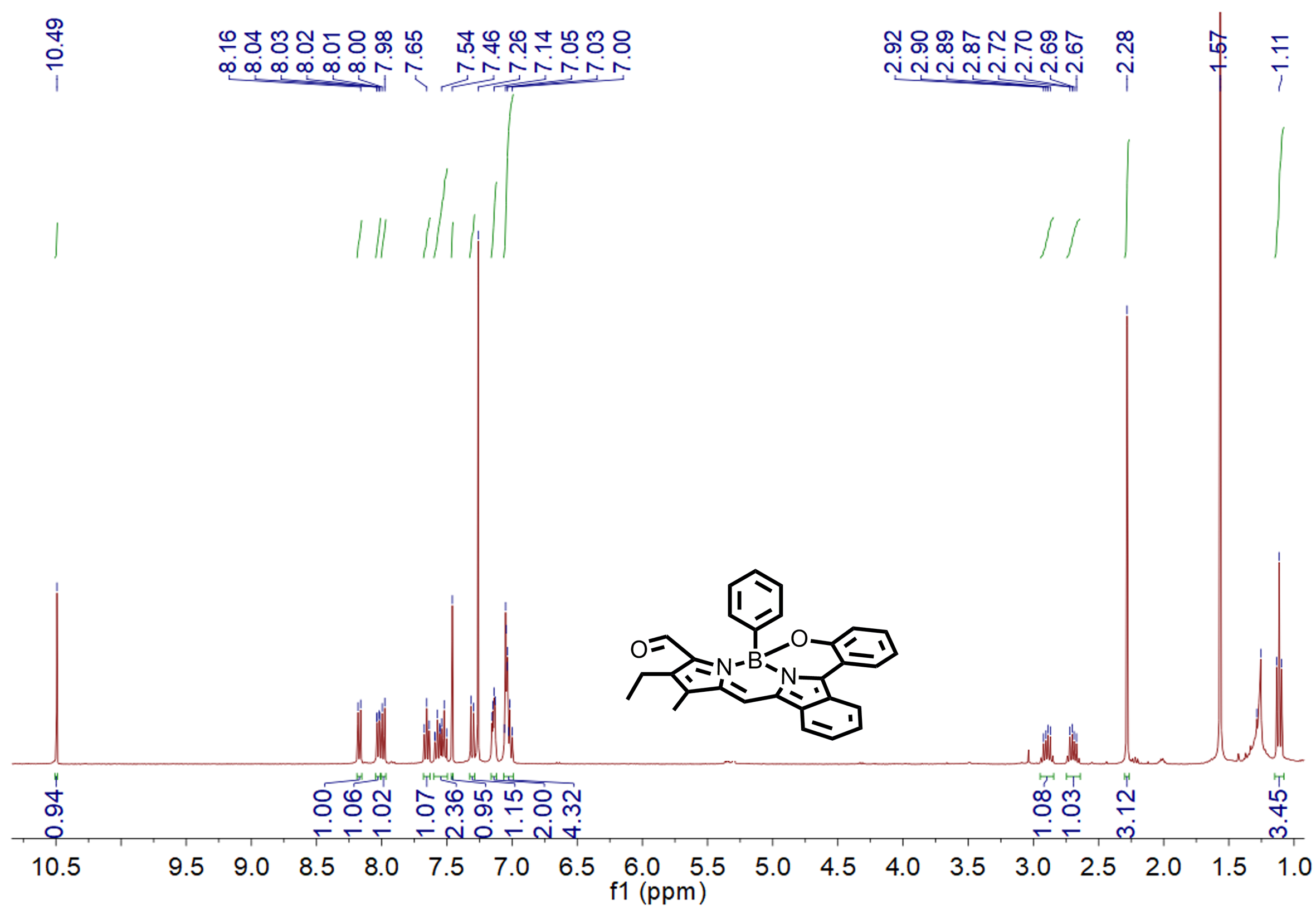


**Figure S19.** ^1^H NMR spectrum of red emitting product **P2** in CDCl_3_.


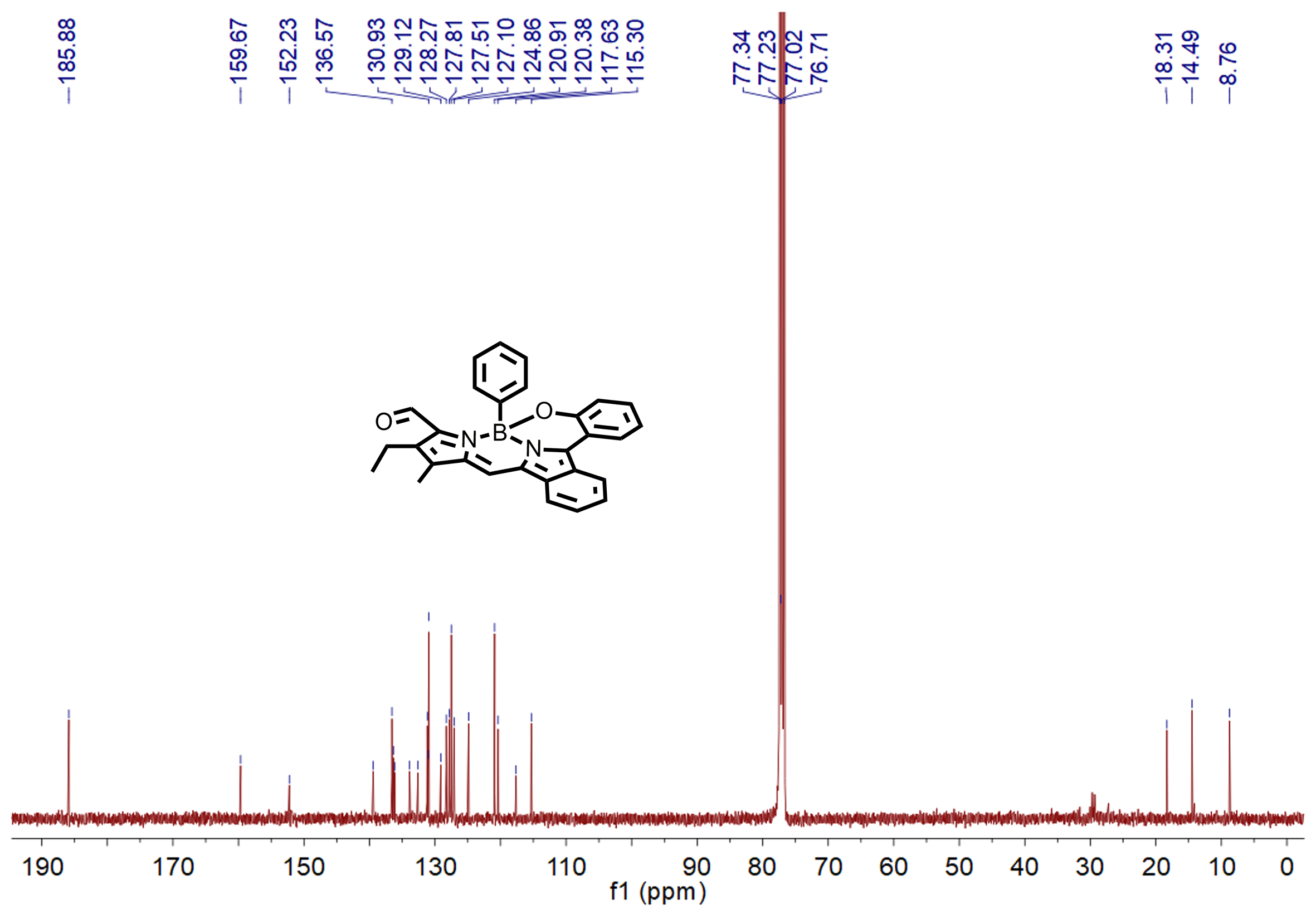


**Figure S20.** ^13^C NMR spectrum of red emitting product **P2** in CDCl_3_.


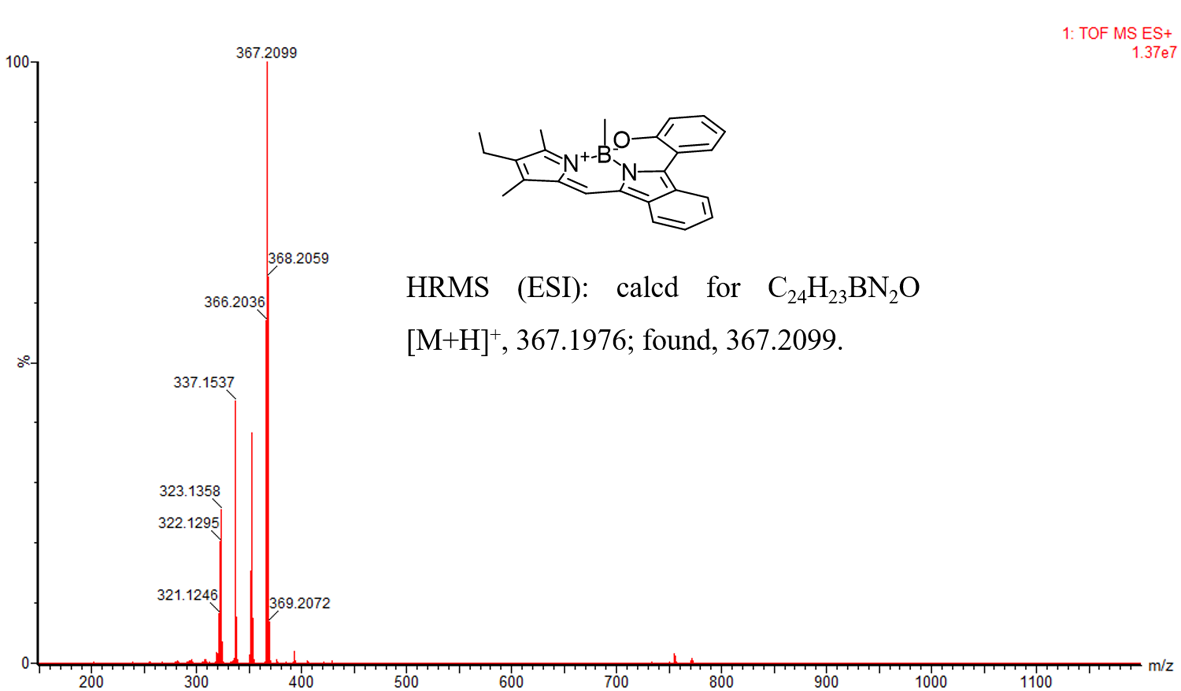


**Figure S21.** HRMS spectrum of **BOBPY2**.


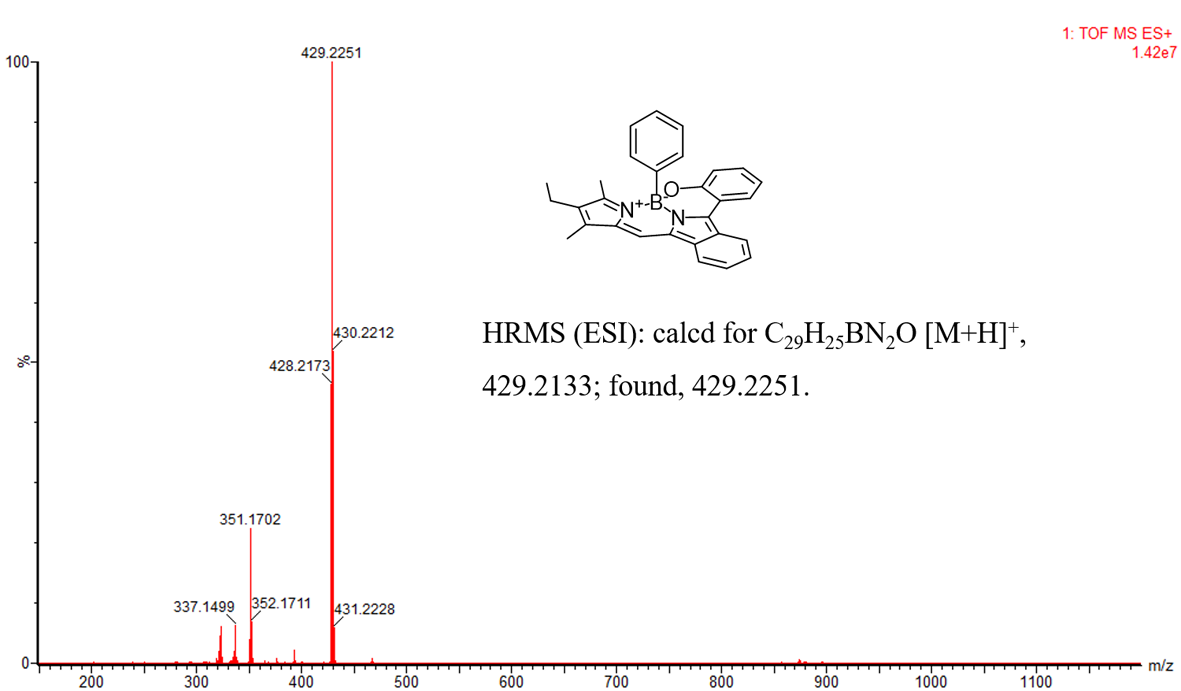


**Figure S22.** HRMS spectrum of **BOBPY2**.


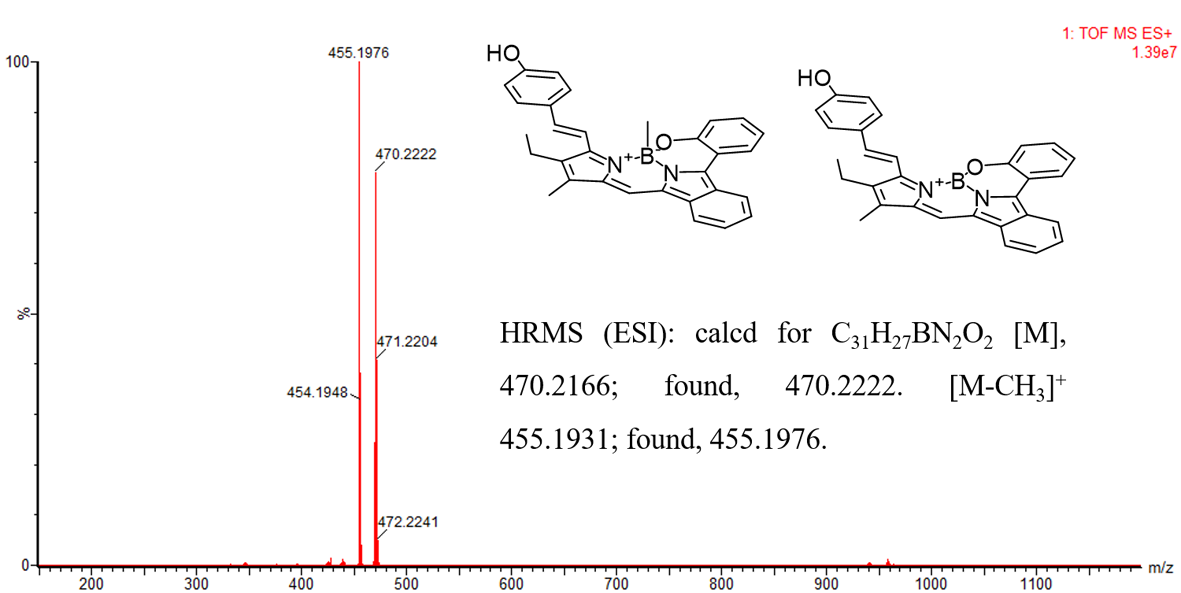


**Figure S23.** HRMS spectrum of **NIRB1**.


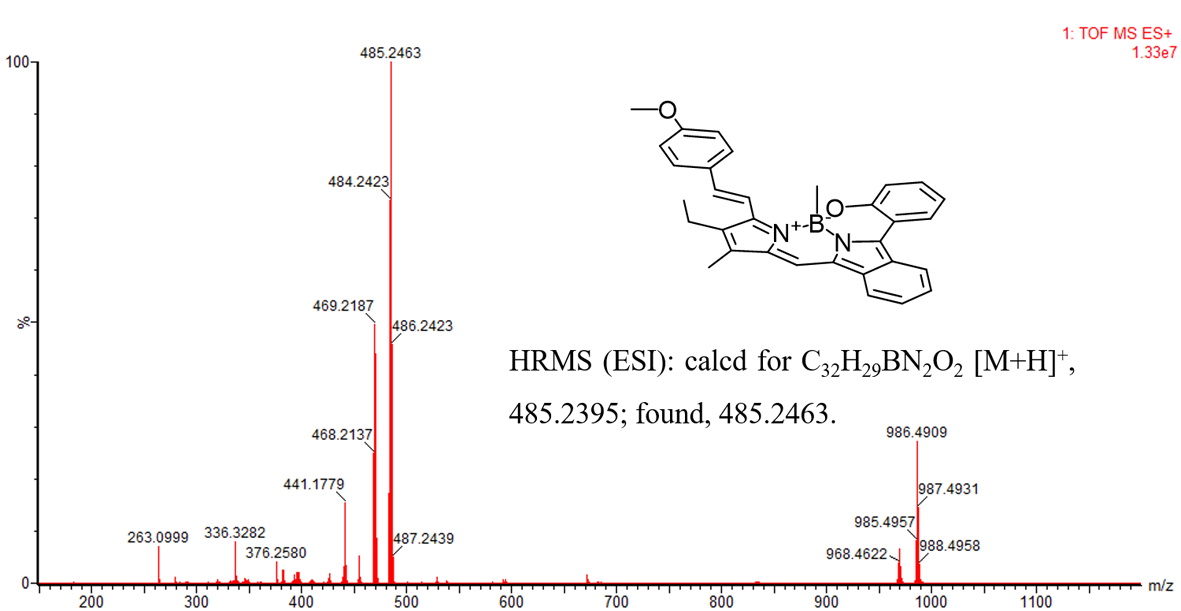


**Figure S24.** HRMS spectrum of **NIRB2_._**


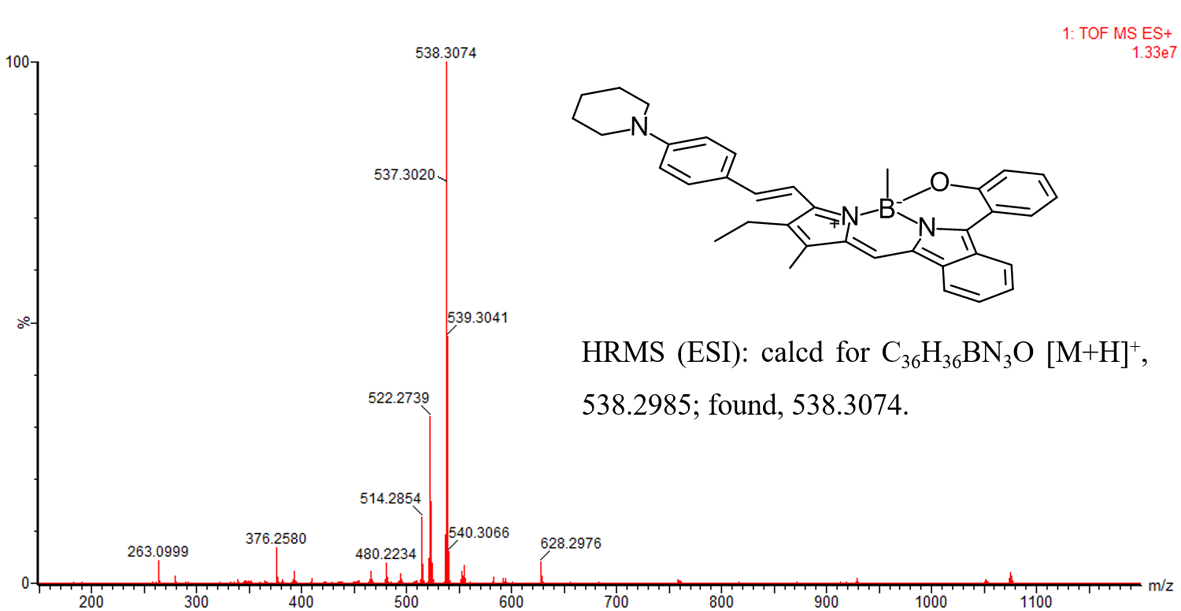


**Figure S25.** HRMS spectrum of **NIRB3**.


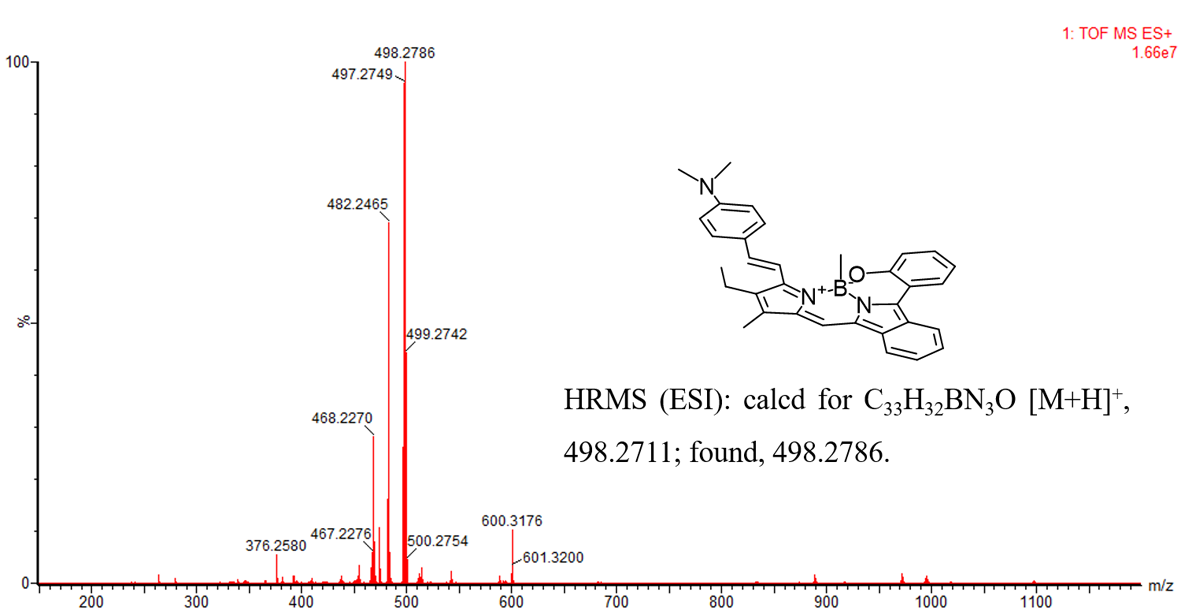


**Figure S26.** HRMS spectrum of **NIRB4**.


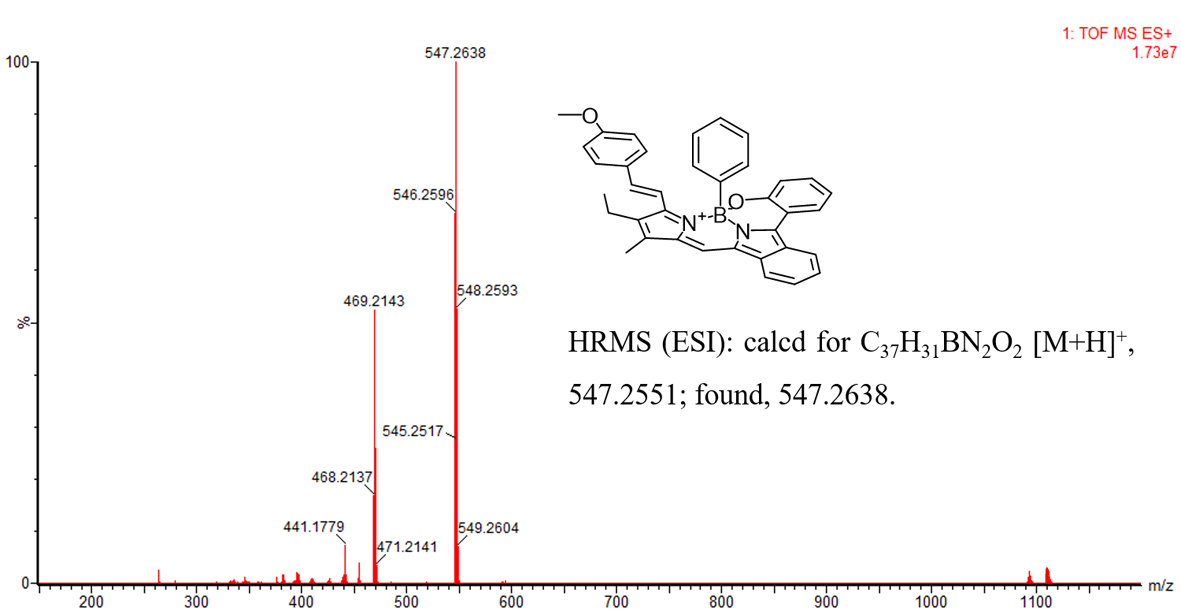


**Figure S27.** HRMS spectrum of **NIRB5**.


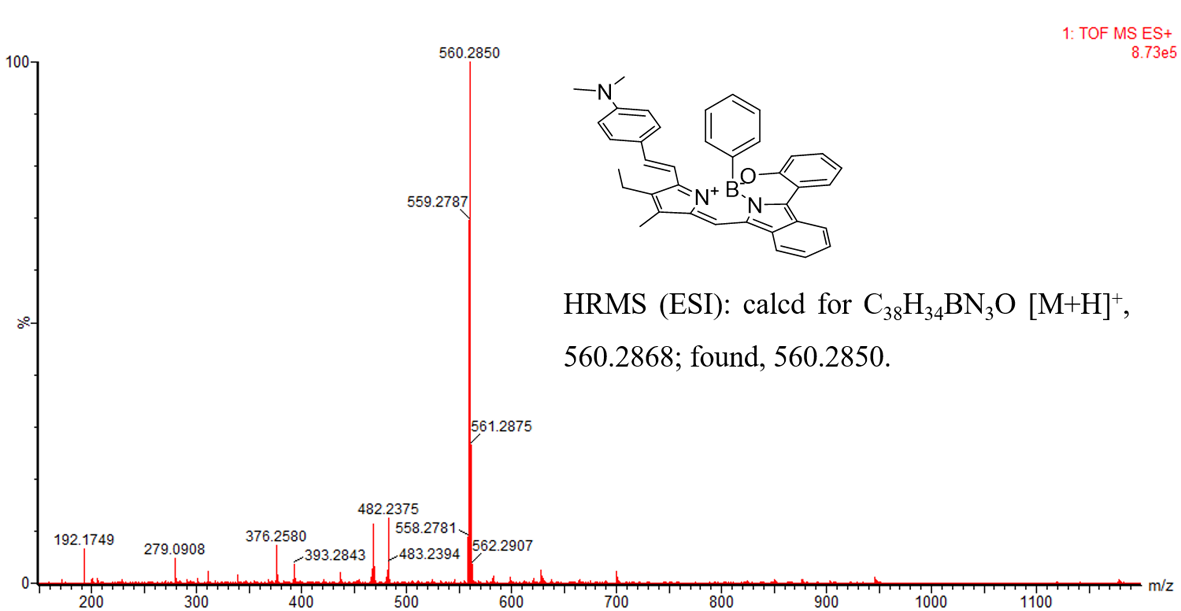


**Figure S28.** HRMS spectrum of **NIRB6**.


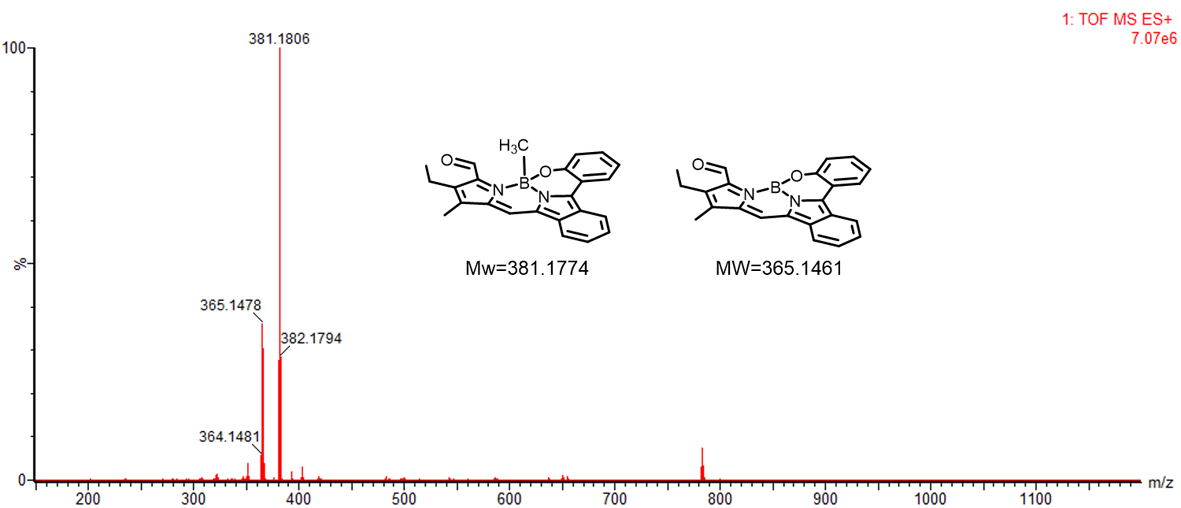


**Figure S29.** HRMS spectrum of red emitting product **P1_._**


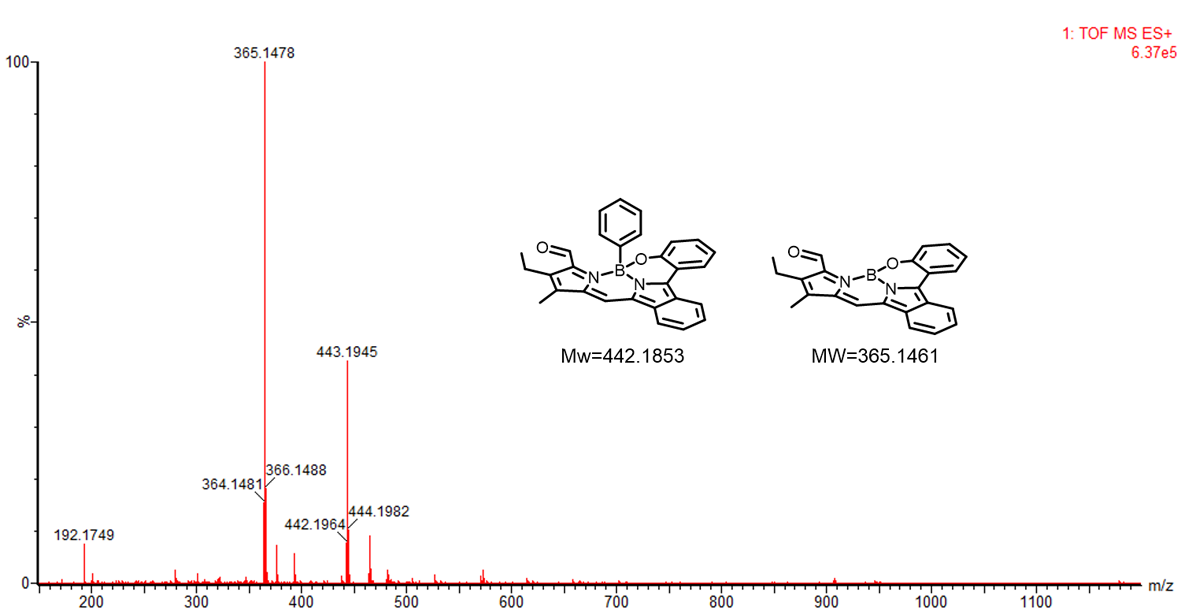


**Figure S30.** HRMS spectrum of red emitting product **P2**.


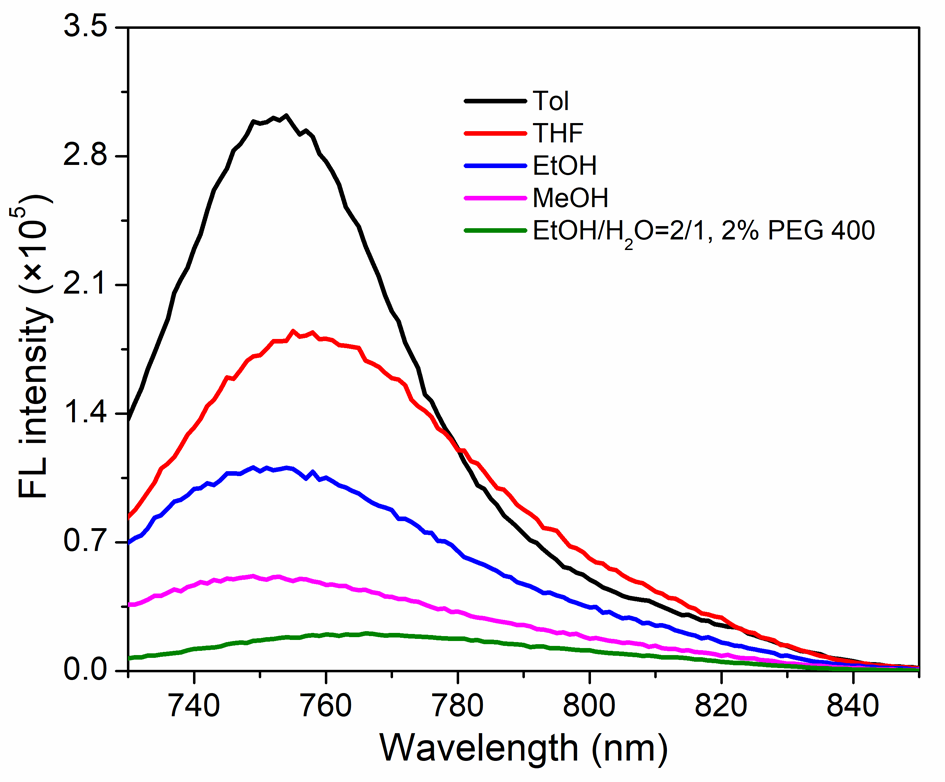


**Figure S31.** Fluorescence spectra of **NIRB6** (10 μM) in different solvents, λ_ex_ = 660 nm.


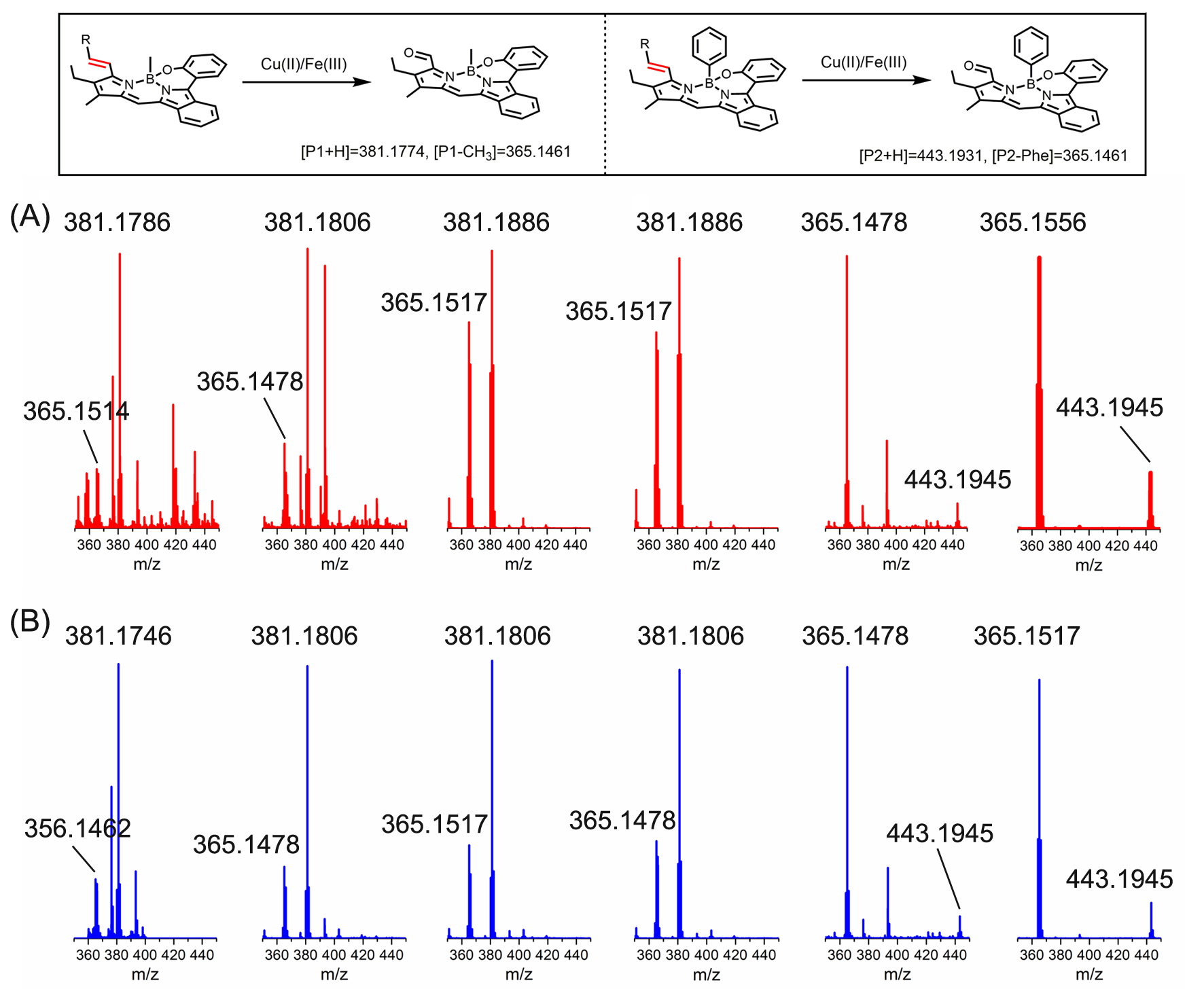


**Figure S32.** MS data of the reaction mixture between NIR molecules and CuCl_2_ (A) or FeCl_3_ (B) in a mixture of ethanol and H_2_O (v/v = 2:1, containing 2% PEG400). From left to right are MS spectra of **NIRB1**−**NIRB6** after addition of CuCl_2_ or FeCl_3_.


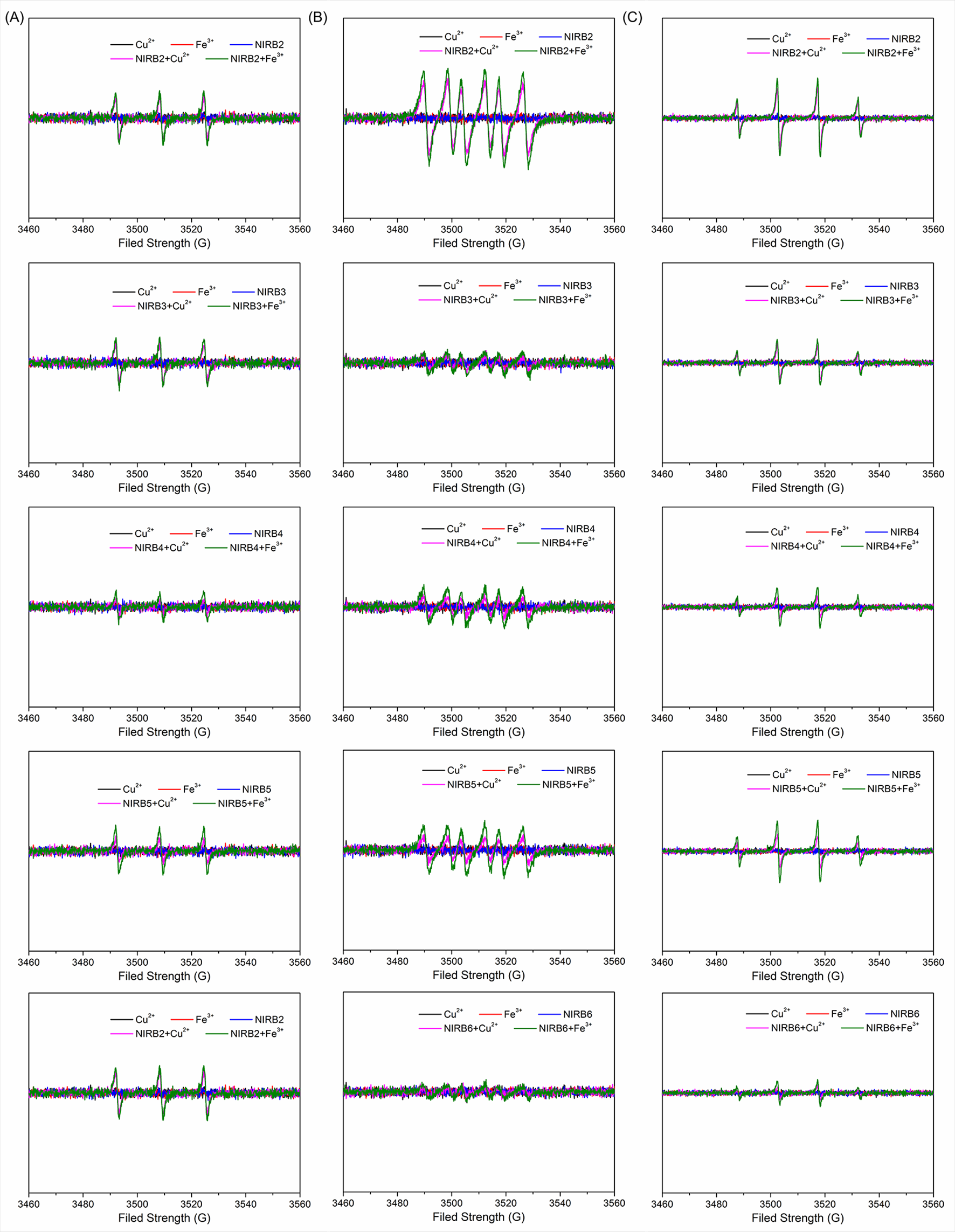


**Figure S33.** EPR spectra of **NIRB2**−**NIRB6** with different spin trap for the detection of ^1^O_2_ (A), O_2_^•−^ (B) and ^•^OH (C).


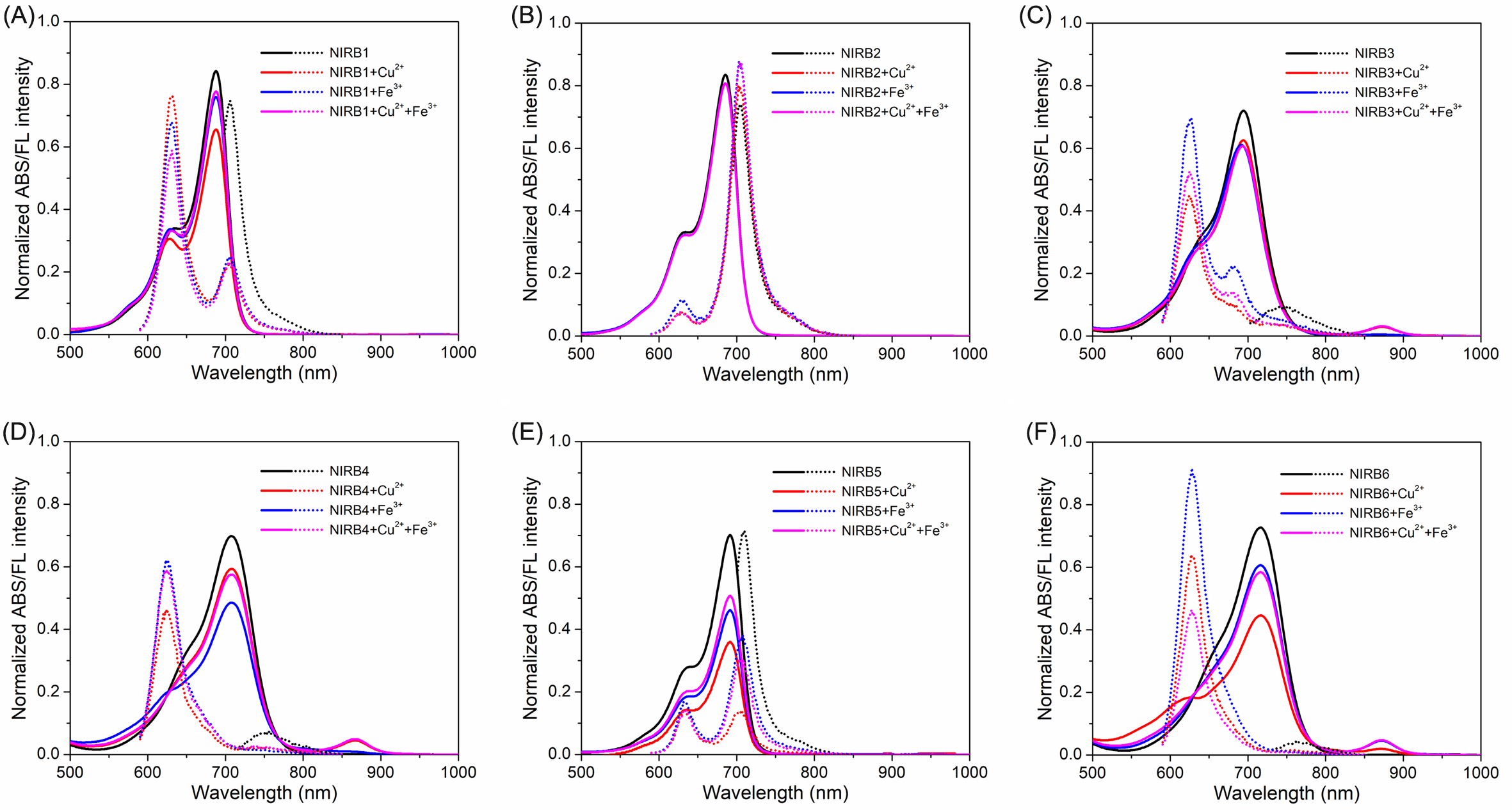


**Figure S34.** Absorption and fluorescence spectra of the molecules (**NIRB1**−**NIRB6**, 10 μM) in ethanol and H_2_O mixture (v/v = 2:1, containing 2% PEG400) in the absence and presence of CuCl_2_/FeCl_3_ (50 μM), the emission of **NIRB1-NIRB6** was exciting at the longest absorption wavelength and the emission of **NIRB1-NIRB6** in the presence of CuCl_2_/FeCl_3_ was exciting at 580 nm. Solid lines and dotted lines represent the absorption and fluorescence spectra, respectively.


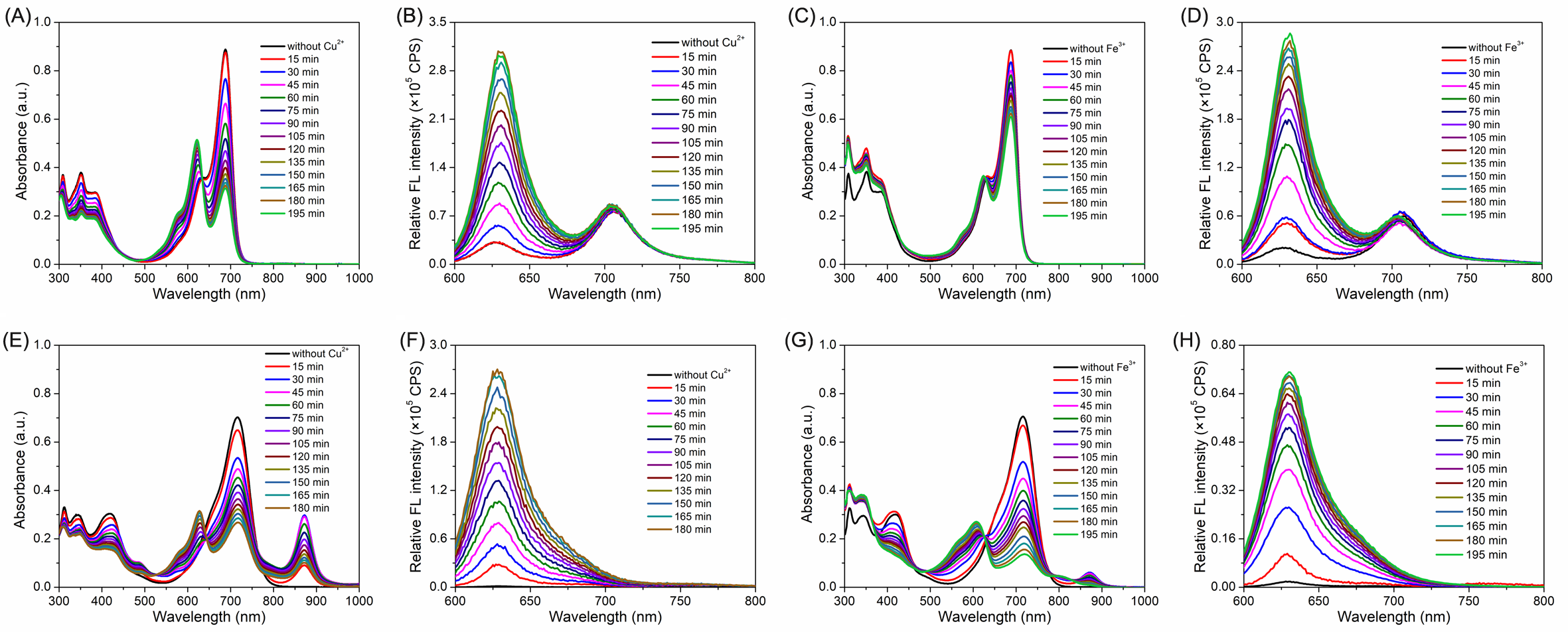


**Figure S35.** (A,E) Time dependent absorption spectra of 10 μM **NIRB1** and **NIRB6** adding with CuCl_2_ (50 μM) in ethanol and H_2_O mixture (v/v = 2:1, containing 2% PEG400). (B,F) Time dependent fluorescence spectra of 10 μM **NIRB1** and **NIRB6** adding with CuCl_2_ (50 μM) in ethanol and H_2_O mixture (v/v = 2:1, containing 2% PEG400), λ_ex_ = 580 nm. (C,G) Time dependent absorption spectra of 10 μM **NIRB1** and **NIRB6** adding with FeCl_3_ (50 μM) in ethanol and H_2_O mixture (v/v = 2:1, containing 2% PEG400). (D,H) Time dependent fluorescence spectra of 10 μM **NIRB1** and **NIRB6** adding with FeCl_3_ (50 μM) in ethanol and H_2_O mixture (v/v = 2:1, containing 2% PEG400), λ_ex_ = 580 nm. Time interval is 15 min.


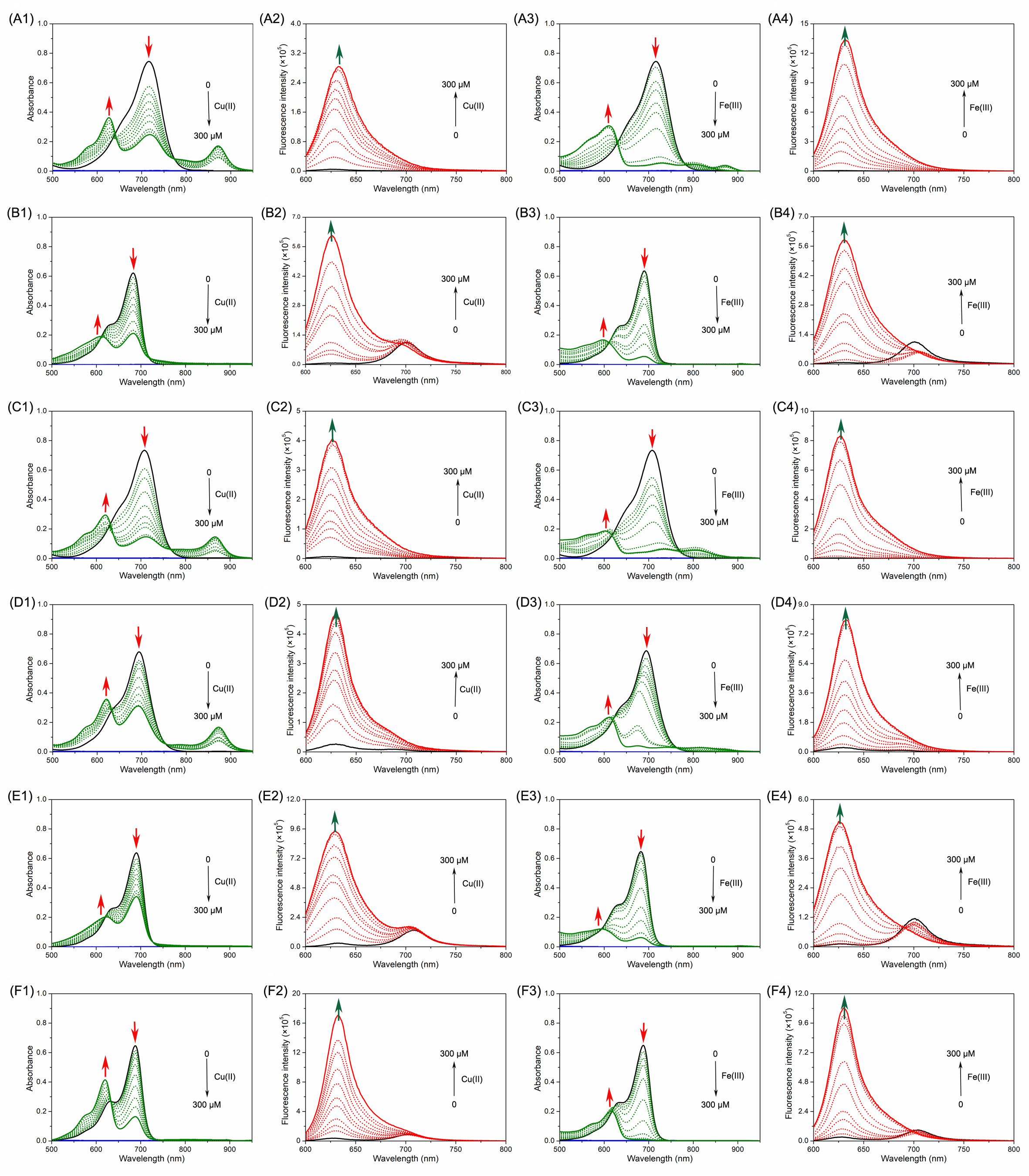


**Figure S36.** Absorption and fluorescence spectra of **NIRB1-NIRB6** (10 μM) towards different concentrations of CuCl_2_ and FeCl_3_ (0-300 μM), excitation: 580 nm.


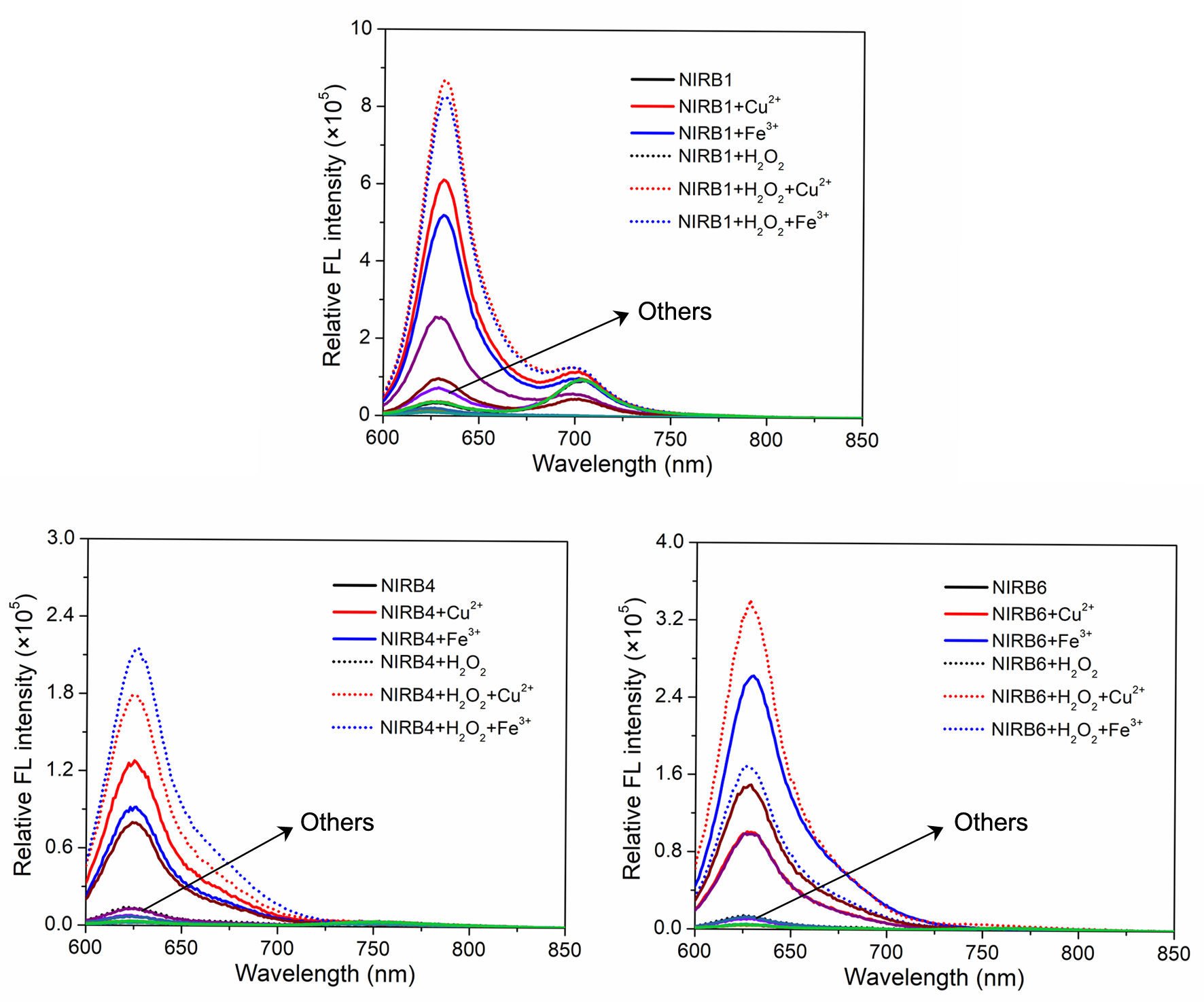


**Figure S37.** Fluorescent spectra of **NIRB1**, **NIRB4** and **NIRB6** (10 μM) upon addition of Cu(II) or Fe(III) (100 μM) with or without ROS/RNS and biothiols in ethanol and H_2_O mixture (v/v = 2:1, containing 2% PEG400). λ_ex_ = 580 nm. (100 μM, H_2_O_2_, ClO^-^, ONOO^-^, Cysteine = Cys and Glutathione = GSH).


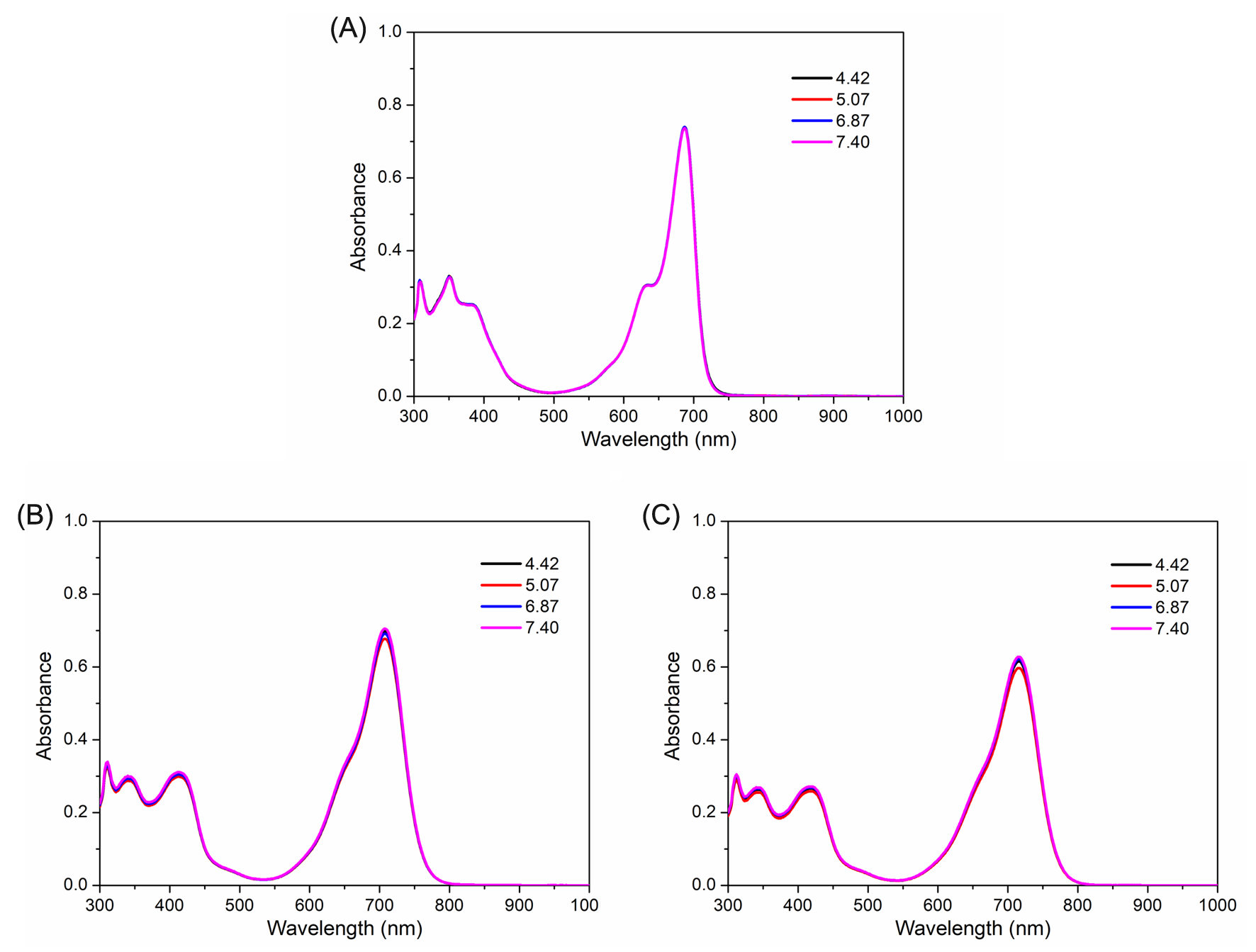


**Figure S38.** Effect of 10 μM **NIRB1** (A)**, NIRB4** (B) and **NIRB6** (C) in PBS buffer solution with different pH values (v/v = 1:1). pH value: 4.42, 5.07, 6.87, 7.40.


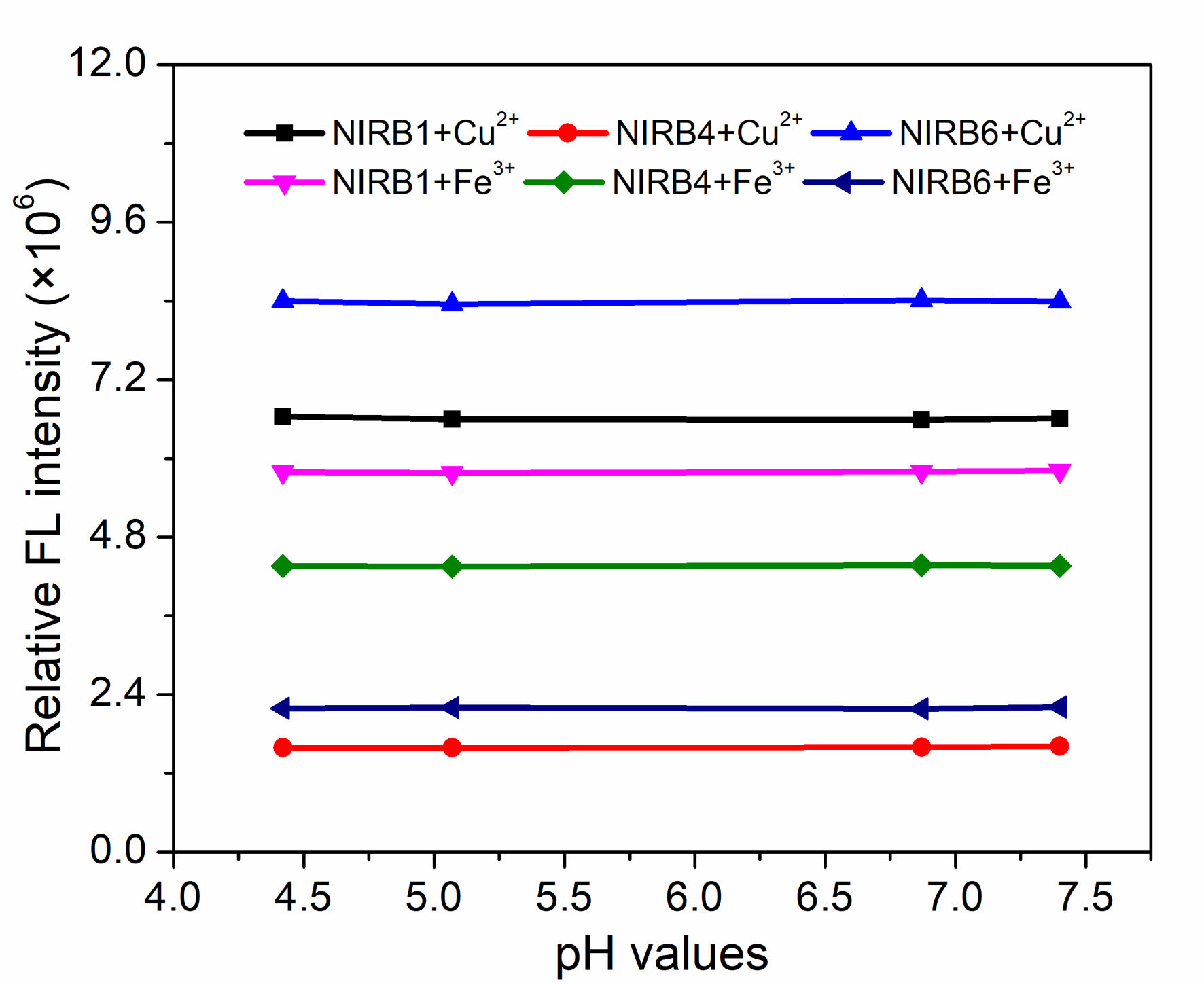


**Figure S39.** Effect of **NIRB1**, **NIRB4** and **NIRB6** (10 μM) adding with Cu(II) or Fe(III) (50 μM) in the mixture of ethanol and PBS buffer solution with different pH values (v/v = 1:1). The fluorescence emission changes at 628 nm (**NIRB1**), 630 nm (**NIRB4**) and 635 nm (**NIRB6**) with the pH titration curve, pH value: 4.42, 5.07, 6.87, 7.40 (from left to right), λ_ex_ = 580 nm.


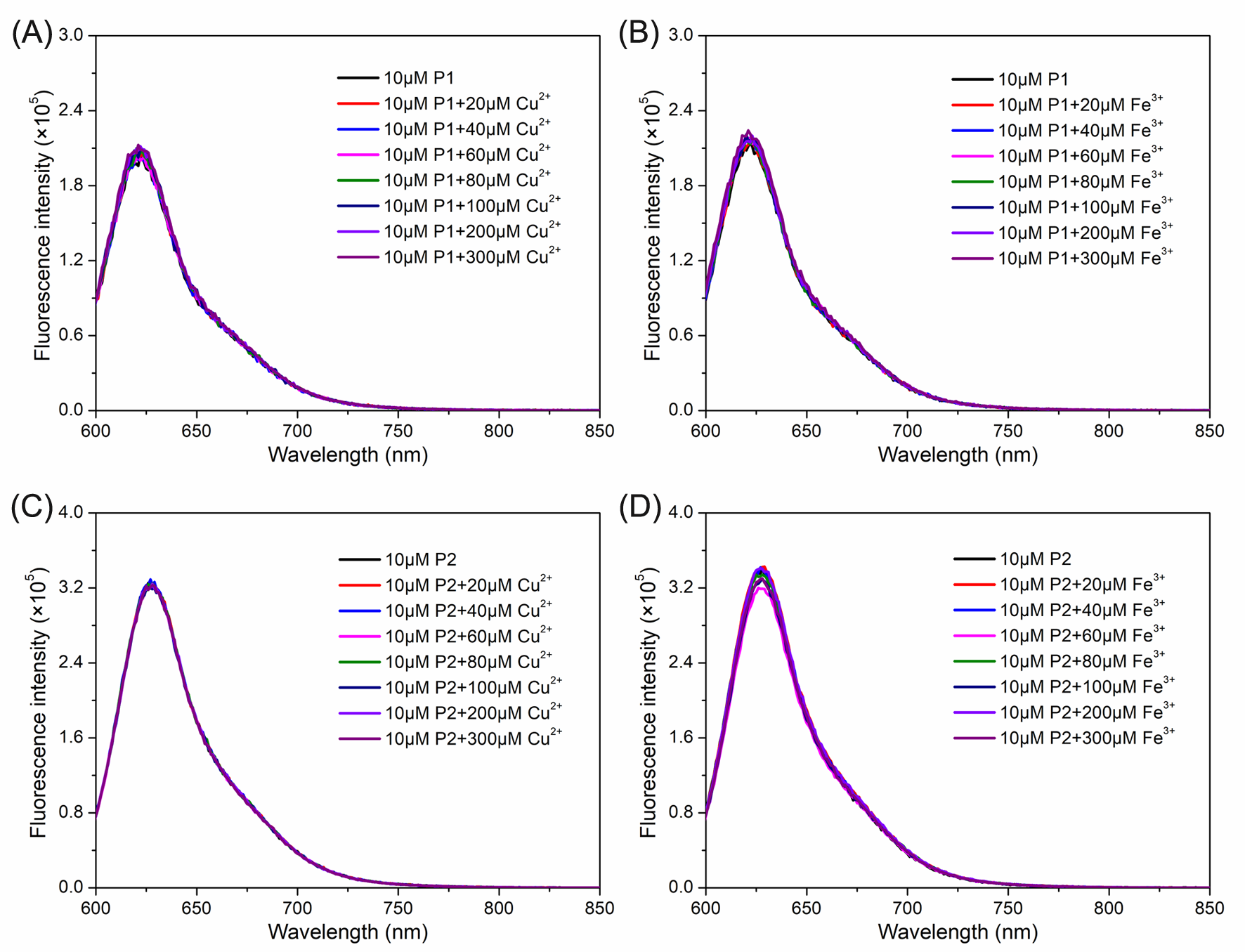


**Figure S40.** Fluorescence spectra of red emitting products **P1** and **P2** (10 μM) in the addition of different concentration of CuCl_2_ and FeCl_3_ from 0 to 300 μM, λ_ex_ = 580 nm.


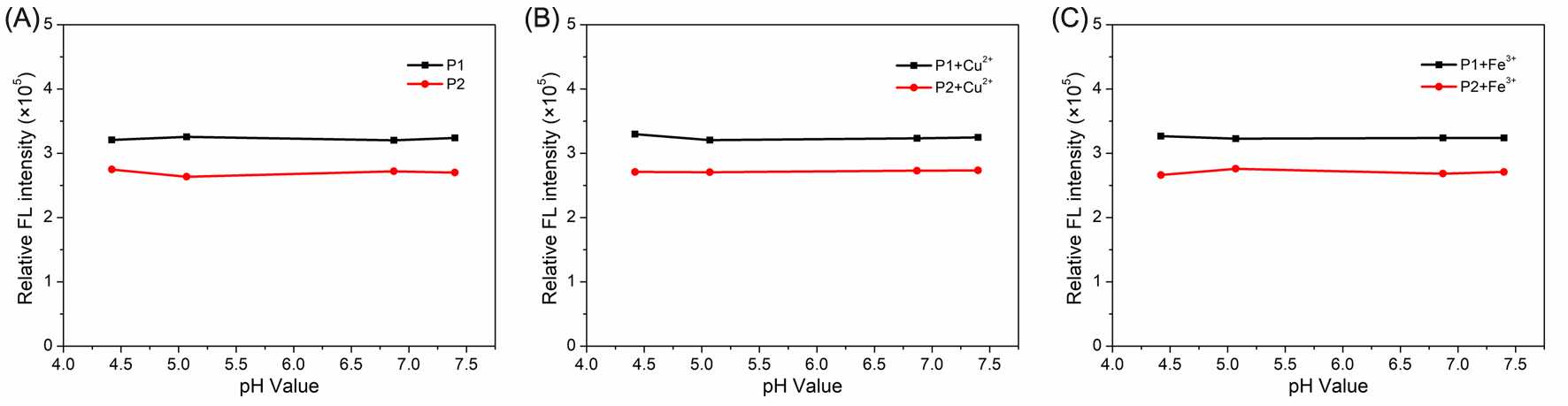


**Figure S41.** Effect of red emitting products **P1** and **P2** (10 μM) in the presence and absence of Cu(II) or Fe(III) (50 μM) in PBS buffer solution with different pH values. The fluorescence emission changes at 641 nm (**P1**) and 625 (**P2**) with the pH titration curve, pH value: 4.42, 5.07, 6.87, 7.40 (from left to right), λ_ex_ = 580 nm.


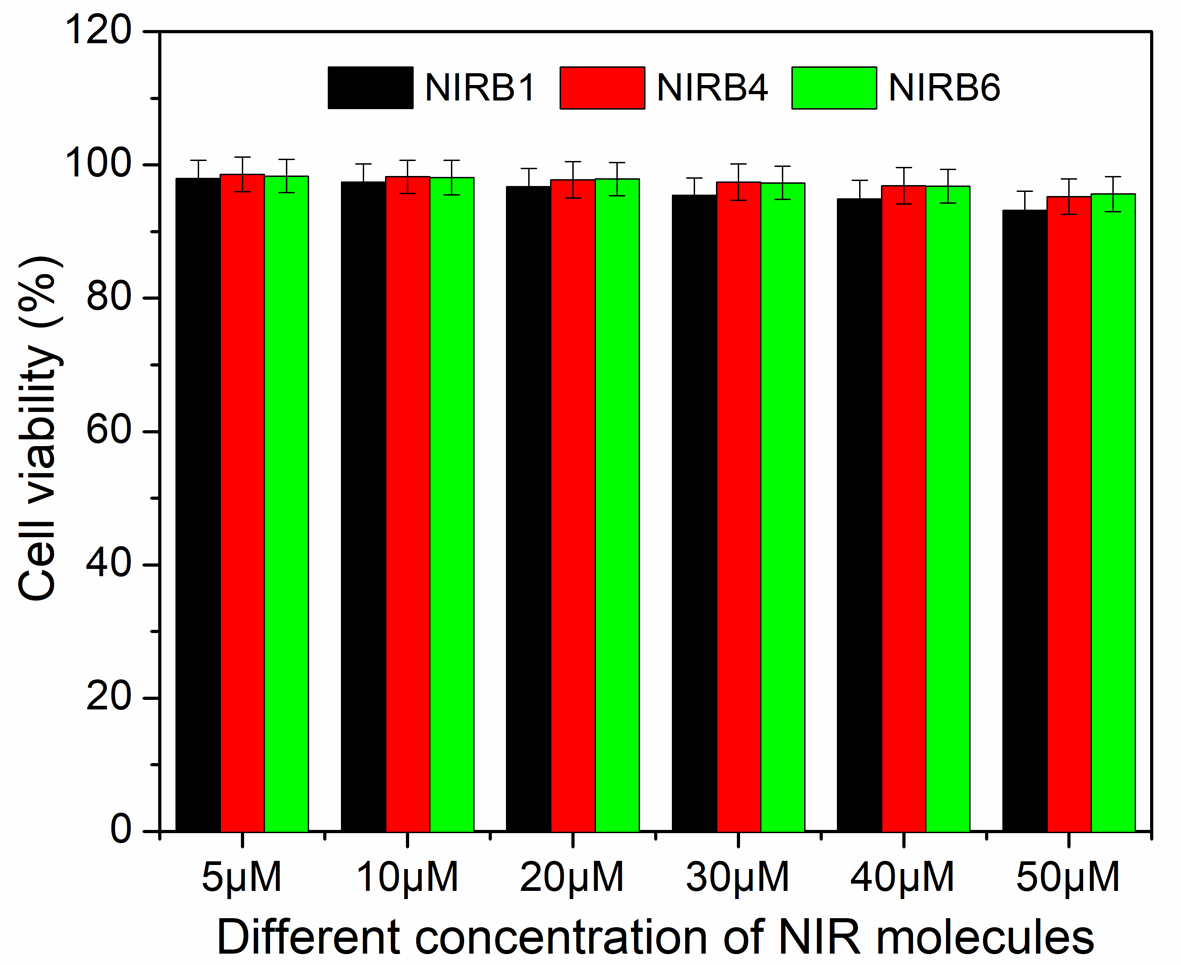


**Figure S42.** Viabilities of U87 cells after incubation with different concentrations of **NIRB1, NIRB4** and **NIRB6** for 24 h.


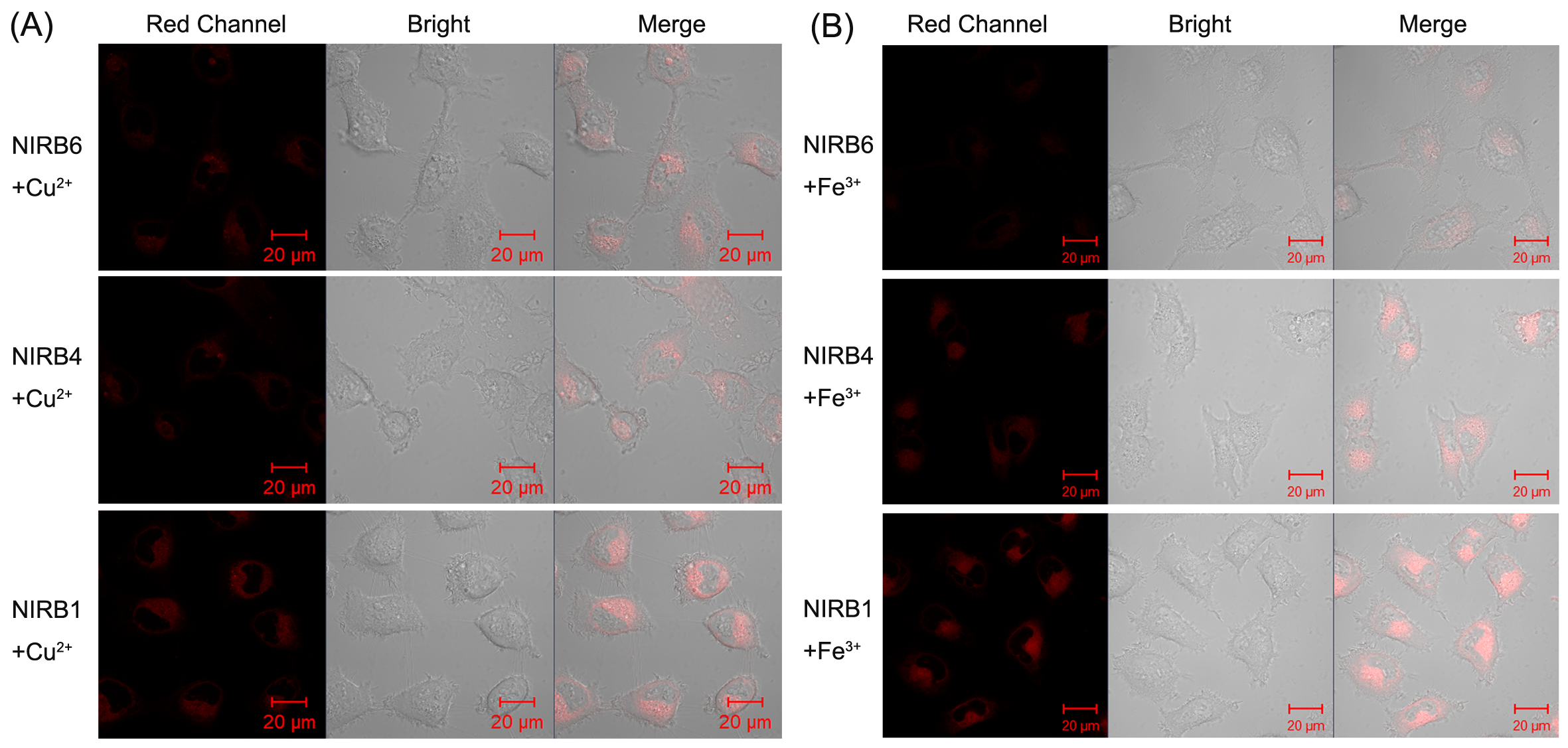


**Figure S43.** (A) Confocal images of Cu(II)-catalyzed reaction of **NIRB1**, **NIRB4** and **NIRB6** (10 μM) in U87 cells, exogenous CuCl_2_ (10 μM), scale bar: 20 μm. (B) Confocal images of Fe(III)-catalyzed reaction of **NIRB1**, **NIRB4** and **NIRB6** (10 μM) incubation in U87 cells, exogenous FeCl_3_ (10 μM), scale bar: 20 μm.


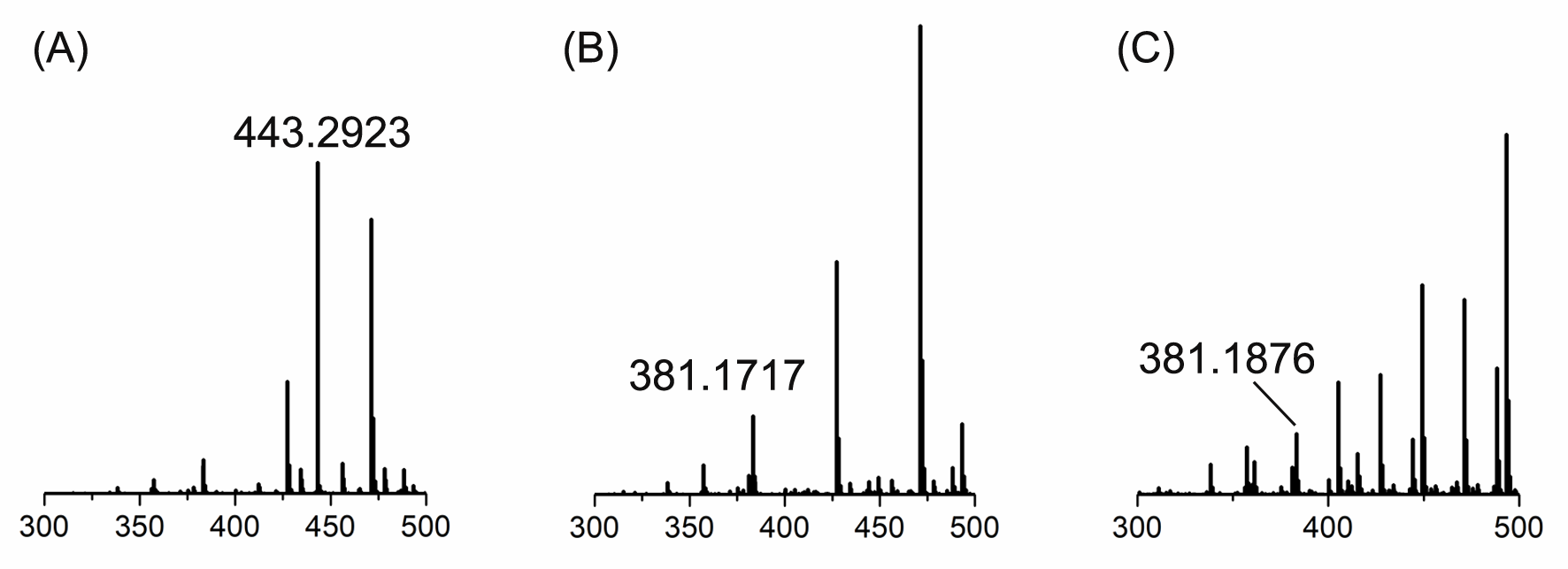


**Figure S44.** MS spectra of the extraction of U87 cells treated with CuCl_2_ (100 μM) and 50 μM **NIRB1** (C), **NIRB4** (B), and **NIRB6** (A), respectively.


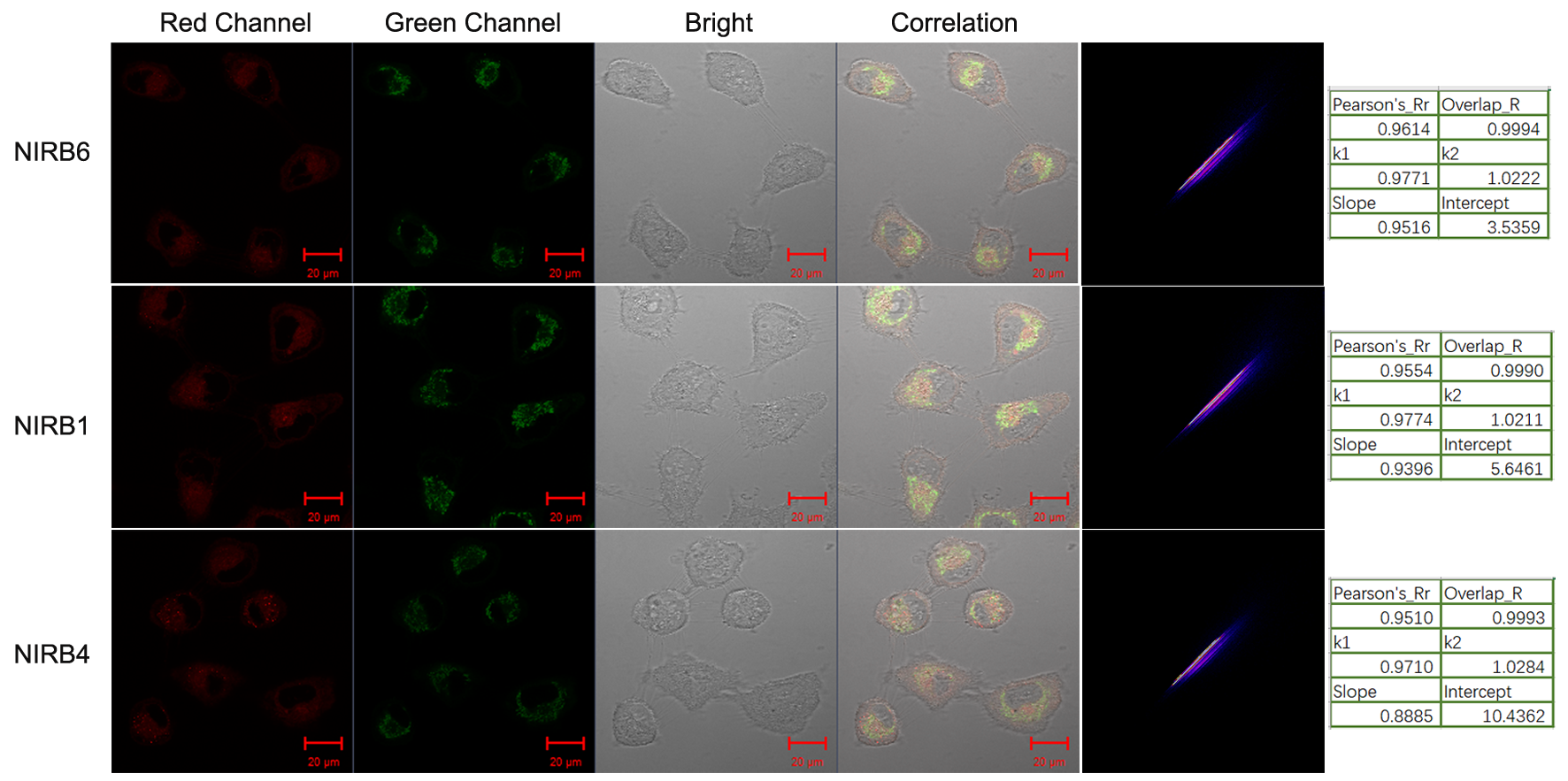


**Figure S45.** Co-localization experiments of 10 μM **NIRB1**, **NIRB4** and **NIRB6** with the addition of 10 μM CuCl_2_ and Mito Tracker Green FM in U87 cells. scale bar: 20 μm.


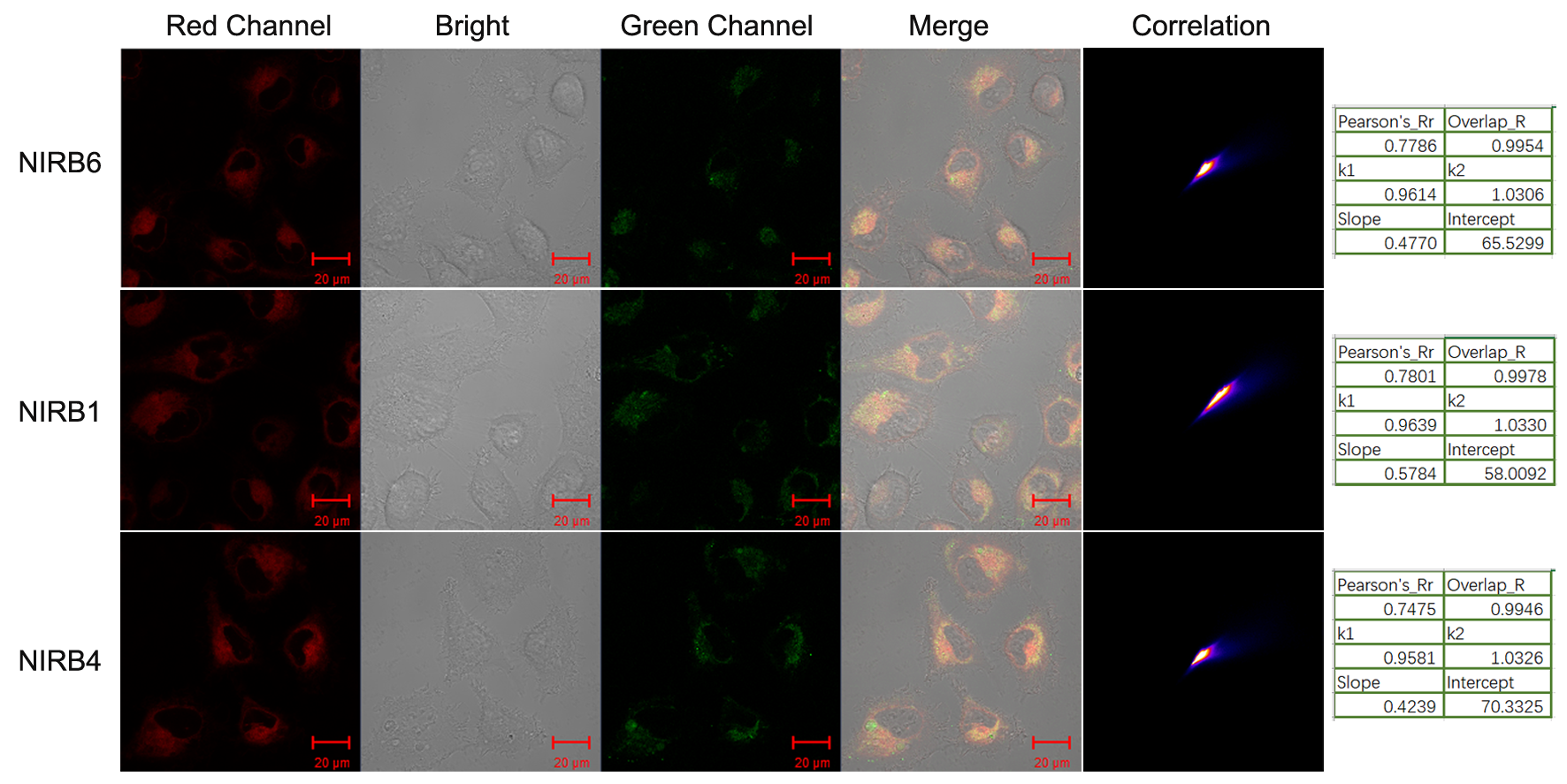


**Figure S46.** Co-localization experiments of 10 μM **NIRB1**, **NIRB4** and **NIRB6** with the addition of 10 μM FeCl_3_ and Mito Tracker Green FM in U87 cells. scale bar: 20 μm.


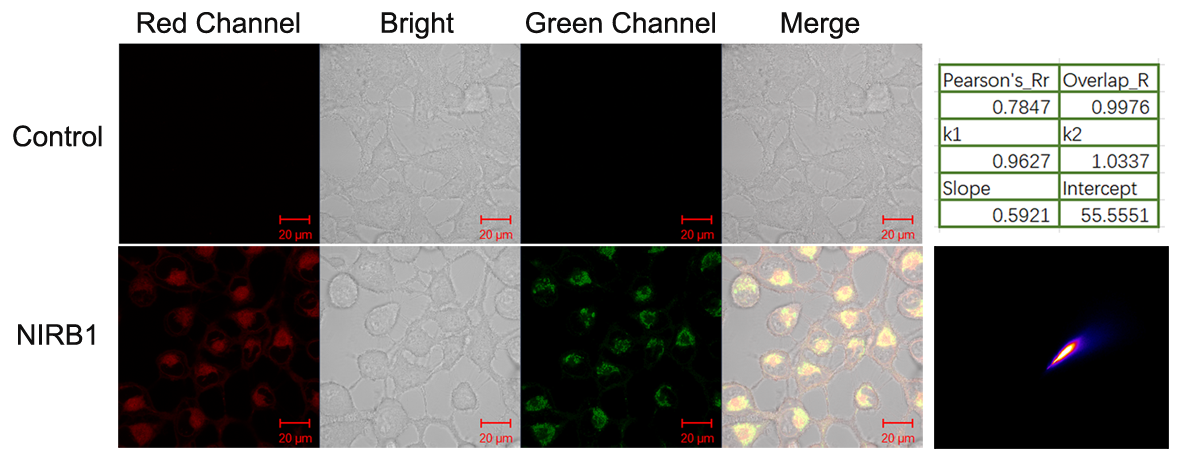


**Figure S47.** Co-localization experiments of 10 μM **NIRB1** and Mito Tracker Green FM in U87 cells. scale bar: 20 μm.


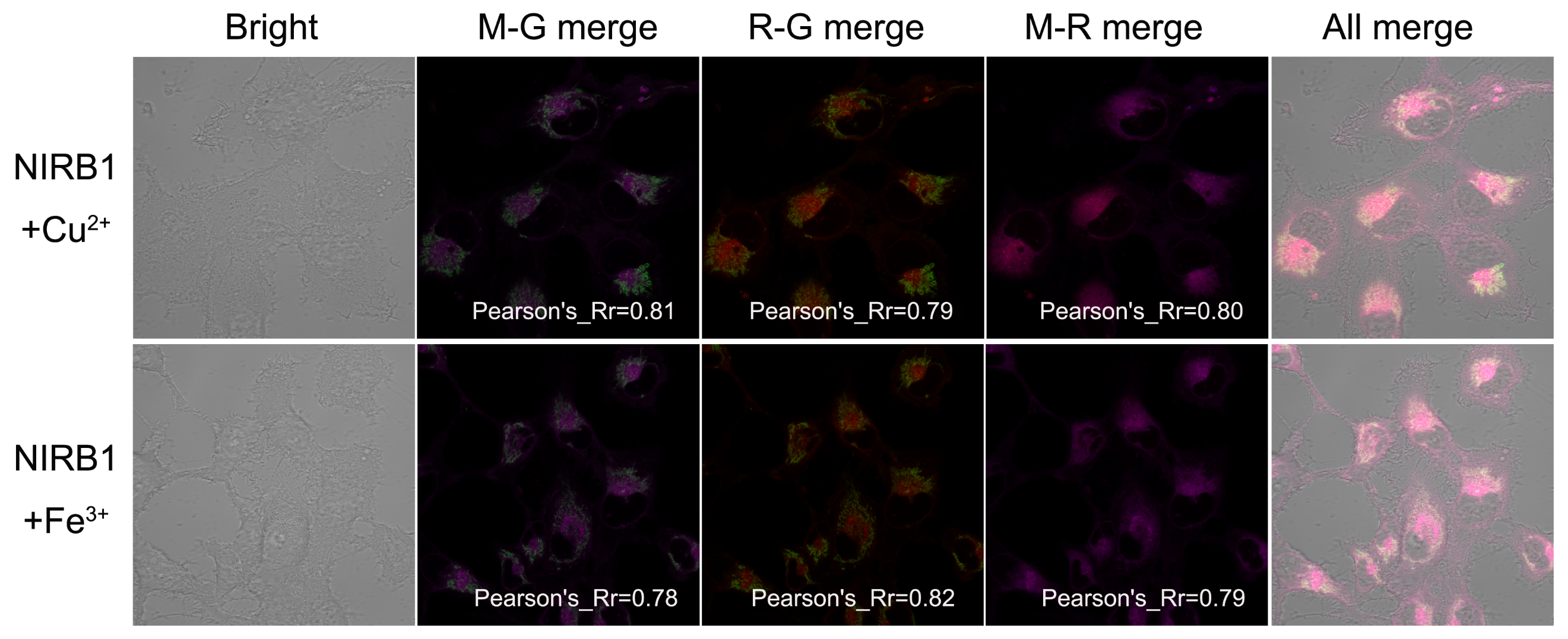


**Figure S48.** Co-localization experiments of 10 μM **NIRB1** and Mito Tracker Green FM in U87 cells after co-incubating with CuCl_2_/FeCl_3_. scale bar: 20 μm.


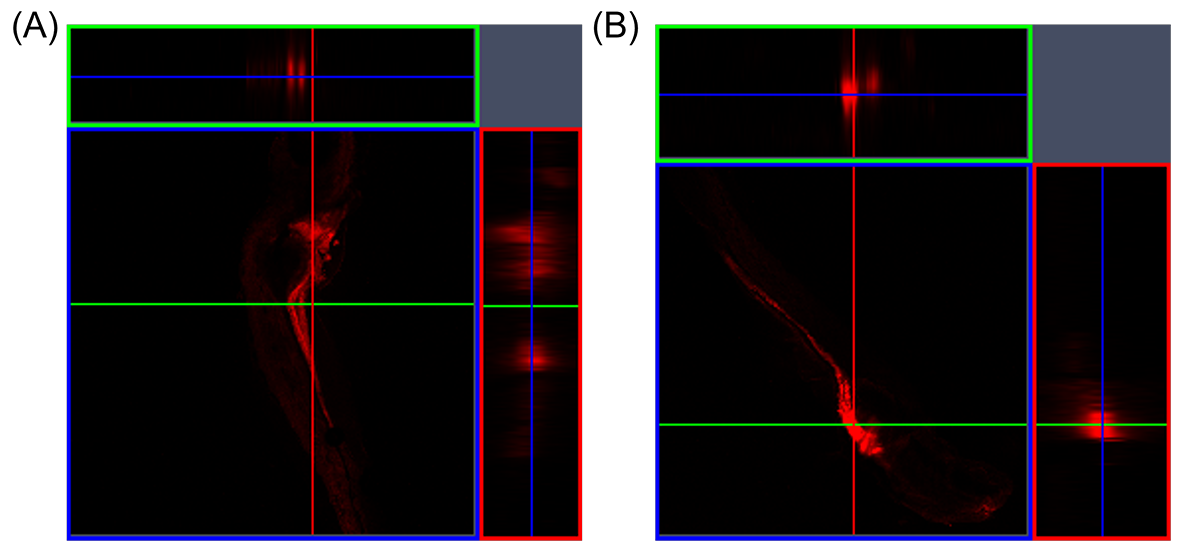


**Figure S49.** Z-stack images of **NIRB1** with CuCl_2_ (A) and FeCl_3_ (B) in zebrafish.


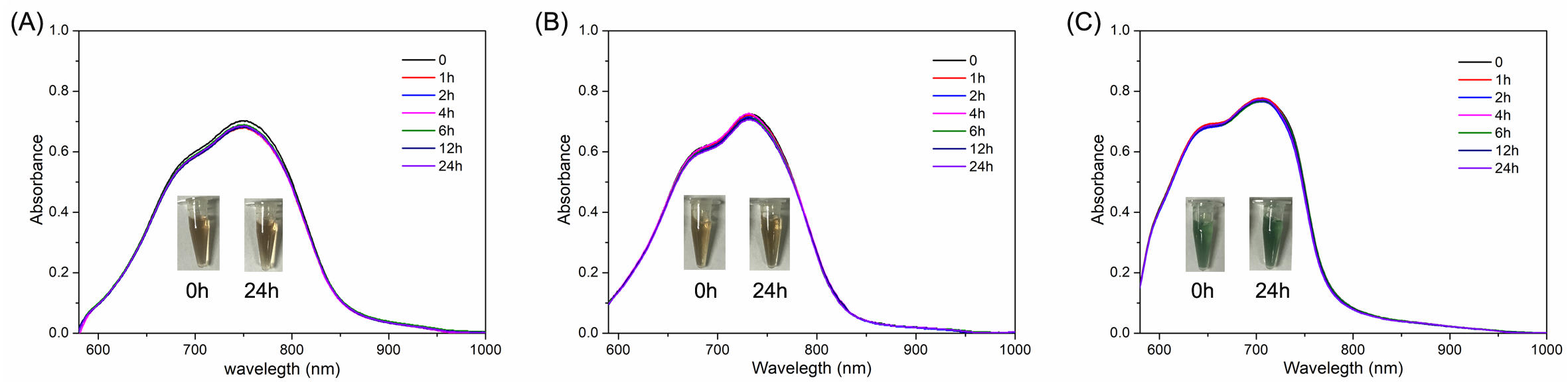


**Figure S50.** UV-Vis spectra of 10 μM **NIRB1** (C), **NIRB4** (B) and **NIRB6** (A) in mouse blood serum, respectively. Insert is images of **NIRB1**, **NIRB4** and **NIRB6** incubate with mouse serum for 0 (left) and 24 h (right).


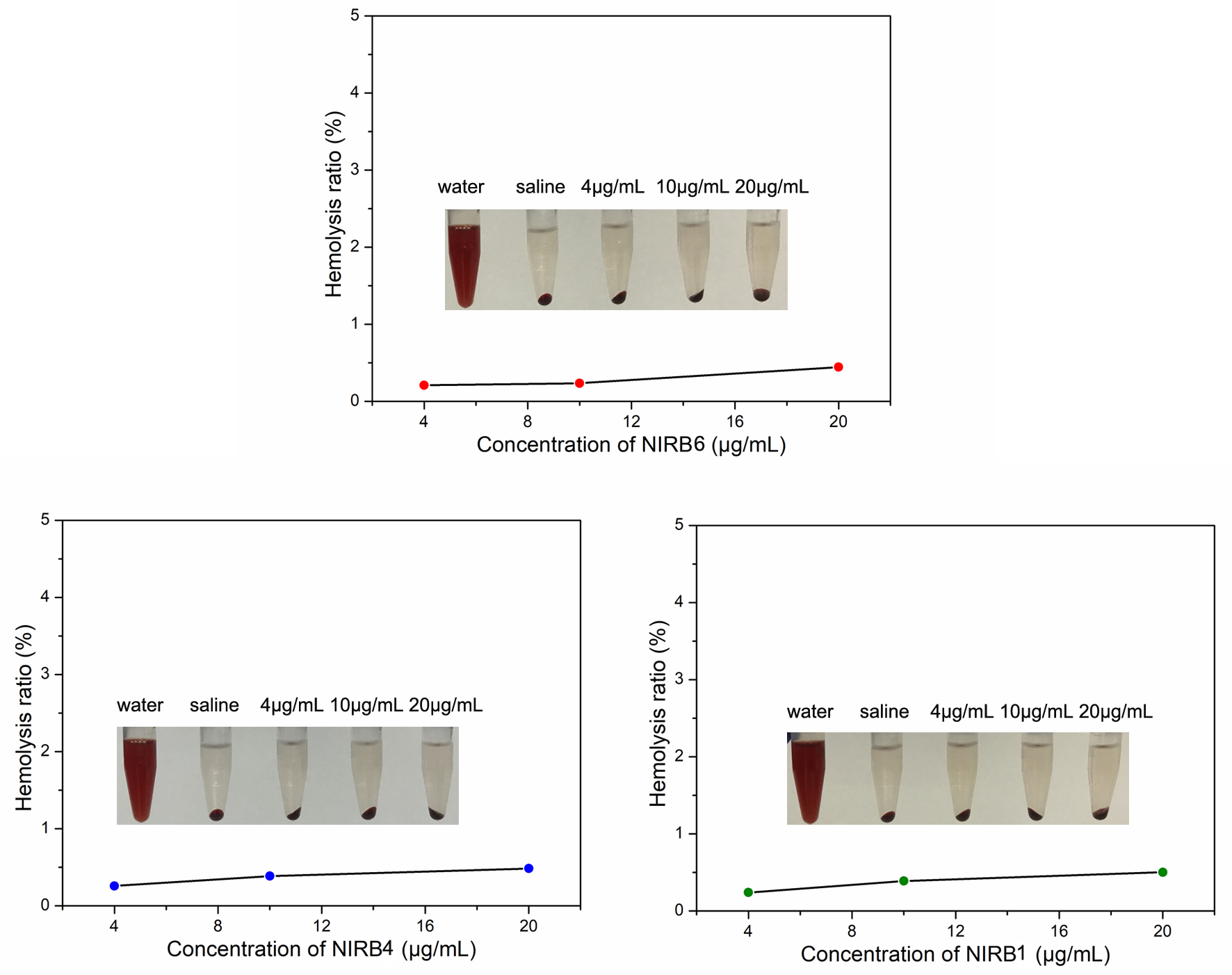


**Figure S51.** Hemolytic analysis of red blood cells treated with different concentration of **NIRB1**, **NIRB4** and **NIRB6** (4 μg/mL, 10 μg/mL and 20 μg/mL) after 3 h. Water and saline as control group for positive and negative experiment. Inserted picture rep-resented the hemolysis results.


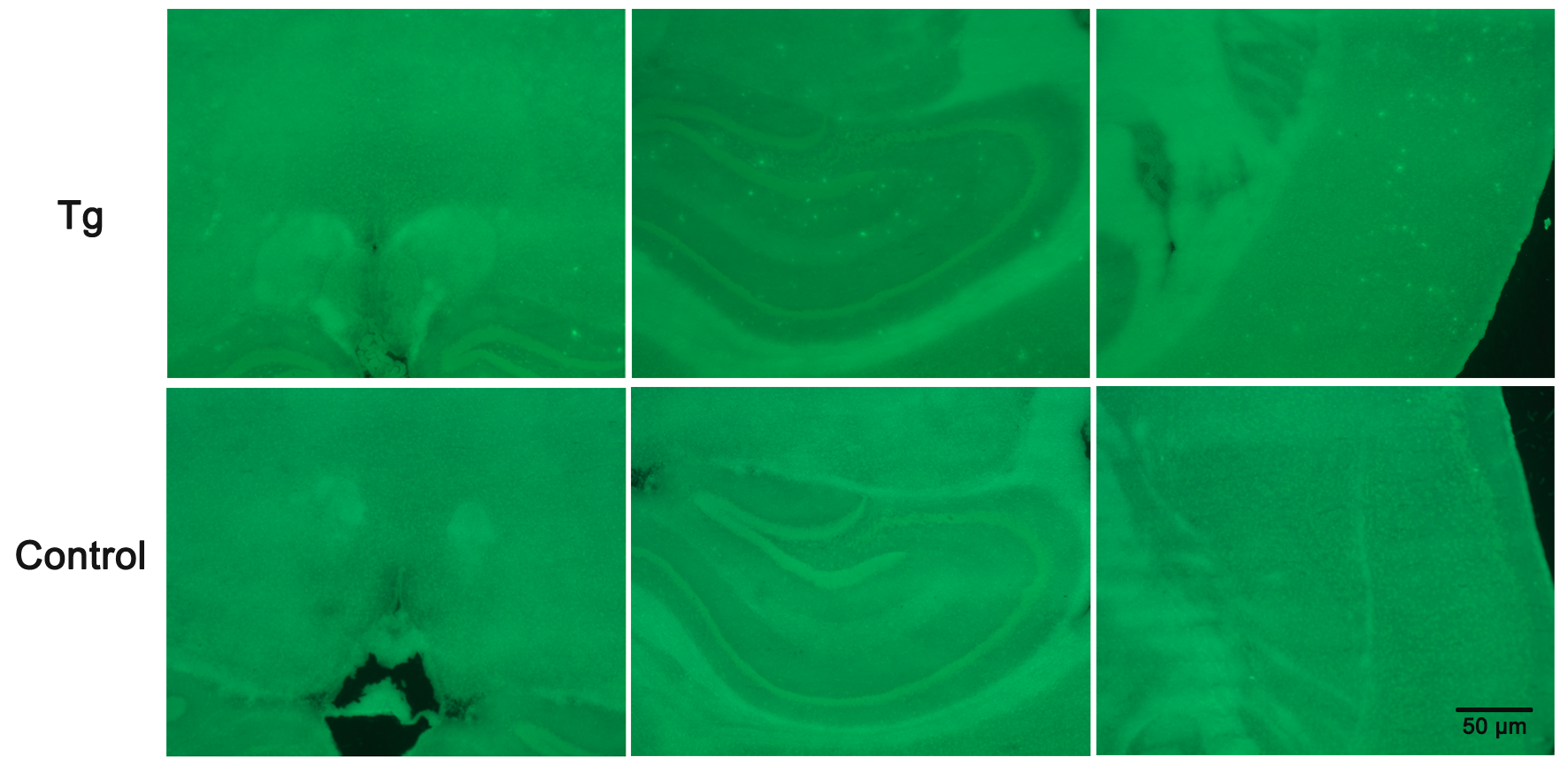


**Figure S52.** Thioflavine-S staining of the brain slices in different regions from Tg mouse and WT mouse, positive staining was mainly localized in the hippocampus and cerebral cortex. Scale bar: 50 μm.
